# Bentonite clay with different nitrogen sources can effectively reduce nitrate leaching from sandy soil

**DOI:** 10.1371/journal.pone.0278824

**Published:** 2022-12-22

**Authors:** Zahid Hussain, Tang Cheng, Muhammad Irshad, Riaz Ahmed Khattak, Chen Yao, Di Song, Muhammad Mohiuddin

**Affiliations:** 1 Yunnan Key Laboratory of Pollution Process and Management of Plateau-Lake -Watershed, Yunnan Research Academy of Eco-Environmental Sciences, Kunming, China; 2 Department of Environmental Sciences, CUI, Abbottabad Campus, Abbottabad, Pakistan; 3 The Brains Institute, Peshawar, Pakistan; 4 Yunnan Infrastructure Investment Co. Ltd., Kunming, China; 5 Department of Environmental Sciences, Kohsar University, Murree, Pakistan; Government College University Lahore, PAKISTAN

## Abstract

Nitrate (NO_3_^-1^) leaching from soils results in the lower soil fertility, reduced crop productivity and increased water pollution. The effects of bentonite clay mixed with various nitrogen (N) fertilizers on NO_3_^-1^ leaching from sandy soils haven’t been extensively studied. Therefore, the present lysimetric study determined NO_3_^-1^ leaching from bentonite [0, 2 and 4% (m/m)] treated sandy soil under three N sources (calcium nitrate [Ca(NO_3_)_2_], ammonium chloride [NH_4_Cl], and urea [CO(NH_2_)_2_] at the rate of 300 kg N ha^-1^). Results showed that bentonite markedly reduced NO_3_^-1^ release in the leachate, while 4% bentonite retained higher NO_3_ in the soil. The NO_3_^-1^ leaching from sandy soil varied with N sources as Ca(NO_3_)_2_ > NH_4_Cl > (CO(NH_2_)_2_. At early stages of leaching, higher concentrations of NO_3_^-1^ were detected in leachate with both NH_4_Cl and Ca(NO_3_)_2_, but leaching of NO_3_^-1^ increased with urea at later leaching stages. The amount of total NO_3_^-1^ retained in soil was conversely related to the amount of NO_3_^-1^ in the leachate. This study indicated that soil amendment with bentonite could efficiently mitigate NO_3_^-1^ leaching from sandy soil and hence prevent N fertilizer losses and groundwater pollution.

## 1. Introduction

Growing populations and changing diets require an increase in agricultural production which may lead to an increase in the use of fertilizers. As such, food demand and fertilizer use have been forecasted to double or triple by 2050 [1]. Chemical N fertilizers and organic manures are often applied to the soil in higher amounts for higher agriculture production, which may lead to the N losses due to removal from the cropped fields into the water bodies [2] and/or emission into the atmosphere [3, 4]. Of the applied N for crops, only 40–50% is being incorporated into the agricultural products [5] and the remainder N is subjected to the substantial losses [6]. The rainfall intensity and irrigation water influence the NO_3_^-1^ loss in the soil profile [7, 8]. The N fertilizers, used either as urea or ammonium form, are biochemically converted to NO_3_^-1^ which is susceptible to leaching from soil-plant system and enter groundwater bodies [2]. Therefore, an effective technology is required to prevent NO_3_^-1^ losses from sandy soils.

Soil NO_3_^-1^ is originated from both organic and inorganic N sources. Leaching and drainage studies found that NO_3_^-1^ is the major form of N occurred in the soil water [8–10]. A number of factors including plant characteristics, seasonal and climatic changes, and soil properties govern NO_3_^-1^ leaching from soils [11, 12]. The specific factors include soil texture, soil N concentration, amount of applied N, type of fertilizer, precipitation amount and intensity, soil water holding capacity, types of crops, root length and N demand of next crop [13, 14]. Leaching of NO_3_^-1^-N is more common than leaching of NH_4_-N since both NO_3_^-1^-N and soil are negatively charged [15]. Over application or un-timely application of animal manures or commercial N fertilizers result in the nutrient imbalance in soils which lead to the increased N leaching rates, especially of NO_3_^-1^, into groundwater [12, 16]. Sandy soils, due to low water holding capacity [17], allow NO_3_^-1^ to leach down into the groundwater faster than the soils having fine textures, such as clay loams [13, 15]. Thus, leaching of NO_3_^-1^ through soil profile can potentially contaminate surface and groundwater [18]. Sandy soils with low organic matter may facilitate leaching of 10–15 mg L^-1^ of NO_3_^-1^ to groundwater [19]. About 20–25% of this NO_3_^-1^ may enter surface water via buffer streams and wetlands causing eutrophication of water bodies [20, 21].

The increasing unsustainable agricultural use of N fertilizers results in NO_3_^-1^ leaching into ground waters [22, 23] and runoff into surface water ecosystem producing unfavorable consequences [24], which adversely affect water quality [22, 25]. The increasing potential of contamination of water resources is linked with the inefficient management of N fertilizer when compared with the natural systems [26–28]. The concentration of NO_3_^-1^ above 10 mg L^-1^ in drinking water are considered as harmful for human health [29]. Higher NO_3_^-1^ consumption has been affiliated with various illnesses, e.g., methemoglobinemia has been proven due to ingestion of over nitrate concentrations in water [30, 31]. The endogenous NO_3_^-1^ may chemically be transformed to carcinogenic N compounds leading to adverse effects of colorectal cancer [32] and bladder cancer [33]. Therefore, developing an effective technology to retain nutrients in soils is imperative to prevent NO_3_^-1^ leaching from soils. Soil amendments have been considered as management practice to reduce NO_3_^-1^ losses from sandy soils [34]. Bentonite, an alumina-siliceous clay material, has not been previously utilized to control NO_3_^-1^ leaching.

Bentonite, like other clays, are hydrous aluminosilicates with fine colloids of < 2 mm of soils [35]. Clays are composed of fine-grained clays minerals and crystals such as quartz, carbonates and oxides [35] and are considered to retain contaminants by anion and cation exchange processes and prevent leaching into groundwaters. Due to effective adsorption capacities, bentonite clay has been used for multiple purposes. Bentonite is also used to remove dyes, radioactive waste, purification of viral RNA and wastewater [36–38]. Bentonite application as amendment enhanced soil fertility by increased soil carbon and potassium [39], while improved water holding capacity of sandy soils under drought stress [40]. Bentonite application to sandy acidic soil improved soil fertility by increasing availability of macro-nutrient (up to 30%) to plants [41]. Fertilizers, if used in combination with nano-dimensional adsorbents increase nutrient use efficiency and reduce nutrient leaching into groundwaters [42], Clay amended sandy soil significantly reduced N and P leaching by 20% to 60% [43]. Leaching of NH_4_-N was reduced by 70% from a mixture of biochar, urea and bentonite plus sepiolite clay [44]. However, it is still unclear that how the type of fertilizer and application of bentonite clay to soils can mitigate NO_3_^-1^ leaching.

A reduction in the NO_3_^-1^ leaching was expected when clay material was applied to the soil. Reports evaluating the interactive effects of bentonite material and N sources on the reduction of NO_3_^-1^ leaching from sandy soils are scanty. Therefore, the objective of the present study was to investigate the influence of bentonite on NO_3_^-1^ leachability from a sandy soil after application of calcium nitrate [Ca(NO_3_)_2_], ammonium chloride [NH_4_Cl], and urea [CO(NH_2_)_2_] as three N sources.

## 2. Materials and methods

### 2.1 Lysimeter experiment

**Fig 1:**
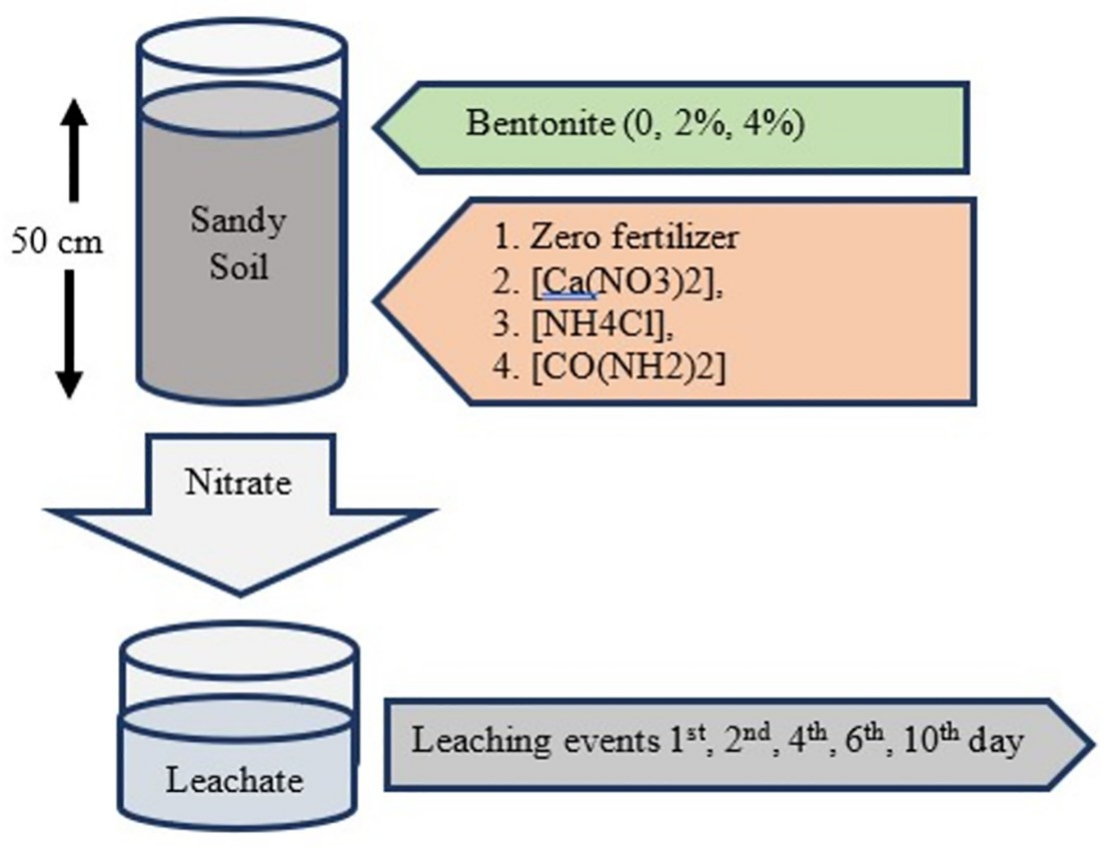
Graphical display of lysimeter experiment.

A leaching experiment was conducted in the Soil Science Laboratory at COMSATS University Islamabad, Abbottabad Campus, Pakistan following the idea of Zhao et al. [45]. For this purpose, PVC columns, with 0.60 m length and 0.15 m diameter, were installed to run the experiment. The bottles used for collecting leachate were installed on the floor. Filter papers were placed on porous bottom of the columns to prevent soil leaching. The columns were connected with bottles using small pipes for the collection of leachate. The connecting pipes were kept airtight to prevent evaporation from the leachate bottles. Locally collected sandy soil samples from agricultural land (0–12 cm depth) were utilized for the experiment. Bentonite material was commercially purchased and then air-dried. Both the soil and bentonite were analyzed for physico-chemical properties before the experiment. After air drying, 46 kg of the soil was added to each column (0.50 m length). The air-dried clay was applied at the rate of 0, 2 and 4% to the sandy soil packed in a PVC column. Three treatments of nitrogen (N) fertilizers namely calcium nitrate [Ca(NO_3_)_2_], ammonium chloride [NH_4_Cl], and urea [CO(NH_2_)_2_] were applied to the soil. Based on the bulk density of soil (1.3 g cm^-1^), each fertilizer was applied at 300 kg N ha^-1^. Initially, as the soil was dry, the amount of first water application was kept higher so that enough water may drain out to collect the leachate. For later leaching events, the leaching fraction (LF) was calculated by dividing the drained water by applied water. Then, the tap water was applied at leaching fraction of 0.3~0.4. Leachate was collected within 24 h after each water application. A total of five leaching events were covered, that is, 1^st^, 2^nd^, 4^th^, 6^th^ and 10^th^ day. The graphical display of the experiment is illustrated in [Fig 1].

### 2.2 Laboratory analysis

Chemical analyses of soil, bentonite and tap water were carried out before experiment. Soil samples were air-dried and sieved via 2 mm sieve. The soil and air-dried bentonite material were tested for pH and electrical conductivity (EC) in 1:5 (w/v) soil-water suspensions by a pH meter (Model: HANNA HI 8520) and EC meter (Model: 4320 JENWAY), respectively [46]. The soil was saturated for overnight, weighed and then the water holding capacity (WHC) of the soil was calculated by the difference in the weight of soil [47]. The bulk density of soil was determined using cylindrical cores. The soil sample was weighed and placed in the oven at 105°C for 8 to 12 hours until the weight was constant. Bulk density was then calculated in the same way as described by Grossman and Reinsch [48].

The post-experiment soil was sampled in two layers (0–25 and 25–50 cm) from the PVC columns and was thoroughly mixed. A 10 g soil sample was shaken in 100 mL distilled water for 1 h and then the suspension was filtered. Moisture content in the soil samples was adjusted by oven drying few grams of soil. The leachate collected from 5 events was analyzed for NO_3_ concentration. The NO_3_^-1^ concentration in pre- and post-experiment soil, bentonite and leachate was determined by UV spectrophotometer (Model: LI-UV-7000) at 220 nm [49]. All the reagents/chemicals of Sigma Aldrich, Germany, were utilized during the experiment.

### 2.3 Statistical analysis

Data were statistically analyzed by OriginLab 2021 for graphical presentation. The three-way analysis of variance (ANOVA) was performed on Sigmaplot. The three factors were taken as bentonite (0, 2% and 4%), N fertilizer sources ([Ca(NO_3_)_2_], [NH_4_Cl] and [CO(NH_2_)_2_]), and leaching events (1^st^, 2^nd^, 4^th^, 6^th^ and 10^th^ day) with three replications. A post hoc Tukey test was also performed to determine the significant difference between the levels of factors.

## 3. Results

### 3.1. Pre-experiment chemical analysis

Bentonite clay, soil and water were analyzed for chemical properties before experiment, which are presented in Table 1. Analysis revealed that soil had highest NO_3_^-1^ concentrations, compared to bentonite clay. The tap water had low concentrations of NO_3_^-1^. The electrical conductivity (EC) of bentonite was highest compared to soil and water samples, but still it fell below the category of non-saline (EC<4 dS m^-1^). However, the pH of bentonite was lower (pH<7) making it more acidic as compared to soil and water.

**Table 1:** Nitrate, electrical conductivity (EC) and pH of bentonite clay material, soil and tap water.

Material	Nitrate	EC (mS m^-1^)	pH
Bentonite	23.5 mg kg^-1^	126.7	5.7
Soil	34.4 mg kg^-1^	87.8	7.8
Tap water	7.8 mg L^-1^	37.5	7.2

### 3.2. Effect of bentonite and N sources on NO_3_^-1^ leaching

**Fig 2:**
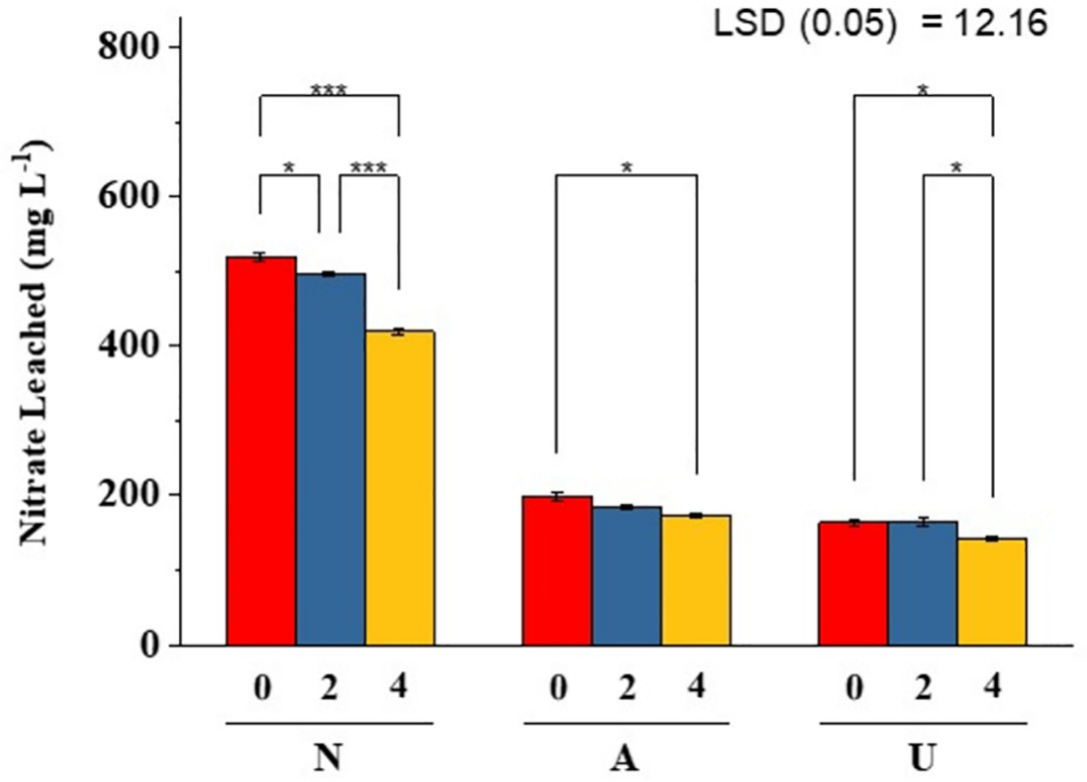
Average amount of leachate nitrate from bentonite treated sandy soil (from day 1 to day 10). N, A and U indicate Ca(NO_3_)_2_, NH_4_Cl and CO(NH_2_)_2_, respectively. Bentonite clay was applied at the rate of 0%, 2% and 4%. *, ** and *** show significant differences at P<0.05, P<0,01 and P<0.001, respectively.

**Fig 3:**
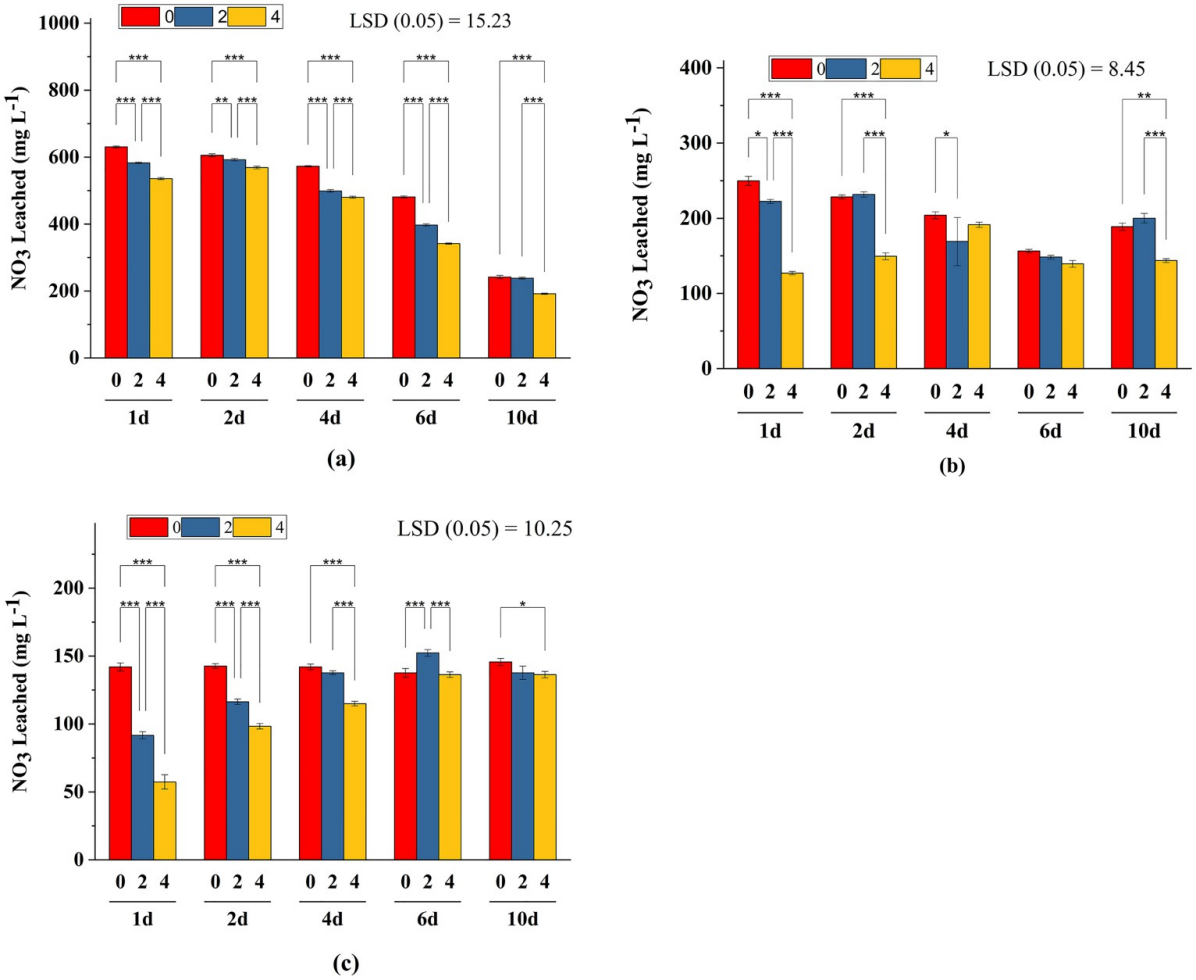
Nitrate leaching in bentonite amended sandy soil using Ca(NO_3_)_2_ (a), NH_4_Cl (b), and CO(NH_2_)_2_ (c) with 0%, 2% and 4% bentonite during five leaching events (days). *, ** and *** show significant difference at P<0.05, P<0,01 and P<0.001, respectively.

**Fig 4:**
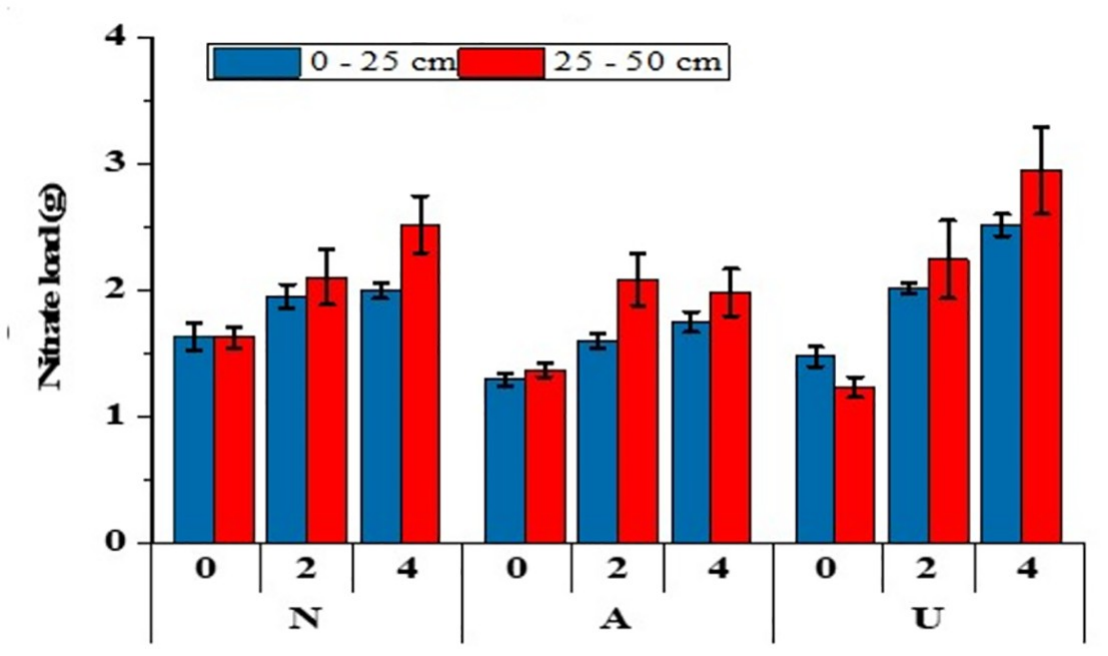
Residual nitrate loads (g) in soil after leaching events (days) at 0%, 2% and 4% bentonite with Ca(NO_3_)_2_ (N), NH_4_Cl (A), and CO(NH_2_)_2_ (U). *, ** and *** show significant difference at P<0.05, P<0,01 and P<0.001, respectively.

**Fig 5:**
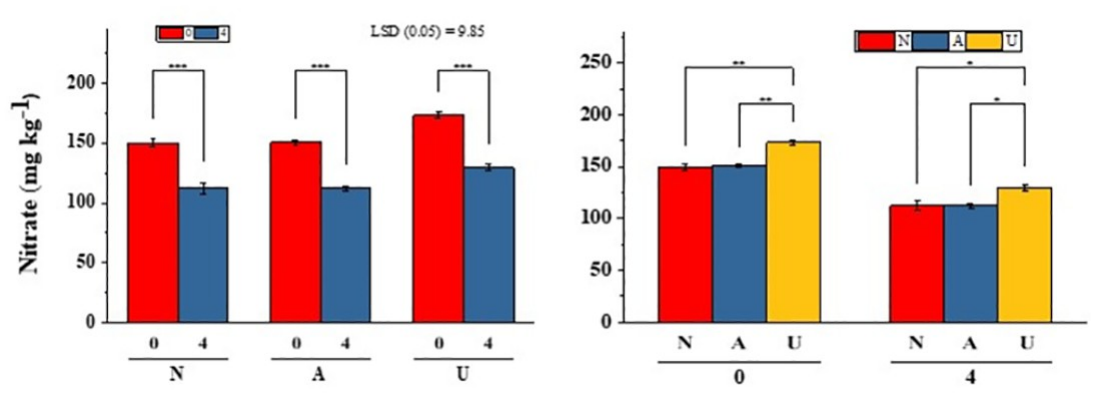
Nitrate concentration in 4% bentonite clay mixed sandy soil after discrete incubation with Ca(NO_3_)_2_ (N), NH_4_Cl (A) and CO(NH_2_)_2_ (U), *, ** and *** show significant difference between treatments at P<0.05, P<0,01 and P<0.001, respectively.

Impact of bentonite clay mixed with different N sources on NO_3_^-1^ leaching is illustrated in [Fig 2]. Results showed NO_3_^-1^ concentration decreased by 12 to 19% in the leachate, irrespective of the source of N with increasing bentonite rates. The significantly highest reduction (20–25%) in NO_3_^-1^ leaching was recorded with Ca(NO_3_)_2_ with 4% bentonite as compared with CO(NH_2_)_2_ and NH_4_Cl at similar bentonite rates. At early stages of leaching, the leachate showed higher concentrations of NO_3_^-1^ in leachate with both NH_4_Cl and Ca(NO_3_)_2_, but leaching of NO_3_ increased with urea sources at later leaching stages [Fig 3]. Total NO_3_^-1^ loads were higher in soil with urea and Ca(NO_3_)_2_ treated soil at 4% bentonite as compared to NH_4_Cl [Fig 4]. The incubation of soil with bentonite (4%) reduced NO_3_ content by 7%, 20% and 8% with Ca(NO_3_)_2_, NH_4_Cl and CO(NH_2_)_2_ treated soil, respectively [Fig 5].

### 3.3. Statistical analysis

A three-way analysis of variance (ANOVA) was performed on NO_3_^-1^ leaching with 3 N sources, 3 levels of bentonite and 5 leaching events [Table 2]. Analysis showed that there was significant difference (P<0.001) within N sources, bentonite levels and leaching events (days). There was statistically significant (P<0.01) interaction among all the factors. The Tukey test revealed significant (P<0.05) difference between 0% and 2%, and 0% and 4% bentonite with all N sources on NO_3_^-1^ leaching, but the difference was not significant between 2% and 4% bentonite with all N sources [Table 2].

**Table 2:** Summary ANOVA on effect of bentonite and N sources on NO3-1 leaching at different leaching events.

Source of Variation	DF	SS	MS	F	P
N sources (N)	2	2943336.13	1471668	14453	< 0.01
Bentonite levels (B)	2	74157.91	37078	364	< 0.01
Leaching event / day (D)	4	282061.82	70515	692	< 0.01
N x B	4	11270.48	2817	27	< 0.01
N x D	8	589355.42	73669	723	< 0.01
B x D	8	15955	1994	19	< 0.01
N x B x D	16	24125	1507	14	< 0.01
Residual	90	9164	101		
Total	134	3949426	29473		

## 4. Discussion

Results showed that irrespective of the source of N, the NO_3_^-1^ leaching consistently decreased with increasing bentonite application showing the sequences of fertilizer type as: Ca(NO_3_) _2_ > NH_4_Cl > CO(NH_2_)_2_ [Fig 2]. Form of N leached from the soil columns was closely related to the type of fertilizer applied to the soil [50]. The NO_3_^-1^ fertilizers appeared to be more sensitive to the leaching, especially in sandy soils [51] and also to the denitrification [52] as compared to urea or ammonium fertilizers. Also, NO_3_^-1^ leaching is substantially higher in free-drained soils [53], such as sandy soil with macropores used in the present study.

Application of bentonite decreased NO_3_^-1^ leaching, regardless to the rate of application. The [NO_3_^-1^] decreased with increasing bentonite treatments level. Whereby higher NO_3_^-1^ concentration was observed in NO_3_^-1^ and NH_4_ containing fertilizers during the initial leaching [Fig 3]. Urea form of N showed consistent increases in NO_3_^-1^ concentrations in water collected in later leaching stages [Fig 3]. This could be associated with increased nitrification process in soils under unsaturated conditions [54], which might have resulted in increased NO_3_^-1^ leaching at later stages. Accumulation of NO_3_^-1^ was more in the soil sampled from the lower layer of the column after a leaching process, showing the sequence as CO(NH_2_)_2_ > NH_4_Cl > Ca(NO_3_)_2_.

The amount of total NO_3_^-1^ retained in soil was termed as nitrate loads conversely related to the amount of NO_3_^-1^ in the leachates [Fig 4]. An enhanced application of bentonite significantly retained NO_3_^-1^ in the soil columns. A higher amount of NO_3_^-1^ was retained in the soil amended with 4% bentonite. The application of bentonite clay enhanced soil moisture and improved macro-aggregate development [55] which improved soil quality through structural development, by increased exchange of anions and cations [56] and helped in reduced leaching while promoting nutrient retention [57].

Enrichment of sandy soils with bentonite increased the porosity and altered the pore-size distribution [58]. The interactions of bentonite with biochar and urea improved soil properties by diffusing soil moisture which controlled the mobility of nutrients within soils [59], thus with high water retaining capacity, increased exchange capacity, swelling, thermal stability and slow-releasing characteristics, bentonite offers valuable solution to reduced nutrient leaching from loose soils.

Higher quantity of NO_3_^-1^ was retained in urea treated soil followed by nitrate and ammonium containing fertilizers [Fig 4]. Such retention could be attributed to the transformation of NO_3_^-1^ in urea contained soil after few days of incubation. Across all N sources, the application of bentonite (4%) markedly limited the release of NO_3_^-1^. After incubation, bentonite contents reduced the magnitude of NO_3_^-1^ among fertilizers as follow: 7% in Ca(NO_3_)_2_, 20% in NH_4_Cl and 8% in urea treated soil [Fig 5]. The soil having negatively charged sites attracts more positively charged NH_4_ as compared to the negatively charged NO_3_^-1^, and therefore the NH_4_ has been considered to be a less mobile in soils, than NO_3_^-1^ [60, 61].

The effect of bentonite on the leachability of NO_3_^-1^ varied with different N fertilizers. The NO_3_^-1^ leaching consistently decreased with increasing bentonite application showing the sequences of fertilizer type as: Ca(NO_3_) _2_ > NH_4_Cl > CO(NH_2_)_2_ [Fig 2]. Addition of Ca as Ca(NO_3_)_2_ increased the adsorptive capacity of bentonite at low pH (5–6), while higher concentration of NO_3_^-1^ ion due to the addition of calcium nitrate as N source resulted in increased adsorption of NO_3_^-1^ at bentonite clay surfaces [62]. The mechanism can be further explained by Ca hydrolysis, which resulted into the formation of less soluble Ca(OH)_2_, releasing more H^+^ ions, thus acidifying the media [Eq 1] [63]. Under low pH, the anion exchange capacity of bentonites in significantly increased which offers more positive sites to attract NO_3_^-1^ on its surface [Eq 2], which increased the retention of NO_3_^-1^ ions on soil colloids due to adsorption phenomenon [Eq 3]. This ultimately reduced NO_3_^-1^ leaching from sandy soil and showed lower NO_3_^-1^ concentrations in leachate. The entire process can be explained as follows, where A is dissociated anion and X is the soil colloidal surface

$${\text{Ca}}^{2+}+{\text{H}}_{2}\text{O}⇌\text{Ca}{\left(\text{OH}\right)}_{2}+2{\text{H}}^{+}$$ (1)

$$\text{X}+{\text{H}}^{+}⇌{\text{X}}^{+}+\text{HA}$$ (2)

$${\text{X}}^{+}+{\text{NO}}_{3}{}^{-}⇌{\text{XNO}}_{3}$$ (3)

Surface charge of variable charge clays varies with changes in pH of soil solution, therefore, at low pH, the anion exchange capacity exceeds cation exchange capacities which retain more NO_3_^-1^ on its surfaces [64], while increasing the mobility of NH_4_ in such conditions. However, the mobility of both N forms can be maintained by adjusting the rates of Ca(NO_3_)_2_ and urea along with bentonite clay under field conditions. The study suggested that bentonite amendment with Ca(NO_3_)_2_ as N sources could effectively mitigate NO_3_^-1^ leaching from sandy soils.

## 5. Conclusions

It is concluded that N sources and bentonite application were important factors affecting the NO_3_^-1^ leaching from sandy soil. Application of N sources enhanced NO_3_^-1^ leaching from the sandy soil. The NO_3_^-1^ leaching decreased in the order of Ca(NO_3_)_2_ > NH_4_Cl > urea. Bentonite substantially reduced NO_3_^-1^ in the leachate. Urea showed higher NO_3_^-1^ at the later leaching events. Higher contents of NO_3_^-1^ were retained in the soil with 4% bentonite. Higher NO_3_^-1^ contents were accumulated in the lower part of the soil column after a leaching process. This experiment suggests that bentonite clay chemistry with added Ca(NO_3_)_2_ provide better understanding of anion exchange capacity to retain higher NO_3_^-1^ concentrations in soil, thereby decreasing NO_3_^-1^ leaching from sandy soil. Further research is suggested to investigate the effects of different clay types on the dynamics of nitrate under field conditions.

## Supporting information

S1 File (PDF)
