# Supplementary material for: Bentonite clay with different nitrogen sources can effectively reduce nitrate leaching from sandy soil
Source: PLoS One. 2022 Dec 22;17(12):e0278824. doi: 10.1371/journal.pone.0278824 (PMC9779043; doi:10.1371/journal.pone.0278824)
Supplement: S1 File — (PDF) [file pone.0278824.s001.pdf]

Figure 2 data: Leachate nitrate (mg L^{-1}) from bentonite treated sandy soil (from day 1 to day 10). Three replications data

N + 0%	N + 2%	N + 4%	A + 0%	A + 2%	A + 4%	U + 0%	U + 2%	U + 4%
510	501	412	208	181	167	159	174	136
518	498	428	196	185	172	173	158	148
529	490	420	189	188	180	162	161	142

*For this and subsequent data: N, A and U indicate $Ca(NO_3)_2$, NH_4Cl and $CO(NH_2)_2$., respectively. Bentonite clay was applied at the rate of 0%, 2% and 4%.

Figure 3 data. Nitrate leaching in bentonite amended sandy soil using N sources during five leaching events' Three replications data

For NH_4Cl treatment					
Bentonite application	1d	2 d	4 d	6 d	10 d
0%	240	224	213	157	198
	248	228	201	152	186
	261	233	198	160	182
2%	217	226	105	147	190
	226	231	199	145	198
	224	238	203	153	212
4%	123	158	198	148	148
	131	148	190	137	144
	127	142	186	133	139

*For this and subsequent data: N, A and U indicate $Ca(NO_3)_2$, NH_4Cl and $CO(NH_2)_2$., respectively. Bentonite clay was applied at the rate of 0%, 2% and 4%.

Figure 3 data. Nitrate leaching in bentonite amended sandy soil using N sources during five leaching events' Three replications' data

For urea [$CO(NH_2)_2$] treatment					
Bentonite application	1d	2 d	4 d	6 d	10 d
0%	147	140	143	143	150
	142	142	138	138	146
	137	146	145	132	141
2%	92	116	138	149	138
	87	120	140	157	146
	96	113	135	151	129
4%	67	102	112	140	140
	56	98	115	136	137
	49	95	118	133	132

Figure 3 data. Nitrate leaching in bentonite amended sandy soil using N sources during five leaching events' Three replications' data

For $Ca(NO_3)_2$ treatment					
Bentonite application	1d	2 d	4 d	6 d	10 d
0%	635	612	572	487	248
	631	597	570	478	232
	626	607	576	478	245
2%	581	586	493	391	233
	587	592	506	398	240
	580	598	498	402	242
4%	530	562	486	345	192
	541	576	476	342	188
	536	568	478	338	195

Figure 4 data: Residual nitrate concentrations found in soil after leaching events

Soil layer	N + 0%	N + 2%	N + 4%	A + 0%	A + 2%	A + 4%	U + 0%	U + 2%	U + 4%
0-25	36	42	42	30	33	41	32	45	51
	31	46	46	26	34	38	35	44	56
	39	39	42	28	37	35	29	42	57
25-50	38	48	58	33	46	46	28	48	61
	36	45	52	30	48	40	27	46	64
	32	44	54	26	42	43	25	52	67

Figure 5 data: Nitrate concentration in 4% bentonite clay mixed sandy soil after discrete incubation with $Ca(NO_3)_2$ (N), NH_4Cl (A) and $CO(NH_2)_2$ (U)

N	A	U	N + 4%	A + 4%	A + 2%
146	154	172	104	109	125
156	148	178	113	112	134
148	150	170	120	116	130
