# Supplementary material for: Bentonite clay with different nitrogen sources can effectively reduce nitrate leaching from sandy soil
Source: PLoS One. 2022 Dec 22;17(12):e0278824. doi: 10.1371/journal.pone.0278824 (PMC9779043; doi:10.1371/journal.pone.0278824)
Supplement: Attachment — Submitted filename: PONE-D-22-14894_R1-.pdf [file pone.0278824.s004.pdf]

PLOS ONE

Bentonite clay with different nitrogen sources reduce nitrate leaching from sandy soil

--Manuscript Draft--

**Manuscript Number:**	PONE-D-22-14894R1
**Article Type:**	Research Article
**Full Title:**	Bentonite clay with different nitrogen sources reduce nitrate leaching from sandy soil
**Short Title:**	Reducing nitrate leaching from sandy soil using bentonite clay and N sources
**Corresponding Author:**	Zahid Hussain<br>COMSATS University Islamabad<br>Abbottabad, PAKISTAN
**Keywords:**	bentonite; nitrate leaching; N sources; sandy soil
**Abstract:**	Nitrate (NO_3^{-1}) leaching from soils results in the lower soil fertility, reduced crop productivity and increased water pollution. The effects of bentonite clay on NO_3^{-1} leaching from sandy soils are not extensively studied. Therefore, the present lysimetric study determined NO_3^{-1} leaching from bentonite [0, 2 and 4% (m/m)] treated sandy soil under three Nitrogen (N) sources (calcium nitrate [$Ca(NO_3)_2$], ammonium chloride [NH_4Cl], and urea [$CO(NH_2)_2$] at the rate of 300 kg N ha^{-1}). Results showed that bentonite markedly reduced NO_3^{-1} release in the leachate, while 4% bentonite retained higher NO_3 in the soil. The NO_3^{-1} leaching from sandy soil varied with N sources as $Ca(NO_3)_2$ > NH_4Cl > $(CO(NH_2)_2$. At early stages of leaching, higher concentrations of NO_3^{-1} were detected in leachate with both NH_4Cl and $Ca(NO_3)_2$, but leaching of NO_3^{-1} increased with urea at later leaching stages. The amount of total NO_3^{-1} retained in soil was conversely related to the amount of NO_3^{-1} in the leachate. This study indicated that soil amendment with bentonite could efficiently mitigate NO_3^{-1} leaching from sandy soil and hence prevent N fertilizer losses and groundwater pollution.
**Order of Authors:**	Zahid Hussain
	Tang Cheng
	Muhammad Irshad
	Riaz Ahmed Khattak
	Chen Yao
	Di Song
	Muhammad Mohiuddin
**Response to Reviewers:**	A detailed "Response to Reviewers" file has been uploaded.<br><br>Two reviewers (1 and 2) suggested changes in Title of the manuscript. The title was modified keeping in view of both reviewers. However, it can be further modified if suggested<br><br>New references has been added as suggested by all reviewers
**Additional Information:**	
**Question**	**Response**
**Financial Disclosure**<br><br>Enter a financial disclosure statement that describes the sources of funding for the work included in this submission. Review the submission guidelines for detailed requirements. View published research	The authors received no specific funding for this work

Powered by Editorial Manager® and ProduXion Manager® from Aries Systems Corporation

articles from *PLOS ONE* for specific examples.<br><br>This statement is required for submission and **will appear in the published article** if the submission is accepted. Please make sure it is accurate.<br><br>**Unfunded studies**<br>Enter: *The author(s) received no specific funding for this work.*<br><br>**Funded studies**<br>Enter a statement with the following details:<br>• Initials of the authors who received each award<br>• Grant numbers awarded to each author<br>• The full name of each funder<br>• URL of each funder website<br>• Did the sponsors or funders play any role in the study design, data collection and analysis, decision to publish, or preparation of the manuscript?<br>• **NO** - Include this sentence at the end of your statement: *The funders had no role in study design, data collection and analysis, decision to publish, or preparation of the manuscript.*<br>• **YES** - Specify the role(s) played.<br><br>* typeset	
**Competing Interests**<br><br>Use the instructions below to enter a competing interest statement for this submission. On behalf of all authors, disclose any competing interests that could be perceived to bias this work—acknowledging all financial support and any other relevant financial or non-financial competing interests.<br><br>This statement is required for submission and **will appear in the published article** if the submission is accepted. Please make sure it is accurate and that any funding sources listed in your Funding Information later in the submission form are also declared in your Financial Disclosure statement.	The authors have declared that no competing interests exist

Powered by Editorial Manager® and ProduXion Manager® from Aries Systems Corporation

View published research articles from *PLOS ONE* for specific examples.<br><br>**NO authors have competing interests**<br><br>Enter: *The authors have declared that no competing interests exist.*<br><br>**Authors with competing interests**<br><br>Enter competing interest details beginning with this statement:<br><br>*I have read the journal's policy and the authors of this manuscript have the following competing interests: [insert competing interests here]*<br><br>* typeset	
**Ethics Statement**<br><br>Enter an ethics statement for this submission. This statement is required if the study involved:<br><br>• Human participants<br>• Human specimens or tissue<br>• Vertebrate animals or cephalopods<br>• Vertebrate embryos or tissues<br>• Field research<br><br>Write "N/A" if the submission does not require an ethics statement.<br><br>General guidance is provided below. Consult the submission guidelines for detailed instructions. **Make sure that all information entered here is included in the Methods section of the manuscript.**	N/A

**Format for specific study types**<br><br>**Human Subject Research (involving human participants and/or tissue)**<br>• Give the name of the institutional review board or ethics committee that approved the study<br>• Include the approval number and/or a statement indicating approval of this research<br>• Indicate the form of consent obtained (written/oral) or the reason that consent was not obtained (e.g. the data were analyzed anonymously)<br><br>**Animal Research (involving vertebrate animals, embryos or tissues)**<br>• Provide the name of the Institutional Animal Care and Use Committee (IACUC) or other relevant ethics board that reviewed the study protocol, and indicate whether they approved this research or granted a formal waiver of ethical approval<br>• Include an approval number if one was obtained<br>• If the study involved *non-human primates*, add *additional details* about animal welfare and steps taken to ameliorate suffering<br>• If anesthesia, euthanasia, or any kind of animal sacrifice is part of the study, include briefly which substances and/or methods were applied<br><br>**Field Research**<br><br>Include the following details if this study involves the collection of plant, animal, or other materials from a natural setting:<br>• Field permit number<br>• Name of the institution or relevant body that granted permission	
**Data Availability**<br><br>Authors are required to make all data underlying the findings described fully available, without restriction, and from the time of publication. PLOS allows rare exceptions to address legal and ethical concerns. See the PLOS Data Policy and FAQ for detailed information.	No - some restrictions will apply

A Data Availability Statement describing where the data can be found is required at submission. Your answers to this question constitute the Data Availability Statement and **will be published in the article**, if accepted.<br><br>**Important:** Stating 'data available on request from the author' is not sufficient. If your data are only available upon request, select 'No' for the first question and explain your exceptional situation in the text box.<br><br>Do the authors confirm that all data underlying the findings described in their manuscript are fully available without restriction?	
**Describe where the data may be found in full sentences. If you are copying our sample text, replace any instances of XXX with the appropriate details.**<br><br>• If the data are **held or will be held in a public repository**, include URLs, accession numbers or DOIs. If this information will only be available after acceptance, indicate this by ticking the box below. For example: *All XXX files are available from the XXX database (accession number(s) XXX, XXX.).*<br>• If the data are all contained **within the manuscript and/or Supporting Information files**, enter the following: *All relevant data are within the manuscript and its Supporting Information files.*<br>• If neither of these applies but you are able to provide **details of access elsewhere**, with or without limitations, please do so. For example:<br><br>*Data cannot be shared publicly because of [XXX]. Data are available from the XXX Institutional Data Access / Ethics Committee (contact via XXX) for researchers who meet the criteria for access to confidential data.*<br><br>*The data underlying the results presented in the study are available from (include the name of the third party*	All relevant data are within the manuscripts and its supporting information files

Powered by Editorial Manager® and ProduXion Manager® from Aries Systems Corporation

and contact information or URL).<br>• This text is appropriate if the data are owned by a third party and authors do not have permission to share the data.<br><br>* typeset	
Additional data availability information:	

Powered by Editorial Manager® and ProduXion Manager® from Aries Systems Corporation

Manuscript Click here to access/download;Manuscript;Manuscript.docx

Bentonite clay with different nitrogen sources reduce nitrate leaching from sandy soil

Zahid Hussain[a,b,*], Tang Cheng[a,*], Muhammad Irshad[c,], Riaz A. Khattak[d], Chen Yao[e], Di Song[a], Muhammad Mohiuddin[f,]

[a]Yunnan key Laboratory of Pollution Process and Management of Plateau-Lake -Watershed, Yunnan Research Academy of Eco-Environmental Sciences, Kunming, 650034, China

[b]Department of Development Studies, COMSATS University Islamabad (CUI), Abbottabad Campus, Abbottabad 22060, Pakistan

[c]Department of Environmental Sciences, CUI, Abbottabad Campus, Abbottabad 22060, Pakistan

[d]The Brains Institute, Peshawar, Pakistan

[e]Yunnan Infrastructure Investment Co. Ltd., 650599, China

[f]Department of Environmental Sciences, Kohsar University, Murree, Pakistan

[*]Corresponding Authors:

1. Dr. Zahid Hussain (Department of Development Studies, COMSATS University Islamabad, Abbottabad Campus, Abbottabad, Pakistan) (Present Address), (Email: drzahid@cuiatd.edu.pk)

2. Tang Cheng (Yunnan key Laboratory of Pollution Process and Management of Plateau-Lake - Watershed, Yunnan Research Academy of Eco-Environmental Sciences, Kunming, 650034, China, Email: 1290992119@qq.com)

Highlights

- Sandy soils are more prone to nutrient leaching
- Application of N sources enhanced NO_3^{-1} leaching from the sandy soil
- NO_3^{-1} leaching reduces soil fertility and causes groundwater pollution
- Bentonite amendment significantly reduced NO_3^{-1} leaching from sandy soil
- The NO_3^{-1} leaching decreased with N sources in the order as $Ca(NO_3)_2$>NH_4Cl> $CO(NH_2)_2$

Abstract

Nitrate (NO_3^{-1}) leaching from soils results in the lower soil fertility, reduced crop productivity and increased water pollution. The effects of bentonite clay on NO_3^{-1} leaching from sandy soils are not extensively studied. Therefore, the present lysimetric study determined NO_3^{-1} leaching from bentonite [0, 2 and 4% (m/m)] treated sandy soil under three Nitrogen (N) sources (calcium nitrate [$Ca(NO_3)_2$], ammonium chloride [NH_4Cl], and urea [$CO(NH_2)_2$] at the rate of 300 kg N ha^{-1}). Results showed that bentonite markedly reduced NO_3^{-1} release in the leachate, while 4% bentonite retained higher NO_3 in the soil. The NO_3^{-1} leaching from sandy soil varied with N sources as $Ca(NO_3)_2$ > NH_4Cl > $(CO(NH_2)_2$. At early stages of leaching, higher concentrations of NO_3^{-1} were detected in leachate with both NH_4Cl and $Ca(NO_3)_2$, but leaching of NO_3^{-1} increased with urea at later leaching stages. The amount of total NO_3^{-1} retained in soil was conversely related to the amount of NO_3^{-1} in the leachate. This study indicated that soil amendment with bentonite could efficiently mitigate NO_3^{-1} leaching from sandy soil and hence prevent N fertilizer losses and groundwater pollution.

Key words: bentonite; nitrate leaching; N sources; sandy soil

1. Introduction

Growing populations and changing diets require an increase in agricultural production which may lead to an increase in the use of fertilizers. As such, food demand and fertilizer use have been forecasted to double or triple by 2050 [1]. Chemical N fertilizers and organic manures are often applied to the soil in higher amounts for higher agriculture production, which may lead to the N losses due to removal from the cropped fields into the water bodies [2] and/or emission into the atmosphere [3, 4]. Of the applied N for crops, only 40-50% is being incorporated into the

agricultural products [5] and the remainder N is subjected to the substantial losses [6]. The rainfall intensity and irrigation water influence the NO_3^{-1} loss in the soil profile [7, 8]. The N fertilizers, used either as urea or ammonium form, is biochemically converted to NO_3^{-1} which is susceptible to leaching from soil-plant system and enter groundwater bodies [2]. Therefore, an effective technology is required to prevent NO_3^{-1} losses from sandy soils.

Soil NO_3^{-1} is originated from both organic and inorganic N sources. Leaching and drainage studies found that NO_3^{-1} is the major form of N occurred in the soil water [8. 9, 10]. A number of factors including plant characteristics, seasonal and climatic changes, and soil properties govern NO_3 leaching from soils [11, 12]. The specific factors include soil texture, soil N concentration, amount of applied N, type of fertilizer, precipitation amount and intensity, soil water holding capacity, types of crops, root length and N demand of next crop [13, 14]. Leaching of NO_3^{-1}-N is more common than leaching of NH_4-N since both NO_3^{-1}-N and soil are negatively charged [15]. Over application or un-timely application of animal manures or commercial N fertilizers result in the nutrient imbalance in soils which lead to the increased N leaching rates, especially of NO_3^{-1}, into groundwater [12, 16]. Sandy soils, due to low water holding capacity [17], allow NO_3^{-1} to leach down into the groundwater faster than the soils having fine textures, such as clay loams [13, 15]. Thus, leaching of NO_3^{-1} through soil profile can potentially contaminate surface and groundwater [18]. Sandy soils with low organic matter may facilitate leaching of 10-15 mg L^{-1} of NO_3^{-1} to groundwater [19]. About 20-25% of this NO_3^{-1} may enter surface water via buffer streams and wetlands causing eutrophication of water bodies [20, 21].

The increasing unsustainable agricultural use of N fertilizers results in NO_3 leaching into ground waters [22, 23] and runoff into surface water ecosystem producing unfavorable consequences [24], which adversely affect water quality [22, 25]. The increasing potential of

contamination of water resources is linked with the inefficient management of N fertilizer when compared with the natural systems [26, 27, 28]. The concentration of NO_3^{-1} above 10 mg L^{-1} in drinking water are considered as harmful for human health [29]. Higher NO_3^{-1} consumption has been affiliated with various illnesses, e.g., methemoglobinemia has been proven due to ingestion of over nitrate concentrations in water [30, 31]. The endogenous NO_3^{-1} may chemically be transformed to carcinogenic N compounds leading to adverse effects of colorectal cancer [32] and bladder cancer [33].Therefore, developing an effective technology to retain nutrients in soils is imperative to prevent NO_3^{-1} leaching from soils. Soil amendments have been considered as management practice to reduce NO_3^{-1} losses from sandy soils [34]. Bentonite, an alumina-siliceous clay material, has not been previously utilized to control NO_3^{-1} leaching.

Bentonite, like other clays, are hydrous aluminosilicates with fine colloids of < 2 mm of soils [35]. Clays are composed of fine-grained clays minerals and crystals such as quartz, carbonates and oxides [35] and are considered to retain contaminants by anion and cation exchange processes and prevent leaching into groundwaters. Due to effective adsorption capacities, bentonite clay has been used for multiple purposes. Bentonite is also used to remove dyes, radioactive waste, purification of viral RNA and wastewater [36, 37, 38]. Bentonite application as amendment enhanced soil fertility by increased soil carbon and potassium [39], while improved water holding capacity of sandy soils under drought stress [40]. Bentonite application to sandy acidic soil improved soil fertility by increasing availability of macro-nutrient (up to 30%) to plants [41]. Fertilizers, if used in combination with nano-dimensional adsorbents increase nutrient use efficiency and reduce nutrient leaching into groundwaters [42], Clay amended sandy soil significantly reduced N and P leaching by 20% to 60% [43]. Leaching of NH_4-N was reduced by 70% from a mixture of biochar, urea and bentonite plus sepiolite clay [44]. However, it is still

unclear that how the type of fertilizer and application of bentonite clay to soils can mitigate NO_3^{-1} leaching.

A reduction in the NO_3^{-1} leaching was expected when clay material was applied to the soil. Reports evaluating the interactive effects of bentonite material and N sources on the reduction of NO_3^{-1} leaching from sandy soils are scanty. Therefore, the objective of the present study was to investigate the influence of bentonite on NO_3^{-1} leachability from a sandy soil after application of three N sources.

2. Materials and methods

2.1 Lysimeter experiment

A leaching experiment was conducted in the Soil Science Laboratory at COMSATS University Islamabad, Abbottabad Campus, Pakistan following the idea of Zhao et al. [45]. For this purpose, PVC columns, with 0.60 m length and 0.15 m diameter, were installed to run the experiment. The bottles used for collecting leachate were installed on the floor. Filter papers were placed on porous bottom of the columns to prevent soil leaching. The columns were connected with bottles using small pipes for the collection of leachate. The connecting pipes were kept airtight to prevent evaporation from the leachate bottles. Locally collected sandy soil samples from agricultural land (0-12 cm depth) were utilized for the experiment. Bentonite material was commercially purchased and then air-dried. Both the soil and bentonite were analyzed for physico-chemical properties before the experiment. After air drying, 46 kg of the soil was added to each column (0.50 m length). The air-dried clay was applied at the rate of 0, 2 and 4% to the sandy soil packed in a PVC column. Three treatments of nitrogen (N) fertilizers namely calcium nitrate [$Ca(NO_3)_2$], ammonium chloride [NH_4Cl], and urea [$CO(NH_2)_2$] were applied to the soil. Based on the bulk density of soil (1.3 g cm^{-1}), each fertilizer was applied at 300 kg N ha^{-1}. Initially, as

the soil was dry, the amount of first water application was kept higher so that enough water may drain out to collect the leachate. For later leaching events, the leaching fraction (LF) was calculated by dividing the drained water by applied water. Then, the tap water was applied at leaching fraction of 0.3~0.4. Leachate was collected within 24 h after each water application. A total of five leaching events were covered, that is, 1st, 2nd, 4th, 6th and 10th day. The graphical display of the experiment is illustrated in [Figure 1].

Figure 1 Graphical display of lysimeter experiment

2.2 Laboratory analysis

Chemical analyses of soil, bentonite and tap water were carried out before experiment. Soil samples were air-dried and sieved via 2 mm sieve. The soil and air-dried bentonite material were tested for pH and electrical conductivity (EC) in 1:5 (w/v) soil-water suspensions by a pH meter (Model: HANNA HI 8520) and EC meter (Model: 4320 JENWAY), respectively [46]. The soil was saturated for overnight, weighed and then the water holding capacity (WHC) of the soil was calculated by the difference in the weight of soil [47]. The bulk density of soil was determined using cylindrical cores. The soil sample was weighed and placed in the oven at 105℃ for 8 to 12 hours until the weight was constant. Bulk density was then calculated in the same way as described by Grossman and Reinsch [48].

The post-experiment soil was sampled in two layers (0-25 and 25-50 cm) from the PVC columns and was thoroughly mixed. A 10 g soil sample was shaken in 100 mL distilled water for 1 h and then the suspension was filtered. Moisture content in the soil samples was adjusted by oven drying few grams of soil. The leachate collected from 5 events was analyzed for NO_3 concentration. The NO_3^{-1} concentration in pre- and post-experiment soil, bentonite and leachate

was determined by UV spectrophotometer (Model: LI-UV-7000) at 220 nm [49]. All the reagents/chemicals of Sigma Aldrich, Germany, were utilized during the experiment.

2.3 Statistical analysis

Data were statistically analyzed by OriginLab 2021 for graphical presentation. The three-way analysis of variance (ANOVA) was performed on Sigmaplot. The three factors were taken as bentonite (0, 2% and 4%), N fertilizer sources ([$Ca(NO_3)_2$], [NH_4Cl] and [$CO(NH_2)_2$]), and leaching events (1st, 2nd, 4th, 6th and 10th day) with three replications. A post hoc Tukey test was also performed to determine the significant difference between the levels of factors.

3. Results

3.1. Pre-experiment chemical analysis

Bentonite clay, soil and water were analyzed for chemical properties before experiment, which are presented in Table 1. Analysis revealed that soil had highest NO_3^{-1} concentrations, compared to bentonite clay. The tap water had low concentrations of NO_3^{-1}. The electrical conductivity (EC) of bentonite was highest compared to soil and water samples, but still it fell below the category of non-saline (EC<4 dS m^{-1}). However, the pH of bentonite was lower (pH<7) making it more acidic as compared to soil and water.

Table 1 Nitrate, electrical conductivity (EC) and pH of bentonite clay material, soil and tap water

Material	Nitrate	EC (mS m^{-1})	pH
Bentonite	23.5 mg kg^{-1}	126.7	5.7
Soil	34.4 mg kg^{-1}	87.8	7.8
Tap water	7.8 mg L^{-1}	37.5	7.2

3.2. Effect of bentonite and N sources on NO_3^{-1} leaching

Impact of bentonite clay mixed with different N sources on NO_3^{-1} leaching is illustrated in [Figure *2*]. Results showed NO_3^{-1} concentration decreased by 12 to 19% in the leachate, irrespective of the source of N with increasing bentonite rates. The significantly highest reduction (20-25%) in NO_3^{-1} leaching was recorded with $Ca(NO_3)_2$ with 4% bentonite as compared with $CO(NH_2)_2$ and NH_4Cl at similar bentonite rates. At early stages of leaching, the leachate showed higher concentrations of NO_3^{-1} in leachate with both NH_4Cl and $Ca(NO_3)_2$, but leaching of NO_3 increased with urea sources at later leaching stages [Figure 3]. Total NO_3^{-1} loads were higher in soil with urea and $Ca(NO_3)_2$ treated soil at 4% bentonite as compared to NH_4Cl [Figure 4]. The incubation of soil with bentonite (4%) reduced NO_3 content by 7%, 20% and 8% with $Ca(NO_3)_2$, NH_4Cl and $CO(NH_2)_2$ treated soil, respectively [Figure *5*].

Figure 2 Average amount of leachate nitrate from bentonite treated sandy soil (from day 1 to day 10). N, A and U indicate $Ca(NO_3)_2$, NH_4Cl and $CO(NH_2)_2$, respectively. Bentonite clay was applied at the rate of 0%, 2% and 4%. *, ** and *** show significant differences at $P<0.05$, $P<0,01$ and $P<0.001$, respectively

Figure 3 Nitrate leaching in bentonite amended sandy soil using $Ca(NO_3)_2$ (a), NH_4Cl (b), and $CO(NH_2)_2$ (c) with 0%, 2% and 4% bentonite during five leaching events (days). *, ** and *** show significant difference at $P<0.05$, $P<0,01$ and $P<0.001$, respectively

Figure 4 Residual nitrate loads (g) in soil after leaching events (days) at 0%, 2% and 4% bentonite with $Ca(NO_3)_2$ (N), NH_4Cl (A), and $CO(NH_2)_2$ (U). *, ** and *** show significant difference at $P<0.05$, $P<0,01$ and $P<0.001$, respectively

Figure 5 Nitrate concentration in 4% bentonite clay mixed sandy soil after discrete incubation with $Ca(NO_3)_2$ (N), NH_4Cl (A) and $CO(NH_2)_2$ (U), *, ** and *** show significant difference between treatments at $P<0.05$, $P<0,01$ and $P<0.001$, respectively

3.3. Statistical Analysis

A three-way analysis of variance (ANOVA) was performed on NO_3^{-1} leaching with 3 N sources, 3 levels of bentonite and 5 leaching events [Table 2]. Analysis showed that there was significant difference (P<0.001) within N sources, bentonite levels and leaching events (days). There was statistically significant (P<0.01) interaction among all the factors. The Tukey test revealed significant (P<0.05) difference between 0% and 2%, and 0% and 4% bentonite with all N sources on NO_3^{-1} leaching, but the difference was not significant between 2% and 4% bentonite with all N sources [Table 2].

Table 2 Summary ANOVA on effect of bentonite and N sources on NO_3^{-1} leaching at different leaching events

Source of Variation	DF	SS	MS	F	P
N sources (N)	2	2943336.13	1471668	14453	< 0.01
Bentonite levels (B)	2	74157.91	37078	364	< 0.01
Leaching event / day (D)	4	282061.82	70515	692	< 0.01
N x B	4	11270.48	2817	27	< 0.01
N x D	8	589355.42	73669	723	< 0.01
B x D	8	15955	1994	19	< 0.01
N x B x D	16	24125	1507	14	< 0.01
Residual	90	9164	101		
Total	134	3949426	29473		

4. Discussion

Results showed that irrespective of the source of N, the NO_3^{-1} leaching consistently decreased with increasing bentonite application showing the sequences of fertilizer type as: $Ca(NO_3)_2 > NH_4Cl > CO(NH_2)_2$ [Figure *2*]. Form of N leached from the soil columns was closely related to the type of fertilizer applied to the soil [50]. The NO_3^{-1} fertilizers appeared to be more sensitive to the leaching, especially in sandy soils [51] and also to the denitrification [52] as compared to urea or ammonium fertilizers. Also, NO_3^{-1} leaching is substantially higher in free-drained soils [53], such as sandy soil with macropores used in the present study.

Application of bentonite decreased NO_3^{-1} leaching, regardless to the rate of application. The [NO_3^{-1}] decreased with increasing bentonite treatments level. Whereby higher NO_3^{-1} concentration was observed in NO_3^{-1} and NH_4 containing fertilizers during the initial leaching [Figure 3]. Urea form of N showed consistent increases in NO_3^{-1} concentrations in water collected in later leaching stages [Figure 3]. This could be associated with increased nitrification process in soils under unsaturated conditions [54], which might have resulted in increased NO_3^{-1} leaching at later stages. Accumulation of NO_3^{-1} was more in the soil sampled from the lower layer of the column after a leaching process, showing the sequence as $CO(NH_2)_2 > NH_4Cl > Ca(NO_3)_2$.

The amount of total NO_3^{-1} retained in soil was termed as nitrate loads conversely related to the amount of NO_3^{-1} in the leachates [Figure 4]. An enhanced application of bentonite significantly retained NO_3^{-1} in the soil columns. A higher amount of NO_3^{-1} was retained in the soil amended with 4% bentonite. The application of bentonite clay enhanced soil moisture and improved macro-aggregate development [55] which improved soil quality through structural development, by increased exchange of anions and cations [56] and helped in reduced leaching while promoting nutrient retention [57].

Enrichment of sandy soils with bentonite increased the porosity and altered the pore-size distribution [58]. The interactions of bentonite with biochar and urea improved soil properties by diffusing soil moisture which controlled the mobility of nutrients within soils [59], thus with high water retaining capacity, increased exchange capacity, swelling, thermal stability and slow-releasing characteristics, bentonite offers valuable solution to reduced nutrient leaching from loose soils.

Higher quantity of NO_3^{-1} was retained in urea treated soil followed by nitrate and ammonium containing fertilizers [Figure 4]. This could be attributed to the transformation of N[illegible]$^{-1}$ in urea contained soil after few days of incubation. Across all N sources, the application of bentonite (4%) markedly limited the release of NO_3^{-1}. After incubation, bentonite contents reduced the magnitude of NO_3^{-1} among fertilizers as follow: 7% in $Ca(NO_3)_2$, 20% in NH_4Cl and 8% in urea treated soil [Figure 5]. The soil having negatively charged sites attracts more positively charged NH_4 as compared to the negatively charged NO_3^{-1}, and therefore the NH_4 has been considered to be a less mobile in soils, than NO_3^{-1} [60, 61].

The effect of bentonite on the leachability of NO_3^{-1} varied with different N fertilizers. The NO_3^{-1} leaching consistently decreased with increasing bentonite application showing the sequences of fertilizer type as: $Ca(NO_3)_2$ > NH_4Cl > $CO(NH_2)_2$ [Figure 2]. Addition of Ca as $Ca(NO_3)_2$ increased the adsorptive capacity of bentonite at low pH (5-6), while higher concentration of NO_3^{-1} ion due to the addition of calcium nitrate as N source resulted in increased adsorption of NO_3^{-1} at bentonite clay surfaces [62]. The mechanism can be further explained by Ca hydrolysis, which resulted into the formation of less soluble $Ca(OH)_2$, releasing more H^+ ions, thus acidifying the media [Eq 1][63]. Under low pH, the anion exchange capacity of bentonites in significantly increased which offers more positive sites to attract NO_3^{-1} on its surface [Eq 2], which

increased the retention of NO_3^{-1} ions on soil colloids due to adsorption phenomenon [Eq 3]. This ultimately reduced NO_3^{-1} leaching from sandy soil and showed lower NO_3^{-1} concentrations in leachate. The entire process can be explained as follows, where A is dissociated anion and X is the soil colloidal surface

$$Ca^{2+} + H_2O \rightleftharpoons Ca(OH)_2 + 2H^+ \quad [Eq\ 1]$$

$$X + H^+ \rightleftharpoons X^+ + HA \quad [Eq\ 2]$$

$$X^+ + NO_3^- \rightleftharpoons XNO_3 \quad [Eq\ 3]$$

Surface charge of variable charge clays varies with changes in pH of soil solution, therefore, at low pH, the anion exchange capacity exceeds cation exchange capacities which retain more NO_3^{-1} on its surfaces [64], while increasing the mobility of NH_4 in such conditions. However, the mobility of both N forms can be maintained by adjusting the rates of $Ca(NO_3)_2$ and urea along with bentonite clay under field conditions. The study suggested that bentonite amendment with $Ca(NO_3)_2$ as N sources could effectively mitigate NO_3^{-1} leaching from sandy soils.

5. Conclusions

It is concluded that N sources and bentonite application were important factors affecting the NO_3^{-1} leaching from sandy soil. Application of N sources enhanced NO_3^{-1} leaching from the sandy soil. The NO_3^{-1} leaching decreased in the order of $Ca(NO_3)_2$ > NH_4Cl > urea. Bentonite substantially reduced NO_3^{-1} in the leachate. Urea showed higher NO_3^{-1} at the later leaching events. Higher contents of NO_3^{-1} were retained in the soil with 4% bentonite. Higher NO_3^{-1} contents were accumulated in the lower part of the soil column after a leaching process. This experiment suggests that bentonite clay chemistry with added $Ca(NO_3)_2$ provide better understanding of anion exchange capacity to retain higher NO_3^{-1} concentrations in soil, thereby decreasing NO_3^{-1} leaching from

sandy soil. Further research is suggested to investigate the effects of different clay types on the dynamics of nitrate under field conditions.

Authors Contributions

Z. Hussain., M. Irshad., R.A. Khattak. led the research study and prepared the manuscript. M. Mohiuddin., T. Cheng., C. Yao. analyzed the data and prepared graphs. D. Song. facilitated the research. R.A. Khattak edited the manuscript for technical inputs. All the authors discussed and reviewed the manuscript.

References

1. Elferink M, Schierhorn F (2016) Global demand for food is rising. Can we meet it? Harvard Business Review, Harward Business Publishing, Canada.
2. Bijay-Singh, Craswell E (2021) Fertilizers and nitrate pollution of surface and ground water: an increasing pervasive global problem. SN Applied Science 3: 518. https://doi.org/10.1007/s42452-021-04521-8
3. Shah AN, Iqbal J, Tanveer M et al. (2017) Nitrogen fertilization and conservation tillage: a review on growth, yield, and greenhouse gas emissions in cotton. Environmental Science and Pollution Research 24: 2261–2272. https://doi.org/10.1007/s11356-016-7894-4.
4. Mosongo PS, Pelster DE, Li X, Gaudel G, Wang Y, Chen S, et al. (2022) Greenhouse Gas Emissions Response to Fertilizer Application and Soil Moisture in Dry Agricultural Uplands of Central Kenya. Atmosphere 13: 463. https://doi.org/10.3390/atmos13030463
5. Houlton BZ, Almaraz M, Aneja V, Austin AT, Bai E, Cassman KG, et al. (2019) A world of cobenefits: Solving the global nitrogen challenge. Earth's Future 7: 865–872.
6. Xu P, Chen A, Houlton BZ, Zeng Z, Wei S, Zhao C, et al. (2020) Spatial Variation of Reactive Nitrogen Emissions from China's Croplands Codetermined by Regional

Urbanization and Its Feedback to Global Climate Change. Geophysical Research Letters 47: e2019GL086551.

7. Pathak H, Jain N, Bhatia A, Kumar A, Chatterjee D, et al. (2016) Improved nitrogen management: A key to climate change adaptation and mitigation. Indian Journal of Fertilizers 12: 151–162.

8. Lu J, Bai Z, Velthof GL, Wu Z, Chadwick D, Ma L (2019) Accumulation and leaching of nitrate in soils in wheat-maize production in China. Agricultural Water Management 212: 407-415. https://doi.org/10.1016/j.agwat.2018.08.039

9. Kladivko EJ, Van Scoyoc GE, Monke EJ, Oates KM, Pask W (1991) Pesticides and nutrient movement into subsurface tile drains on s silt loam soil in Indiana. Journal of Environmental Quality 20: 264-270.

10. Jacinthe PA, Dick WA, Brown LC (1999) Bioremediation of nitrate-contaminated shallow soils using water table management techniques: Nitrate removal efficiency. Transactions of the American Society of Agricultural Engineers 42: 1251-1259.

11. Bibi S, Saifullah, Naeem A, Dahlawi S (2016) Environmental Impacts of Nitrogen Use in Agriculture, Nitrate Leaching and Mitigation Strategies. Hakeem et al. (eds.), Soil Science: Agricultural and Environmental Prospectives, Springer International Publishing Switzerland. https://doi.org/10.1007/978-3-319-34451-5_6

12. Tamagno S, Eagle AJ, McLellan E, van Kessel C, Linquist B A, Ladha JK et al. (2022) Quantifying N leaching losses as a function of N balance: A path to sustainable food supply chains. Agriculture, Ecosystems and Environment 324: 107714. https://doi.org/10.1016/j.agee.2021.107714

13. Hallaq AHA (2010) The impact of soil texture on nitrates leaching into groundwater in the North Governorate, Gaza Strip. Journal of Social Sciences 38(2): 1-37.

14. Plošek L, Elbl J, Lošák T, Kužel S, Kintl A, Juřička D et al. (2017) Leaching of mineral nitrogen in the soil influenced by addition of compost and N-mineral fertilizer. Acta Agriculturae Scandinavica, Section B. Soil and Plant Science 67(7): 607-614. https://doi.org/10.1080/09064710.2017.1322632

15. Huddell AM, Galford GL, Tully KL, Crowley C, Palm CA, Neill C et al. (2020) Meta-analysis on the potential for increasing nitrogen losses from intensifying tropical agriculture. Global Change Biology 26: 1668-1680. https://doi.org/10.1111/gcb.14951

16. Hatfield JL, Cambardella CA (2001) Nutrient management in cropping systems. In McFarland J and Sanderson M (ed.) Integrated management of land application of animal waste. (In press.). American Society of Agricultural Engineers, St. Joseph, MI.

17. Rasa K, Heikkinen J, Hannula M, Arstila K, Kulju S, Hyvaluoma J (2018) How and why does willow biochar increase a clay soil water retention capacity? Biomass and Bioenergy 119: 346-353. 10.1016/j.biombioe.2018.10.004

18. Wang H, Gao J-e, Li X-h, Zhang S-l, Wang H-j (2015) Nitrate Accumulation and Leaching in Surface and Ground Water Based on Simulated Rainfall Experiments. PLoS ONE 10(8): e0136274. https://doi.org/10.1371/journal.pone.0136274

19. Gatiboni L, Osmond D (2019) Nitrogen Management and Water Quality, Publication No: AG-439-02, NC State Extension Publications, NC State University, USA.

20. Johnson S, Burchell MR, Evans RO, Osmond DL, Gilliam JW (2012) Riparian Buffer Located in an Upland Landscape Position Does Not Enhance Nitrate-nitrogen Removal. Ecological Engineering 52: 252-261. http://dx.doi.org/10.1016/j.ecoleng.2012.11.006.

21. King SE, Osmond DL, Smith J, Dukes M, Evans RO, Knies S et al. (2015) Effects of Riparian Buffer Vegetation and Width: A 12-year Retrospective Study. *Journal of Environmental Quality* 45(4): 1243-1251. https://doi.org/10.2134/jeq2015.06.0321

22. Hansen B, Thorling L, Schullehner J et al. (2017) Groundwater nitrate response to sustainable nitrogen management. Scientific Reports 7: 8566. https://doi.org/10.1038/s41598-017-07147-2

23. Ayyub CM, Haidar MW, Zulfiqar F, Abideen ZU, Wright SR (2019) Potato tuber yield and quality in response to different nitrogen fertilizer application rates under two split doses in an irrigated sandy loam soil. Journal of Plant Nutrition 42(15): 1850-1860. https://doi.org/10.1080/01904167.2019.1648669

24. Alexander RB, Smith RA, Schwarz GE (1995) The regional transport of point and non-point source of nitrogen to the Gulf of Mexico. In Proc. Gulf of Mexico Hypoxia Conf. Kenner LA, 5-6 Dec 1995. USEPA Publication National Center for Environment. Publ. and Inf. Washington, D.C.

25. Zeb S, Waseem A, Malik AH, Mahmood Q (2011) Water quality assessment of Siran River, Pakistan. International Journal of Physical Sciences 6: 7789 – 7798.

26. Kanwar RS, Stilenberg DER, Pfeiffer DL, Karlen TS, Simpkins WW (1993) Transport of nitrate and pesticides to shallow groundwater systems as affected by tillage and crop rotation practices, 270-273. In Proc. National Conference on Agricultural Research to Protect Water Quality.

27. Randall GW (1997) Nitrate-N in surface waters as influenced by climatic conditions and agricultural practices. In Proc. Agriculture. and Hypoxia in the Mississippi Watershed

Conference, St. Louis, MO. 14–15 July 1997. American Farm Bureau Federation, Park Ridge, IL.

28. Cambardella CA, Moorman TB, Jaynes DB, Parkin TB, Karlen DL (1999) Water quality in Walnut Creek watershed: Nitrate nitrogen in soils, subsurface drainage water and shallow groundwater. Journal of Environmental Quality 28: 25-34.

29. EPA (2022) National Primary Drinking Water Regulations. Available at: https://www.epa.gov/ground-water-and-drinking-water/national-primary-drinking-water-regulations#Inorganic (Date of access: 28.08.2022).

30. Kross BC, Hallberg GR, Bruner R, Cherryholmes K, Johnson KJ (1993) The nitrate contamination of private well water in Iowa. American Journal of Public Health 83: 270-272.

31. WHO (2022) Guidelines for drinking-water quality 4th ed., Available at: https://www.who.int/publications/i/item/9789240045064 (Date of access: 28.08.2022).

32. Espejo-Herrera N et al. (2016) Colorectal cancer risk and nitrate exposure through drinking water and diet. International Journal of Cancer 139: 334–346. https://doil.org/10.1002/ijc.30083.

33. Jones RR et al. (2016) Nitrate from Drinking Water and Diet and Bladder Cancer Among Postmenopausal Women in Iowa. Environmental Health Perspectives 124: 1751–1758. https://doi.org/10.1289/EHP191.

34. Irshad M, Waseem A, Umar M, Sabir MA (2014) Leachability of nitrate from sandy soil using waste amendment. Communications in Soil Science and Plant Analysis 45: 680-687.

35. Sirivastava A, Singh PK (2017) Adsorption of Nitrate from Ground Water using Indian Bentonite: Fixed Bed Column Study. International Journal of Engineering Research and Technology (IJERT) 6(5): 390-394.

36. Bentahar S, Dnik A, Khomri ME, Messaoudi NE, Mohammad AD, Noureddine E et al. (2018) Removal of a cationic dye from aqueous solution by natural clay. Journal of Groundwater for Sustainable Development 6: 255–262. https://doi.org/10.1016/j.gsd.2018.02.002

37. De Castro MLFA, Abad MLB, Sumalinog DAG, Abarca RRM, Paoprasert P, de Luna MDG (2018) Adsorption of Methylene Blue dye and Cu(II) ions on EDTA-modified bentonite: Isotherm, kinetic and thermodynamic studies. J. Sustain. Environ. Res. 28, 197–205. https://doi.org/10.1016/j.serj.2018.04.001

38. Lopez-Fernandez M, Vilchez-Vargas R, Jroundi F, Boon N, Pieper D, Merroun ML (2018) Microbial community changes induced by uranyl nitrate in bentonite clay microcosms. Applied Clay Science 160: 206-216. https://doi.org/10.1016/j.clay.2017.12.034

39. Hall DJM, Jones HR, Crabtree WL, Daniels TL (2010) Claying and deep ripping can increase crop yields and profits on water repellent sands with marginal fertility in southern Western Australia. Australian Journal of Soil Research 48: 178–187. https://doi.org/10.1071/SR09078.

40. Mi J, Gregorich EG, Xu S, McLaughlin NB, Liu J (2020) Effect of bentonite as a soil amendment on field water‐ holding capacity, and millet photosynthesis and grain quality. Scientific Reports 10: 18282. https://doi.org/10.1038/s41598-020-75350-9.

41. Czaban J, Siebielec G (2013) Effects of Bentonite on Sandy Soil Chemistry in a Long-Term Plot Experiment (II); Effect on pH, CEC, and Macro- and Micronutrients. Polish Journal of Environmental Studies 22(6): 1669-1676.

42. Zulfiqar F, Navarro M, Ashraf M, Akram NA, Munné-Bosch S (2019) Nanofertilizer use for sustainable agriculture: Advantages and limitations. Plant Science 289: 110270. https://doi.org/10.1016/j.plantsci.2019.110270.

43. Tahir S, Marschner P (2017) Clay Addition to Sandy Soil Reduces Nutrient Leaching—Effect of Clay Concentration and Ped Size. Communications in Soil Science and Plant Analysis 48(15): 1813–1821. https://doi.org/10.1080/00103624.2017.1395454.

44. Shi W, Ju Y, Bian R, Li L, Joseph S, Mitchell RG et al. (2020) Biochar bound urea boosts plant growth and reduces nitrogen leaching. Science of Total Environment 701: 134424. https://doi.org/10.1016/j.scitotenv.2019.134424

45. Zhao C, Hu C, Huang W, Sun X, Tan Q, Di H (2010) A lysimeter study of nitrate leaching and optimum nitrogen application rates for intensively irrigated vegetable production systems in Central China. Journal of Soils and Sediments 10: 9–17.

46. Richard LA (1954) Diagnosis and improvement of saline and alkali soils (USDA Handbook 60) Washington, DC: USDA.

47. Basso AS, Miguez FE, Laird DA, Horton R, Westgate M (2013) Assessing potential of biochar for increasing water-holding capacity of sandy soils. GCB-Bioenergy 5(2): 132-142. https://doi.org/10.1111/gcbb.12026

48. Grossman RB, Reinsch TG (2002) Bulk density and linear extensibility: core method. In: Dane JH, Topp GC (Eds.), Methods of Soil Analysis. Part 4. Physical Methods. Soil Science Society of America, Inc., Madison, WI, pp. 208–228.

49. Norman RJ, Stucki JW (1981) The determination of nitrate and nitrite in soil extracts by ultraviolet spectrophotometry. Soil Science Society of America 45: 347-353.

50. Zhou JB, Xi JG, Chen ZJ, Li SX (2006) Leaching and transformation of nitrogen fertilizers in soil after application of N with irrigation: A soil column method. Pedosphere 16(2): 245-252.

51. De Boer HC, Deru JGC, Hoekstra NJ, Van Eekeren N (2016) Strategic timing of nitrogen fertilization to increase root biomass and nitrogen-use efficiency of *Lolium perenne L*. Plant and Soil 407: 81-90.

52. Harty MA, Forrestal PJ, Watson CJ, McGeough KL, Carolan R, Elliot C et al. (2016) Reducing nitrous oxide emissions by changing N fertilizer use from calcium ammonium nitrate (CAN) to urea based formulations. Science of Total Environment 563-564: 576-586.

53. De Boer HC (2017) Nitrate leaching from liquid cattle manure compared to synthetic fertilizer applied to grassland or silage maize in the Netherlands. Wageningen Livestock Research Wageningen, October.

54. Zhang YM, Chen DL, Zhang JB, Edis R, Hu CS, Zhu AN (2004) Ammonia volatilization and denitrification losses from an irrigated maize-wheat rotation field in the North China Plain. Pedosphere 14(4): 533-540.

55. Zhang H, Chen W, Zhao B, Phillips LA, Zhou Y, Lapen DR et al. (2020) Sandy soils amended with bentonite induced changes in soil microbiota and fungistasis in maize fields. Applied Soil Ecology 146: 103378. https://doi.org/10.1016/j.apsoil.2019.103378.

56. Churchman GJ, Noble A, Bailey G, Chittleborough D, Harper R (2014) Clay addition and redistribution to enhance carbon sequestration in soils Soil Carbon. In: Hartemink AE,

McSweeney K (eds.) Soil Carbon. Springer International Publishing, Cham, Switzerland, pp. 327-335. https://dx.doi.org/10.1007/978-3-319-04084-4_34 .

57. Satje A, Nelson PN (2009) Bentonite treatments can improve the nutrient and water holding capacity of sugarcane soils in the wet tropics. Proceedings: Australian Society of Sugarcane Technology 31: 166-176.

58. Suzuki S, Noble AD, Ruaysoongnern S, Chinabut N (2007) Improvement in water-holding capacity and structural stability of a sandy soil in northeast Thailand. Arid Land Research Management 21: 37-49.

59. Liu X, Liao J, Song H et al. (2019) A Biochar-Based Route for Environmentally Friendly Controlled Release of Nitrogen: Urea-Loaded Biochar and Bentonite Composite. Scientific Reports 9: 9548. https://doi.org/10.1038/s41598-019-46065-3.

60. Wang FL, Alva AK (2000) Ammonium adsorption and desorption in sandy soils. Soil Science Society of American Journal 64: 1669-1674.

61. Wang Y, Zhang YP (2004) Ammonium adsorption in a Eum-Orthic Anthrosol at different solution / soil ratios and temperature. Pedosphere 14(2): 253-257.

62. Ouakouak A, Youcef L, Achour S, Hamdi N (2018) Kinetics and equilibrium studies of nitrates adsorption onto natural Bentonite. Conference paper, Colloque International Sciences & Techniques de l'Eau & Environnement (STEE).

63. Lohinger H (2019) Calcium Hydroxide. Epina ebook Series. UC Davis, University of California, USA

64. Sollins P, Robertson GP, Uehara G (1988) Nutrient Mobility in Variable- and Permanent-Charge Soils. Biogeochemistry 6(3): 181-199.

Supplementary Files

Supplementary File
Bentonite experimer

Manuscript (clean copy) Click here to access/download;Manuscript;Manuscript.docx

Bentonite clay with different nitrogen sources reduce nitrate leaching from sandy soil

Zahid Hussain[a,b,*], Tang Cheng[a,*], Muhammad Irshad[c,], Riaz A. Khattak[d], Chen Yao[e], Di Song[a], Muhammad Mohiuddin[f,]

[a]Yunnan key Laboratory of Pollution Process and Management of Plateau-Lake -Watershed, Yunnan Research Academy of Eco-Environmental Sciences, Kunming, 650034, China

[b]Department of Development Studies, COMSATS University Islamabad (CUI), Abbottabad Campus, Abbottabad 22060, Pakistan

[c]Department of Environmental Sciences, CUI, Abbottabad Campus, Abbottabad 22060, Pakistan

[d]The Brains Institute, Peshawar, Pakistan

[e]Yunnan Infrastructure Investment Co. Ltd., 650599, China

[f]Department of Environmental Sciences, Kohsar University, Murree, Pakistan

[*]Corresponding Authors:

1. Dr. Zahid Hussain (Department of Development Studies, COMSATS University Islamabad, Abbottabad Campus, Abbottabad, Pakistan) (Present Address), (Email: drzahid@cuiatd.edu.pk)

2. Tang Cheng (Yunnan key Laboratory of Pollution Process and Management of Plateau-Lake - Watershed, Yunnan Research Academy of Eco-Environmental Sciences, Kunming, 650034, China, Email: 1290992119@qq.com)

Highlights

- Sandy soils are more prone to nutrient leaching
- Application of N sources enhanced NO_3^{-1} leaching from the sandy soil
- NO_3^{-1} leaching reduces soil fertility and causes groundwater pollution
- Bentonite amendment significantly reduced NO_3^{-1} leaching from sandy soil
- The NO_3^{-1} leaching decreased with N sources in the order as $Ca(NO_3)_2$>NH_4Cl> $CO(NH_2)_2$

Abstract

Nitrate (NO_3^{-1}) leaching from soils results in the lower soil fertility, reduced crop productivity and increased water pollution. The effects of bentonite clay on NO_3^{-1} leaching from sandy soils are not extensively studied. Therefore, the present lysimetric study determined NO_3^{-1} leaching from bentonite [0, 2 and 4% (m/m)] treated sandy soil under three Nitrogen (N) sources (calcium nitrate [$Ca(NO_3)_2$], ammonium chloride [NH_4Cl], and urea [$CO(NH_2)_2$] at the rate of 300 kg N ha^{-1}). Results showed that bentonite markedly reduced NO_3^{-1} release in the leachate, while 4% bentonite retained higher NO_3 in the soil. The NO_3^{-1} leaching from sandy soil varied with N sources as $Ca(NO_3)_2 > NH_4Cl > (CO(NH_2)_2$. At early stages of leaching, higher concentrations of NO_3^{-1} were detected in leachate with both NH_4Cl and $Ca(NO_3)_2$, but leaching of NO_3^{-1} increased with urea at later leaching stages. The amount of total NO_3^{-1} retained in soil was conversely related to the amount of NO_3^{-1} in the leachate. This study indicated that soil amendment with bentonite could efficiently mitigate NO_3^{-1} leaching from sandy soil and hence prevent N fertilizer losses and groundwater pollution.

Key words: bentonite; nitrate leaching; N sources; sandy soil

1. Introduction

Growing populations and changing diets require an increase in agricultural production which may lead to an increase in the use of fertilizers. As such, food demand and fertilizer use have been forecasted to double or triple by 2050 [1]. Chemical N fertilizers and organic manures are often applied to the soil in higher amounts for higher agriculture production, which may lead to the N losses due to removal from the cropped fields into the water bodies [2] and/or emission into the atmosphere [3, 4]. Of the applied N for crops, only 40-50% is being incorporated into the

agricultural products [5] and the remainder N is subjected to the substantial losses [6]. The rainfall intensity and irrigation water influence the NO_3^{-1} loss in the soil profile [7, 8]. The N fertilizers, used either as urea or ammonium form, is biochemically converted to NO_3^{-1} which is susceptible to leaching from soil-plant system and enter groundwater bodies [2]. Therefore, an effective technology is required to prevent NO_3^{-1} losses from sandy soils.

Soil NO_3^{-1} is originated from both organic and inorganic N sources. Leaching and drainage studies found that NO_3^{-1} is the major form of N occurred in the soil water [8. 9, 10]. A number of factors including plant characteristics, seasonal and climatic changes, and soil properties govern NO_3 leaching from soils [11, 12]. The specific factors include soil texture, soil N concentration, amount of applied N, type of fertilizer, precipitation amount and intensity, soil water holding capacity, types of crops, root length and N demand of next crop [13, 14]. Leaching of NO_3^{-1}-N is more common than leaching of NH_4-N since both NO_3^{-1}-N and soil are negatively charged [15]. Over application or un-timely application of animal manures or commercial N fertilizers result in the nutrient imbalance in soils which lead to the increased N leaching rates, especially of NO_3^{-1}, into groundwater [12, 16]. Sandy soils, due to low water holding capacity [17], allow NO_3^{-1} to leach down into the groundwater faster than the soils having fine textures, such as clay loams [13, 15]. Thus, leaching of NO_3^{-1} through soil profile can potentially contaminate surface and groundwater [18]. Sandy soils with low organic matter may facilitate leaching of 10-15 mg L^{-1} of NO_3^{-1} to groundwater [19]. About 20-25% of this NO_3^{-1} may enter surface water via buffer streams and wetlands causing eutrophication of water bodies [20, 21].

The increasing unsustainable agricultural use of N fertilizers results in NO_3 leaching into ground waters [22, 23] and runoff into surface water ecosystem producing unfavorable consequences [24], which adversely affect water quality [22, 25]. The increasing potential of

contamination of water resources is linked with the inefficient management of N fertilizer when compared with the natural systems [26, 27, 28]. The concentration of NO_3^{-1} above 10 mg L^{-1} in drinking water are considered as harmful for human health [29]. Higher NO_3^{-1} consumption has been affiliated with various illnesses, e.g., methemoglobinemia has been proven due to ingestion of over nitrate concentrations in water [30, 31]. The endogenous NO_3^{-1} may chemically be transformed to carcinogenic N compounds leading to adverse effects of colorectal cancer [32] and bladder cancer [33].Therefore, developing an effective technology to retain nutrients in soils is imperative to prevent NO_3^{-1} leaching from soils. Soil amendments have been considered as management practice to reduce NO_3^{-1} losses from sandy soils [34]. Bentonite, an alumina-siliceous clay material, has not been previously utilized to control NO_3^{-1} leaching.

Bentonite, like other clays, are hydrous aluminosilicates with fine colloids of < 2 mm of soils [35]. Clays are composed of fine-grained clays minerals and crystals such as quartz, carbonates and oxides [35] and are considered to retain contaminants by anion and cation exchange processes and prevent leaching into groundwaters. Due to effective adsorption capacities, bentonite clay has been used for multiple purposes. Bentonite is also used to remove dyes, radioactive waste, purification of viral RNA and wastewater [36, 37, 38]. Bentonite application as amendment enhanced soil fertility by increased soil carbon and potassium [39], while improved water holding capacity of sandy soils under drought stress [40]. Bentonite application to sandy acidic soil improved soil fertility by increasing availability of macro-nutrient (up to 30%) to plants [41]. Fertilizers, if used in combination with nano-dimensional adsorbents increase nutrient use efficiency and reduce nutrient leaching into groundwaters [42], Clay amended sandy soil significantly reduced N and P leaching by 20% to 60% [43]. Leaching of NH_4-N was reduced by 70% from a mixture of biochar, urea and bentonite plus sepiolite clay [44]. However, it is still

unclear that how the type of fertilizer and application of bentonite clay to soils can mitigate NO_3^{-1} leaching.

A reduction in the NO_3^{-1} leaching was expected when clay material was applied to the soil. Reports evaluating the interactive effects of bentonite material and N sources on the reduction of NO_3^{-1} leaching from sandy soils are scanty. Therefore, the objective of the present study was to investigate the influence of bentonite on NO_3^{-1} leachability from a sandy soil after application of three N sources.

2. Materials and methods

2.1 Lysimeter experiment

A leaching experiment was conducted in the Soil Science Laboratory at COMSATS University Islamabad, Abbottabad Campus, Pakistan following the idea of Zhao et al. [45]. For this purpose, PVC columns, with 0.60 m length and 0.15 m diameter, were installed to run the experiment. The bottles used for collecting leachate were installed on the floor. Filter papers were placed on porous bottom of the columns to prevent soil leaching. The columns were connected with bottles using small pipes for the collection of leachate. The connecting pipes were kept airtight to prevent evaporation from the leachate bottles. Locally collected sandy soil samples from agricultural land (0-12 cm depth) were utilized for the experiment. Bentonite material was commercially purchased and then air-dried. Both the soil and bentonite were analyzed for physico-chemical properties before the experiment. After air drying, 46 kg of the soil was added to each column (0.50 m length). The air-dried clay was applied at the rate of 0, 2 and 4% to the sandy soil packed in a PVC column. Three treatments of nitrogen (N) fertilizers namely calcium nitrate [$Ca(NO_3)_2$], ammonium chloride [NH_4Cl], and urea [$CO(NH_2)_2$] were applied to the soil. Based on the bulk density of soil (1.3 g cm^{-1}), each fertilizer was applied at 300 kg N ha^{-1}. Initially, as

the soil was dry, the amount of first water application was kept higher so that enough water may drain out to collect the leachate. For later leaching events, the leaching fraction (LF) was calculated by dividing the drained water by applied water. Then, the tap water was applied at leaching fraction of 0.3~0.4. Leachate was collected within 24 h after each water application. A total of five leaching events were covered, that is, 1st, 2nd, 4th, 6th and 10th day. The graphical display of the experiment is illustrated in [Figure 1].

Figure 1 Graphical display of lysimeter experiment

2.2 Laboratory analysis

Chemical analyses of soil, bentonite and tap water were carried out before experiment. Soil samples were air-dried and sieved via 2 mm sieve. The soil and air-dried bentonite material were tested for pH and electrical conductivity (EC) in 1:5 (w/v) soil-water suspensions by a pH meter (Model: HANNA HI 8520) and EC meter (Model: 4320 JENWAY), respectively [46]. The soil was saturated for overnight, weighed and then the water holding capacity (WHC) of the soil was calculated by the difference in the weight of soil [47]. The bulk density of soil was determined using cylindrical cores. The soil sample was weighed and placed in the oven at 105℃ for 8 to 12 hours until the weight was constant. Bulk density was then calculated in the same way as described by Grossman and Reinsch [48].

The post-experiment soil was sampled in two layers (0-25 and 25-50 cm) from the PVC columns and was thoroughly mixed. A 10 g soil sample was shaken in 100 mL distilled water for 1 h and then the suspension was filtered. Moisture content in the soil samples was adjusted by oven drying few grams of soil. The leachate collected from 5 events was analyzed for NO_3 concentration. The NO_3^{-1} concentration in pre- and post-experiment soil, bentonite and leachate

was determined by UV spectrophotometer (Model: LI-UV-7000) at 220 nm [49]. All the reagents/chemicals of Sigma Aldrich, Germany, were utilized during the experiment.

2.3 Statistical analysis

Data were statistically analyzed by OriginLab 2021 for graphical presentation. The three-way analysis of variance (ANOVA) was performed on Sigmaplot. The three factors were taken as bentonite (0, 2% and 4%), N fertilizer sources ([$Ca(NO_3)_2$], [NH_4Cl] and [$CO(NH_2)_2$]), and leaching events (1st, 2nd, 4th, 6th and 10th day) with three replications. A post hoc Tukey test was also performed to determine the significant difference between the levels of factors.

3. Results

3.1. Pre-experiment chemical analysis

Bentonite clay, soil and water were analyzed for chemical properties before experiment, which are presented in Table 1. Analysis revealed that soil had highest NO_3^{-1} concentrations, compared to bentonite clay. The tap water had low concentrations of NO_3^{-1}. The electrical conductivity (EC) of bentonite was highest compared to soil and water samples, but still it fell below the category of non-saline (EC<4 dS m^{-1}). However, the pH of bentonite was lower (pH<7) making it more acidic as compared to soil and water.

Table 1 Nitrate, electrical conductivity (EC) and pH of bentonite clay material, soil and tap water

Material	Nitrate	EC (mS m^{-1})	pH
Bentonite	23.5 mg kg^{-1}	126.7	5.7
Soil	34.4 mg kg^{-1}	87.8	7.8
Tap water	7.8 mg L^{-1}	37.5	7.2

3.2. Effect of bentonite and N sources on NO_3^{-1} leaching

Impact of bentonite clay mixed with different N sources on NO_3^{-1} leaching is illustrated in [Figure *2*]. Results showed NO_3^{-1} concentration decreased by 12 to 19% in the leachate, irrespective of the source of N with increasing bentonite rates. The significantly highest reduction (20-25%) in NO_3^{-1} leaching was recorded with $Ca(NO_3)_2$ with 4% bentonite as compared with $CO(NH_2)_2$ and NH_4Cl at similar bentonite rates. At early stages of leaching, the leachate showed higher concentrations of NO_3^{-1} in leachate with both NH_4Cl and $Ca(NO_3)_2$, but leaching of NO_3 increased with urea sources at later leaching stages [Figure 3]. Total NO_3^{-1} loads were higher in soil with urea and $Ca(NO_3)_2$ treated soil at 4% bentonite as compared to NH_4Cl [Figure 4]. The incubation of soil with bentonite (4%) reduced NO_3 content by 7%, 20% and 8% with $Ca(NO_3)_2$, NH_4Cl and $CO(NH_2)_2$ treated soil, respectively [Figure *5*].

Figure 2 Average amount of leachate nitrate from bentonite treated sandy soil (from day 1 to day 10). N, A and U indicate $Ca(NO_3)_2$, NH_4Cl and $CO(NH_2)_2$, respectively. Bentonite clay was applied at the rate of 0%, 2% and 4%. *, ** and *** show significant differences at P<0.05, P<0,01 and P<0.001, respectively

Figure 3 Nitrate leaching in bentonite amended sandy soil using $Ca(NO_3)_2$ (a), NH_4Cl (b), and $CO(NH_2)_2$ (c) with 0%, 2% and 4% bentonite during five leaching events (days). *, ** and *** show significant difference at P<0.05, P<0,01 and P<0.001, respectively

Figure 4 Residual nitrate loads (g) in soil after leaching events (days) at 0%, 2% and 4% bentonite with $Ca(NO_3)_2$ (N), NH_4Cl (A), and $CO(NH_2)_2$ (U). *, ** and *** show significant difference at P<0.05, P<0,01 and P<0.001, respectively

Figure 5 Nitrate concentration in 4% bentonite clay mixed sandy soil after discrete incubation with $Ca(NO_3)_2$ (N), NH_4Cl (A) and $CO(NH_2)_2$ (U), *, ** and *** show significant difference between treatments at P<0.05, P<0,01 and P<0.001, respectively

3.3. Statistical Analysis

A three-way analysis of variance (ANOVA) was performed on NO_3^{-1} leaching with 3 N sources, 3 levels of bentonite and 5 leaching events [Table 2]. Analysis showed that there was significant difference (P<0.001) within N sources, bentonite levels and leaching events (days). There was statistically significant (P<0.01) interaction among all the factors. The Tukey test revealed significant (P<0.05) difference between 0% and 2%, and 0% and 4% bentonite with all N sources on NO_3^{-1} leaching, but the difference was not significant between 2% and 4% bentonite with all N sources [Table 2].

Table 2 Summary ANOVA on effect of bentonite and N sources on NO_3^{-1} leaching at different leaching events

Source of Variation	DF	SS	MS	F	P
N sources (N)	2	2943336.13	1471668	14453	< 0.01
Bentonite levels (B)	2	74157.91	37078	364	< 0.01
Leaching event / day (D)	4	282061.82	70515	692	< 0.01
N x B	4	11270.48	2817	27	< 0.01
N x D	8	589355.42	73669	723	< 0.01
B x D	8	15955	1994	19	< 0.01
N x B x D	16	24125	1507	14	< 0.01
Residual	90	9164	101		
Total	134	3949426	29473		

4. Discussion

Results showed that irrespective of the source of N, the NO_3^{-1} leaching consistently decreased with increasing bentonite application showing the sequences of fertilizer type as: $Ca(NO_3)_2$ > NH_4Cl > $CO(NH_2)_2$ [Figure *2*]. Form of N leached from the soil columns was closely related to the type of fertilizer applied to the soil [50]. The NO_3^{-1} fertilizers appeared to be more sensitive to the leaching, especially in sandy soils [51] and also to the denitrification [52] as compared to urea or ammonium fertilizers. Also, NO_3^{-1} leaching is substantially higher in free-drained soils [53], such as sandy soil with macropores used in the present study.

Application of bentonite decreased NO_3^{-1} leaching, regardless to the rate of application. The [NO_3^{-1}] decreased with increasing bentonite treatments level. Whereby higher NO_3^{-1} concentration was observed in NO_3^{-1} and NH_4 containing fertilizers during the initial leaching [Figure 3]. Urea form of N showed consistent increases in NO_3^{-1} concentrations in water collected in later leaching stages [Figure 3]. This could be associated with increased nitrification process in soils under unsaturated conditions [54], which might have resulted in increased NO_3^{-1} leaching at later stages. Accumulation of NO_3^{-1} was more in the soil sampled from the lower layer of the column after a leaching process, showing the sequence as $CO(NH_2)_2$ > NH_4Cl > $Ca(NO_3)_2$.

The amount of total NO_3^{-1} retained in soil was termed as nitrate loads conversely related to the amount of NO_3^{-1} in the leachates [Figure 4]. An enhanced application of bentonite significantly retained NO_3^{-1} in the soil columns. A higher amount of NO_3^{-1} was retained in the soil amended with 4% bentonite. The application of bentonite clay enhanced soil moisture and improved macro-aggregate development [55] which improved soil quality through structural development, by increased exchange of anions and cations [56] and helped in reduced leaching while promoting nutrient retention [57].

Enrichment of sandy soils with bentonite increased the porosity and altered the pore-size distribution [58]. The interactions of bentonite with biochar and urea improved soil properties by diffusing soil moisture which controlled the mobility of nutrients within soils [59], thus with high water retaining capacity, increased exchange capacity, swelling, thermal stability and slow-releasing characteristics, bentonite offers valuable solution to reduced nutrient leaching from loose soils.

Higher quantity of NO_3^{-1} was retained in urea treated soil followed by nitrate and ammonium containing fertilizers [Figure 4]. This could be attributed to the transformation of NO_3^{-1} in urea contained soil after few days of incubation. Across all N sources, the application of bentonite (4%) markedly limited the release of NO_3^{-1}. After incubation, bentonite contents reduced the magnitude of NO_3^{-1} among fertilizers as follow: 7% in $Ca(NO_3)_2$, 20% in NH_4Cl and 8% in urea treated soil [Figure 5]. The soil having negatively charged sites attracts more positively charged NH_4 as compared to the negatively charged NO_3^{-1}, and therefore the NH_4 has been considered to be a less mobile in soils, than NO_3^{-1} [60, 61].

The effect of bentonite on the leachability of NO_3^{-1} varied with different N fertilizers. The NO_3^{-1} leaching consistently decreased with increasing bentonite application showing the sequences of fertilizer type as: $Ca(NO_3)_2$ > NH_4Cl > $CO(NH_2)_2$ [Figure 2]. Addition of Ca as $Ca(NO_3)_2$ increased the adsorptive capacity of bentonite at low pH (5-6), while higher concentration of NO_3^{-1} ion due to the addition of calcium nitrate as N source resulted in increased adsorption of NO_3^{-1} at bentonite clay surfaces [62]. The mechanism can be further explained by Ca hydrolysis, which resulted into the formation of less soluble $Ca(OH)_2$, releasing more H^+ ions, thus acidifying the media [Eq 1][63]. Under low pH, the anion exchange capacity of bentonites in significantly increased which offers more positive sites to attract NO_3^{-1} on its surface [Eq 2], which

increased the retention of NO_3^{-1} ions on soil colloids due to adsorption phenomenon [Eq 3]. This ultimately reduced NO_3^{-1} leaching from sandy soil and showed lower NO_3^{-1} concentrations in leachate. The entire process can be explained as follows, where A is dissociated anion and X is the soil colloidal surface

$$Ca^{2+} + H_2O \rightleftharpoons Ca(OH)_2 + 2H^+ \qquad \text{[Eq 1]}$$

$$X + H^+ \rightleftharpoons X^+ + HA \qquad \text{[Eq 2]}$$

$$X^+ + NO_3^- \rightleftharpoons XNO_3 \qquad \text{[Eq 3]}$$

Surface charge of variable charge clays varies with changes in pH of soil solution, therefore, at low pH, the anion exchange capacity exceeds cation exchange capacities which retain more NO_3^{-1} on its surfaces [64], while increasing the mobility of NH_4 in such conditions. However, the mobility of both N forms can be maintained by adjusting the rates of $Ca(NO_3)_2$ and urea along with bentonite clay under field conditions. The study suggested that bentonite amendment with $Ca(NO_3)_2$ as N sources could effectively mitigate NO_3^{-1} leaching from sandy soils.

5. Conclusions

It is concluded that N sources and bentonite application were important factors affecting the NO_3^{-1} leaching from sandy soil. Application of N sources enhanced NO_3^{-1} leaching from the sandy soil. The NO_3^{-1} leaching decreased in the order of $Ca(NO_3)_2$ > NH_4Cl > urea. Bentonite substantially reduced NO_3^{-1} in the leachate. Urea showed higher NO_3^{-1} at the later leaching events. Higher contents of NO_3^{-1} were retained in the soil with 4% bentonite. Higher NO_3^{-1} contents were accumulated in the lower part of the soil column after a leaching process. This experiment suggests that bentonite clay chemistry with added $Ca(NO_3)_2$ provide better understanding of anion exchange capacity to retain higher NO_3^{-1} concentrations in soil, thereby decreasing NO_3^{-1} leaching from

sandy soil. Further research is suggested to investigate the effects of different clay types on the dynamics of nitrate under field conditions.

Authors Contributions

Z. Hussain., M. Irshad., R.A. Khattak. led the research study and prepared the manuscript. M. Mohiuddin., T. Cheng., C. Yao. analyzed the data and prepared graphs. D. Song. facilitated the research. R.A. Khattak edited the manuscript for technical inputs. All the authors discussed and reviewed the manuscript.

References

1. Elferink M, Schierhorn F (2016) Global demand for food is rising. Can we meet it? Harvard Business Review, Harward Business Publishing, Canada.
2. Bijay-Singh, Craswell E (2021) Fertilizers and nitrate pollution of surface and ground water: an increasing pervasive global problem. SN Applied Science 3: 518. https://doi.org/10.1007/s42452-021-04521-8
3. Shah AN, Iqbal J, Tanveer M et al. (2017) Nitrogen fertilization and conservation tillage: a review on growth, yield, and greenhouse gas emissions in cotton. Environmental Science and Pollution Research 24: 2261–2272. https://doi.org/10.1007/s11356-016-7894-4.
4. Mosongo PS, Pelster DE, Li X, Gaudel G, Wang Y, Chen S, et al. (2022) Greenhouse Gas Emissions Response to Fertilizer Application and Soil Moisture in Dry Agricultural Uplands of Central Kenya. Atmosphere 13: 463. https://doi.org/10.3390/atmos13030463
5. Houlton BZ, Almaraz M, Aneja V, Austin AT, Bai E, Cassman KG, et al. (2019) A world of cobenefits: Solving the global nitrogen challenge. Earth's Future 7: 865–872.
6. Xu P, Chen A, Houlton BZ, Zeng Z, Wei S, Zhao C, et al. (2020) Spatial Variation of Reactive Nitrogen Emissions from China's Croplands Codetermined by Regional

Urbanization and Its Feedback to Global Climate Change. Geophysical Research Letters 47: e2019GL086551.

7. Pathak H, Jain N, Bhatia A, Kumar A, Chatterjee D, et al. (2016) Improved nitrogen management: A key to climate change adaptation and mitigation. Indian Journal of Fertilizers 12: 151–162.

8. Lu J, Bai Z, Velthof GL, Wu Z, Chadwick D, Ma L (2019) Accumulation and leaching of nitrate in soils in wheat-maize production in China. Agricultural Water Management 212: 407-415. https://doi.org/10.1016/j.agwat.2018.08.039

9. Kladivko EJ, Van Scoyoc GE, Monke EJ, Oates KM, Pask W (1991) Pesticides and nutrient movement into subsurface tile drains on s silt loam soil in Indiana. Journal of Environmental Quality 20: 264-270.

10. Jacinthe PA, Dick WA, Brown LC (1999) Bioremediation of nitrate-contaminated shallow soils using water table management techniques: Nitrate removal efficiency. Transactions of the American Society of Agricultural Engineers 42: 1251-1259.

11. Bibi S, Saifullah, Naeem A, Dahlawi S (2016) Environmental Impacts of Nitrogen Use in Agriculture, Nitrate Leaching and Mitigation Strategies. Hakeem et al. (eds.), Soil Science: Agricultural and Environmental Prospectives, Springer International Publishing Switzerland. https://doi.org/10.1007/978-3-319-34451-5_6

12. Tamagno S, Eagle AJ, McLellan E, van Kessel C, Linquist B A, Ladha JK et al. (2022) Quantifying N leaching losses as a function of N balance: A path to sustainable food supply chains. Agriculture, Ecosystems and Environment 324: 107714. https://doi.org/10.1016/j.agee.2021.107714

13. Hallaq AHA (2010) The impact of soil texture on nitrates leaching into groundwater in the North Governorate, Gaza Strip. Journal of Social Sciences 38(2): 1-37.

14. Plošek L, Elbl J, Lošák T, Kužel S, Kintl A, Juřička D et al. (2017) Leaching of mineral nitrogen in the soil influenced by addition of compost and N-mineral fertilizer. Acta Agriculturae Scandinavica, Section B. Soil and Plant Science 67(7): 607-614. https://doi.org/10.1080/09064710.2017.1322632

15. Huddell AM, Galford GL, Tully KL, Crowley C, Palm CA, Neill C et al. (2020) Meta-analysis on the potential for increasing nitrogen losses from intensifying tropical agriculture. Global Change Biology 26: 1668-1680. https://doi.org/10.1111/gcb.14951

16. Hatfield JL, Cambardella CA (2001) Nutrient management in cropping systems. In McFarland J and Sanderson M (ed.) Integrated management of land application of animal waste. (In press.). American Society of Agricultural Engineers, St. Joseph, MI.

17. Rasa K, Heikkinen J, Hannula M, Arstila K, Kulju S, Hyvaluoma J (2018) How and why does willow biochar increase a clay soil water retention capacity? Biomass and Bioenergy 119: 346-353. 10.1016/j.biombioe.2018.10.004

18. Wang H, Gao J-e, Li X-h, Zhang S-l, Wang H-j (2015) Nitrate Accumulation and Leaching in Surface and Ground Water Based on Simulated Rainfall Experiments. PLoS ONE 10(8): e0136274. https://doi.org/10.1371/journal.pone.0136274

19. Gatiboni L, Osmond D (2019) Nitrogen Management and Water Quality, Publication No: AG-439-02, NC State Extension Publications, NC State University, USA.

20. Johnson S, Burchell MR, Evans RO, Osmond DL, Gilliam JW (2012) Riparian Buffer Located in an Upland Landscape Position Does Not Enhance Nitrate-nitrogen Removal. Ecological Engineering 52: 252-261. http://dx.doi.org/10.1016/j.ecoleng.2012.11.006.

21. King SE, Osmond DL, Smith J, Dukes M, Evans RO, Knies S et al. (2015) Effects of Riparian Buffer Vegetation and Width: A 12-year Retrospective Study. *Journal of Environmental Quality* 45(4): 1243-1251. https://doi.org/10.2134/jeq2015.06.0321

22. Hansen B, Thorling L, Schullehner J et al. (2017) Groundwater nitrate response to sustainable nitrogen management. Scientific Reports 7: 8566. https://doi.org/10.1038/s41598-017-07147-2

23. Ayyub CM, Haidar MW, Zulfiqar F, Abideen ZU, Wright SR (2019) Potato tuber yield and quality in response to different nitrogen fertilizer application rates under two split doses in an irrigated sandy loam soil. Journal of Plant Nutrition 42(15): 1850-1860. https://doi.org/10.1080/01904167.2019.1648669

24. Alexander RB, Smith RA, Schwarz GE (1995) The regional transport of point and non-point source of nitrogen to the Gulf of Mexico. In Proc. Gulf of Mexico Hypoxia Conf. Kenner LA, 5-6 Dec 1995. USEPA Publication National Center for Environment. Publ. and Inf. Washington, D.C.

25. Zeb S, Waseem A, Malik AH, Mahmood Q (2011) Water quality assessment of Siran River, Pakistan. International Journal of Physical Sciences 6: 7789 – 7798.

26. Kanwar RS, Stilenberg DER, Pfeiffer DL, Karlen TS, Simpkins WW (1993) Transport of nitrate and pesticides to shallow groundwater systems as affected by tillage and crop rotation practices, 270-273. In Proc. National Conference on Agricultural Research to Protect Water Quality.

27. Randall GW (1997) Nitrate-N in surface waters as influenced by climatic conditions and agricultural practices. In Proc. Agriculture. and Hypoxia in the Mississippi Watershed

Conference, St. Louis, MO. 14–15 July 1997. American Farm Bureau Federation, Park Ridge, IL.

28. Cambardella CA, Moorman TB, Jaynes DB, Parkin TB, Karlen DL (1999) Water quality in Walnut Creek watershed: Nitrate nitrogen in soils, subsurface drainage water and shallow groundwater. Journal of Environmental Quality 28: 25-34.

29. EPA (2022) National Primary Drinking Water Regulations. Available at: https://www.epa.gov/ground-water-and-drinking-water/national-primary-drinking-water-regulations#Inorganic (Date of access: 28.08.2022).

30. Kross BC, Hallberg GR, Bruner R, Cherryholmes K, Johnson KJ (1993) The nitrate contamination of private well water in Iowa. American Journal of Public Health 83: 270-272.

31. WHO (2022) Guidelines for drinking-water quality 4th ed., Available at: https://www.who.int/publications/i/item/9789240045064 (Date of access: 28.08.2022).

32. Espejo-Herrera N et al. (2016) Colorectal cancer risk and nitrate exposure through drinking water and diet. International Journal of Cancer 139: 334–346. https://doil.org/10.1002/ijc.30083.

33. Jones RR et al. (2016) Nitrate from Drinking Water and Diet and Bladder Cancer Among Postmenopausal Women in Iowa. Environmental Health Perspectives 124: 1751–1758. https://doi.org/10.1289/EHP191.

34. Irshad M, Waseem A, Umar M, Sabir MA (2014) Leachability of nitrate from sandy soil using waste amendment. Communications in Soil Science and Plant Analysis 45: 680-687.

35. Sirivastava A, Singh PK (2017) Adsorption of Nitrate from Ground Water using Indian Bentonite: Fixed Bed Column Study. International Journal of Engineering Research and Technology (IJERT) 6(5): 390-394.

36. Bentahar S, Dnik A, Khomri ME, Messaoudi NE, Mohammad AD, Noureddine E et al. (2018) Removal of a cationic dye from aqueous solution by natural clay. Journal of Groundwater for Sustainable Development 6: 255–262. https://doi.org/10.1016/j.gsd.2018.02.002

37. De Castro MLFA, Abad MLB, Sumalinog DAG, Abarca RRM, Paoprasert P, de Luna MDG (2018) Adsorption of Methylene Blue dye and Cu(II) ions on EDTA-modified bentonite: Isotherm, kinetic and thermodynamic studies. J. Sustain. Environ. Res. 28, 197–205. https://doi.org/10.1016/j.serj.2018.04.001

38. Lopez-Fernandez M, Vilchez-Vargas R, Jroundi F, Boon N, Pieper D, Merroun ML (2018) Microbial community changes induced by uranyl nitrate in bentonite clay microcosms. Applied Clay Science 160: 206-216. https://doi.org/10.1016/j.clay.2017.12.034

39. Hall DJM, Jones HR, Crabtree WL, Daniels TL (2010) Claying and deep ripping can increase crop yields and profits on water repellent sands with marginal fertility in southern Western Australia. Australian Journal of Soil Research 48: 178–187. https://doi.org/10.1071/SR09078.

40. Mi J, Gregorich EG, Xu S, McLaughlin NB, Liu J (2020) Effect of bentonite as a soil amendment on field water‐ holding capacity, and millet photosynthesis and grain quality. Scientific Reports 10: 18282. https://doi.org/10.1038/s41598-020-75350-9.

41. Czaban J, Siebielec G (2013) Effects of Bentonite on Sandy Soil Chemistry in a Long-Term Plot Experiment (II); Effect on pH, CEC, and Macro- and Micronutrients. Polish Journal of Environmental Studies 22(6): 1669-1676.

42. Zulfiqar F, Navarro M, Ashraf M, Akram NA, Munné-Bosch S (2019) Nanofertilizer use for sustainable agriculture: Advantages and limitations. Plant Science 289: 110270. https://doi.org/10.1016/j.plantsci.2019.110270.

43. Tahir S, Marschner P (2017) Clay Addition to Sandy Soil Reduces Nutrient Leaching—Effect of Clay Concentration and Ped Size. Communications in Soil Science and Plant Analysis 48(15): 1813–1821. https://doi.org/10.1080/00103624.2017.1395454.

44. Shi W, Ju Y, Bian R, Li L, Joseph S, Mitchell RG et al. (2020) Biochar bound urea boosts plant growth and reduces nitrogen leaching. Science of Total Environment 701: 134424. https://doi.org/10.1016/j.scitotenv.2019.134424

45. Zhao C, Hu C, Huang W, Sun X, Tan Q, Di H (2010) A lysimeter study of nitrate leaching and optimum nitrogen application rates for intensively irrigated vegetable production systems in Central China. Journal of Soils and Sediments 10: 9–17.

46. Richard LA (1954) Diagnosis and improvement of saline and alkali soils (USDA Handbook 60) Washington, DC: USDA.

47. Basso AS, Miguez FE, Laird DA, Horton R, Westgate M (2013) Assessing potential of biochar for increasing water-holding capacity of sandy soils. GCB-Bioenergy 5(2): 132-142. https://doi.org/10.1111/gcbb.12026

48. Grossman RB, Reinsch TG (2002) Bulk density and linear extensibility: core method. In: Dane JH, Topp GC (Eds.), Methods of Soil Analysis. Part 4. Physical Methods. Soil Science Society of America, Inc., Madison, WI, pp. 208–228.

49. Norman RJ, Stucki JW (1981) The determination of nitrate and nitrite in soil extracts by ultraviolet spectrophotometry. Soil Science Society of America 45: 347-353.

50. Zhou JB, Xi JG, Chen ZJ, Li SX (2006) Leaching and transformation of nitrogen fertilizers in soil after application of N with irrigation: A soil column method. Pedosphere 16(2): 245-252.

51. De Boer HC, Deru JGC, Hoekstra NJ, Van Eekeren N (2016) Strategic timing of nitrogen fertilization to increase root biomass and nitrogen-use efficiency of *Lolium perenne L*. Plant and Soil 407: 81-90.

52. Harty MA, Forrestal PJ, Watson CJ, McGeough KL, Carolan R, Elliot C et al. (2016) Reducing nitrous oxide emissions by changing N fertilizer use from calcium ammonium nitrate (CAN) to urea based formulations. Science of Total Environment 563-564: 576-586.

53. De Boer HC (2017) Nitrate leaching from liquid cattle manure compared to synthetic fertilizer applied to grassland or silage maize in the Netherlands. Wageningen Livestock Research Wageningen, October.

54. Zhang YM, Chen DL, Zhang JB, Edis R, Hu CS, Zhu AN (2004) Ammonia volatilization and denitrification losses from an irrigated maize-wheat rotation field in the North China Plain. Pedosphere 14(4): 533-540.

55. Zhang H, Chen W, Zhao B, Phillips LA, Zhou Y, Lapen DR et al. (2020) Sandy soils amended with bentonite induced changes in soil microbiota and fungistasis in maize fields. Applied Soil Ecology 146: 103378. https://doi.org/10.1016/j.apsoil.2019.103378.

56. Churchman GJ, Noble A, Bailey G, Chittleborough D, Harper R (2014) Clay addition and redistribution to enhance carbon sequestration in soils Soil Carbon. In: Hartemink AE,

McSweeney K (eds.) Soil Carbon. Springer International Publishing, Cham, Switzerland, pp. 327-335. https://dx.doi.org/10.1007/978-3-319-04084-4_34 .

57. Satje A, Nelson PN (2009) Bentonite treatments can improve the nutrient and water holding capacity of sugarcane soils in the wet tropics. Proceedings: Australian Society of Sugarcane Technology 31: 166-176.

58. Suzuki S, Noble AD, Ruaysoongnern S, Chinabut N (2007) Improvement in water-holding capacity and structural stability of a sandy soil in northeast Thailand. Arid Land Research Management 21: 37-49.

59. Liu X, Liao J, Song H et al. (2019) A Biochar-Based Route for Environmentally Friendly Controlled Release of Nitrogen: Urea-Loaded Biochar and Bentonite Composite. Scientific Reports 9: 9548. https://doi.org/10.1038/s41598-019-46065-3.

60. Wang FL, Alva AK (2000) Ammonium adsorption and desorption in sandy soils. Soil Science Society of American Journal 64: 1669-1674.

61. Wang Y, Zhang YP (2004) Ammonium adsorption in a Eum-Orthic Anthrosol at different solution / soil ratios and temperature. Pedosphere 14(2): 253-257.

62. Ouakouak A, Youcef L, Achour S, Hamdi N (2018) Kinetics and equilibrium studies of nitrates adsorption onto natural Bentonite. Conference paper, Colloque International Sciences & Techniques de l'Eau & Environnement (STEE).

63. Lohinger H (2019) Calcium Hydroxide. Epina ebook Series. UC Davis, University of California, USA

64. Sollins P, Robertson GP, Uehara G (1988) Nutrient Mobility in Variable- and Permanent-Charge Soils. Biogeochemistry 6(3): 181-199.

Supplementary Files

Supplementary File
Bentonite experimer

Figure 1

Click here to access/download;Figure;Figure 1.tif

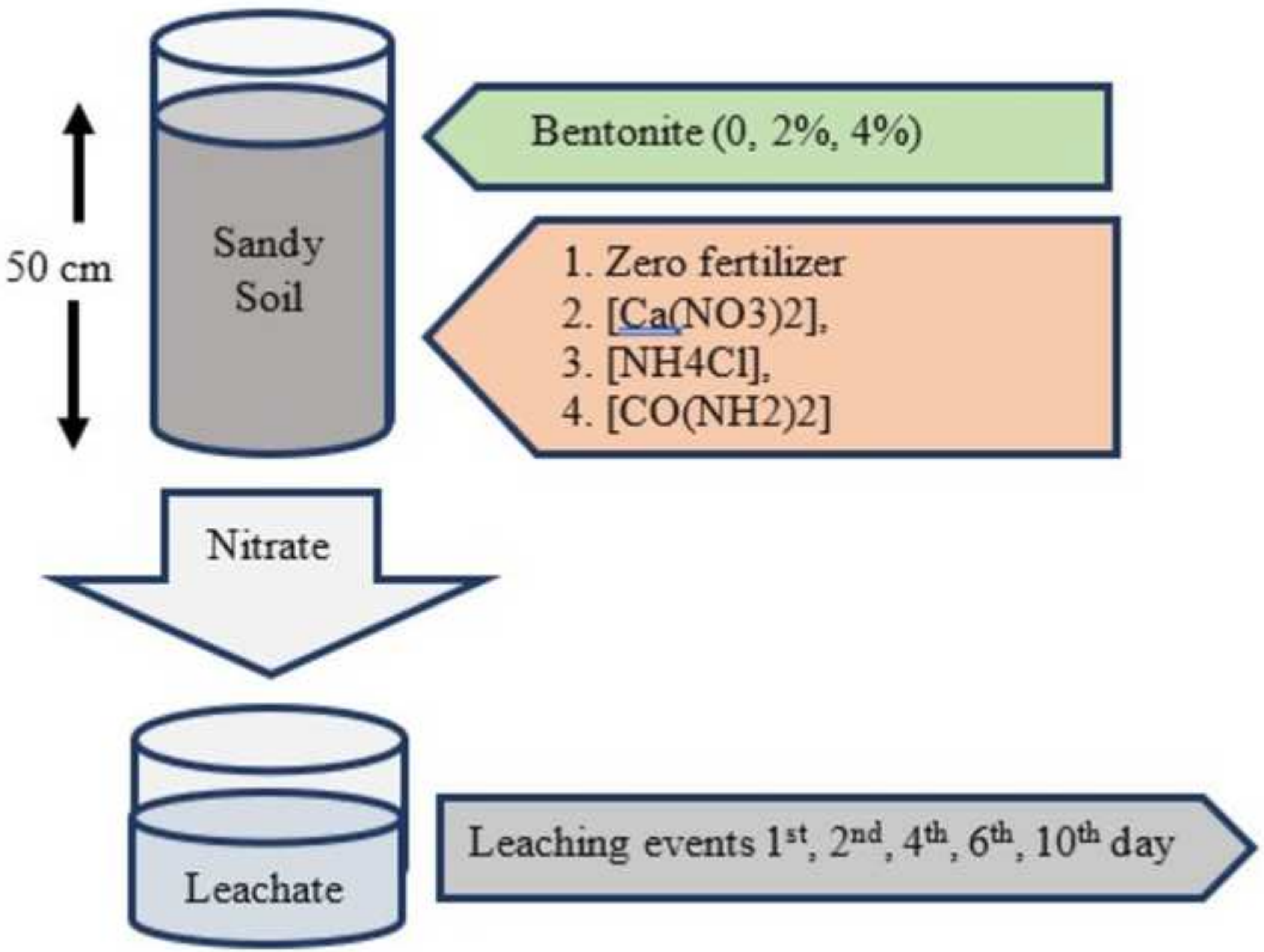

Figure 2

Click here to access/download;Figure;Figure 2.tif

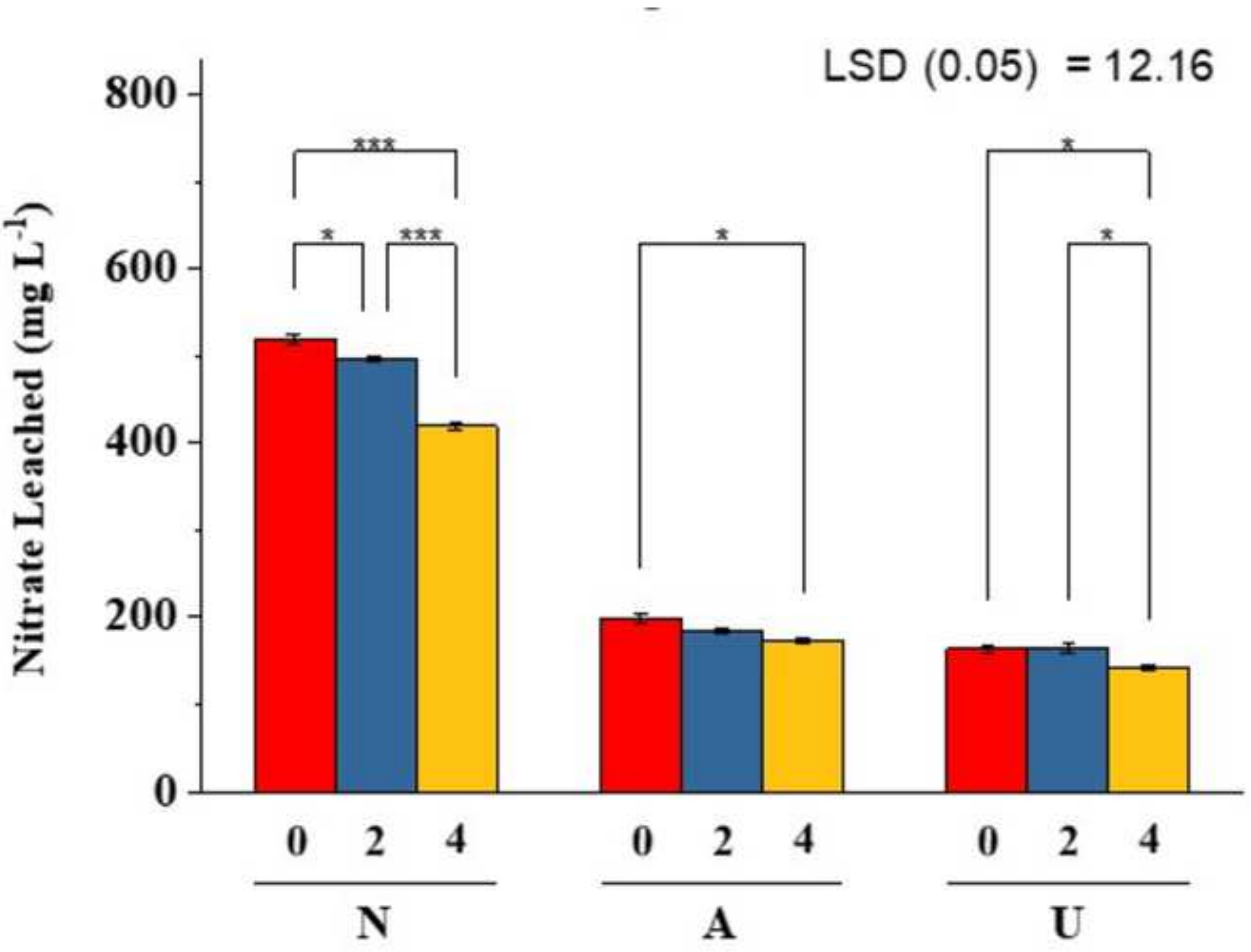

Figure 3

Click here to access/download;Figure;Figure 3.tif

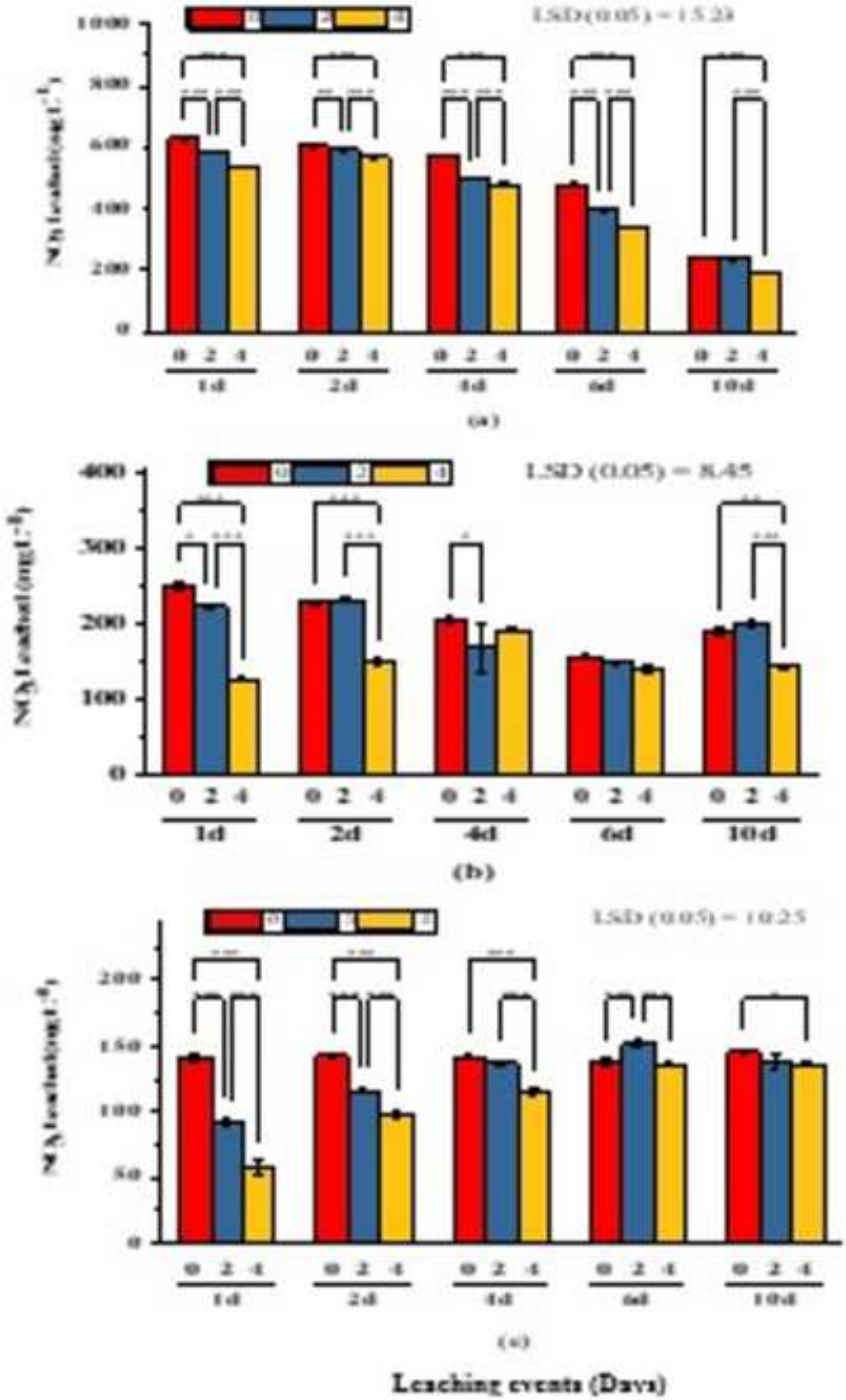

Figure 4

Click here to access/download;Figure;Figure 4.tif

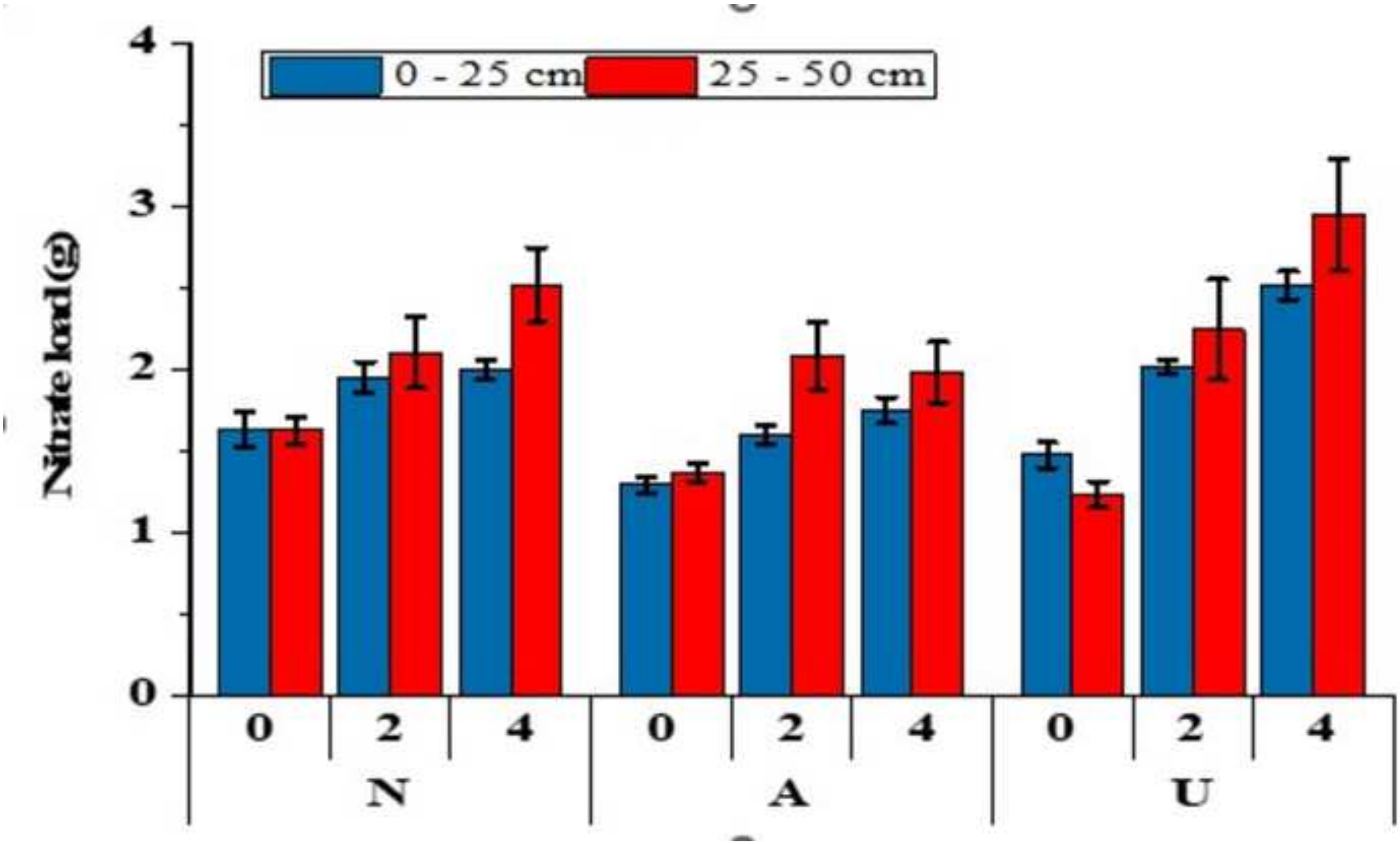

Figure 5

Click here to access/download;Figure;Figure 5.tif

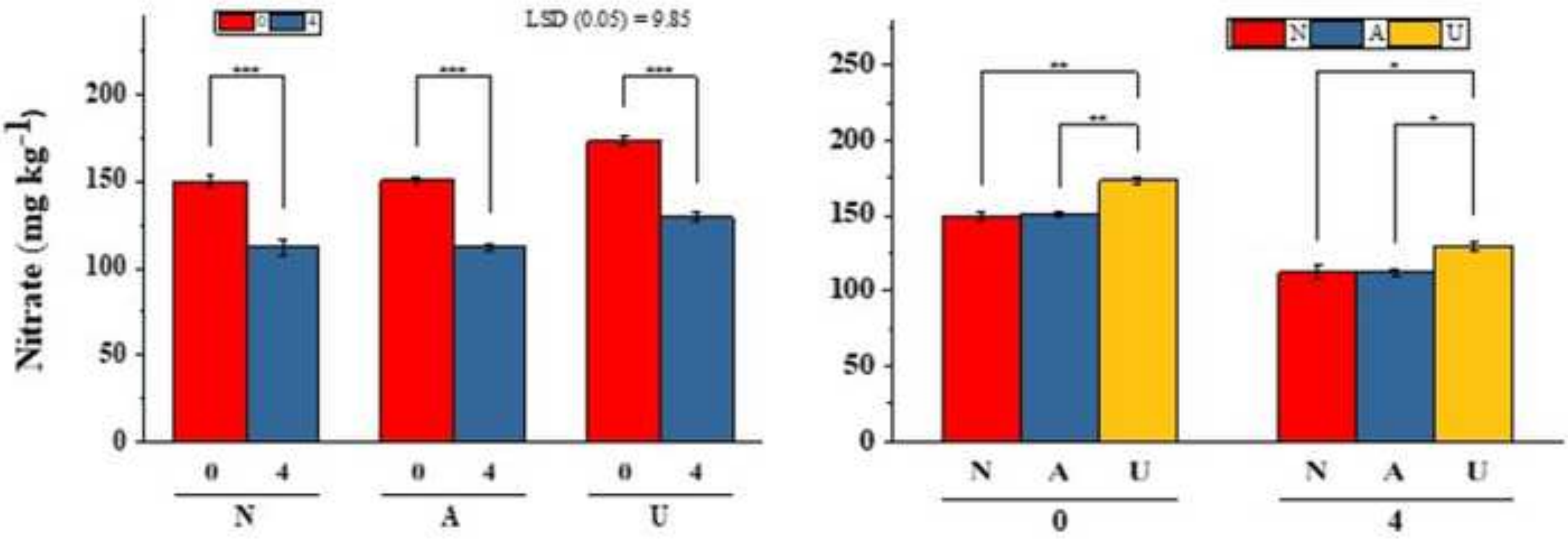

Click here to access/download
Supporting Information
Supplementary File Bentonite experiment data.docx

Bentonite clay ~~mixed~~ with different ~~N~~nitrogen sources ~~significantly~~ reduce nitrate leaching from sandy soil

Zahid Hussain[a,b,*], Tang Cheng[a,*], Muhammad Irshad[c,], Riaz A. Khattak[d], Chen Yao[e], Di Song[a], Muhammad Mohiuddin[f,]

[a]Yunnan key Laboratory of Pollution Process and Management of Plateau-Lake -Watershed, Yunnan Research Academy of Eco-Environmental Sciences, Kunming, 650034, China

[b]Department of Development Studies, COMSATS University Islamabad (CUI), Abbottabad Campus, Abbottabad 22060, Pakistan

[c]Department of Environmental Sciences, CUI, Abbottabad Campus, Abbottabad 22060, Pakistan

[d]The Brains Institute, Peshawar, Pakistan

[e]Yunnan Infrastructure Investment Co. Ltd., 650599, China

[f]Department of Environmental Sciences, Kohsar University, Murree, Pakistan

[*]Corresponding Authors:

1. Dr. Zahid Hussain (Department of Development Studies, COMSATS University Islamabad, Abbottabad Campus, Abbottabad, Pakistan) (Present Address), (Email: drzahid@cuiatd.edu.pk)

2. Tang Cheng (Yunnan key Laboratory of Pollution Process and Management of Plateau-Lake - Watershed, Yunnan Research Academy of Eco-Environmental Sciences, Kunming, 650034, China, Email: 1290992119@qq.com)

Highlights

- Sandy soils are more prone to nutrient leaching
- Application of N sources enhanced NO_3 leaching from the sandy soil
- NO_3 leaching reduces soil fertility and causes groundwater pollution
- Bentonite amendment significantly reduced NO_3 leaching from sandy soil
- The NO_3 leaching decreased with N sources in the order as $Ca(NO_3)_2$>NH_4Cl> $CO(NH_2)_2$

Abstract

Nitrate (NO_3^{-1}) leaching from soils results in the lower soil fertility, reduced crop productivity and increased water pollution. The effects of bentonite clay on NO_3^{-1} leaching from sandy soils are not extensively studied. Therefore, the present lysimeteric study determined NO_3^{-1} leaching from bentonite [0, 2 and 4% (m/m)] treated sandy soil under three Nitrogen (N) sources (calcium nitrate [$Ca(NO_3)_2$], ammonium chloride [NH_4Cl], and urea [$CO(NH_2)_2$] at the rate of 300 kg N ha^{-1}). Results showed that bentonite markedly reduced NO_3^{-1} release in the leachate, while 4% bentonite retained higher NO_3 in the soil. The NO_3^{-1} leaching from sandy soil varied with N sources as $Ca(NO_3)_2 > NH_4Cl > (CO(NH_2)_2$. At early stages of leaching, higher concentrations of NO_3^{-1} were detected in leachate with both NH_4Cl and $Ca(NO_3)_2$, but leaching of NO_3^{-1} increased with urea at later leaching stages. The amount of total NO_3^{-1} retained in soil was conversely related to the amount of NO_3^{-1} in the leachate. This study indicated that soil amendment with bentonite could efficiently mitigate NO_3^{-1} leaching from sandy soil and hence prevent N fertilizer losses and groundwater pollution.

Key words: bentonite; nitrate leaching; N sources; sandy soil

1. Introduction

Growing populations and changing diets require an increase in agricultural production which ~~will/~~may lead to an increase in the use of fertilizers. As such, food demand and fertilizer use have been forecasted to double or triple by 2050 [1]~~(Elferink and Schierhorn, 2016)~~. Chemical N fertilizers and organic manures are often applied to the soil in higher amounts for higher agriculture production, which may lead to the N losses due to removal ~~Agricultural use of nitrogen (N) fertilizers is causing N emission~~ from the cropped fields into the water bodies [2]~~-~~ and/or

Formatted: Superscript
Formatted: Superscript
Formatted: Superscript
Formatted: Superscript
Formatted: Superscript
Formatted: Subscript
Formatted: Superscript
Formatted: Subscript
Formatted: Subscript
Formatted: Subscript
Formatted: Subscript
Formatted: Superscript
Formatted: Subscript
Formatted: Superscript
Formatted: Subscript
Formatted: Superscript
Formatted: Superscript
Formatted: Indent: First line: 0.5"
Formatted: Underline
Formatted: Underline

emission into the atmosphere [3, 4]. Of the applied N for crops, only 40-50% is being incorporated into the agricultural products [5] and the remainder N is subjected to the substantial losses [6]. The rainfall intensity and irrigation water influence the NO_3^{-1} loss in the soil profile [7, 8]. ~~This practice increased nutrient losses and resulted in tremendous N pollution with subsequent ecological damage.~~ The N fertilizers, used either as urea or ammonium form, is biochemically converted to NO_3^{-1} which is susceptible to leaching from soil-plant system and enter groundwater bodies [2]. ~~There is a general consensus that nitrate leaching from soils has caused substantial environmental threats. The primary source of N pollution in surface and ground water is mainly related to N fertilizers.~~ Therefore, an effective technology is required to prevent NO_3^{-1} losses from sandy soils. ~~Developing an effective technology to retain nutrients in soils is imperative to prevent NO_3 leaching from soils. Soil amendments have been considered as management practice to reduce NO_3 losses from sandy soils (Irshad et al., 2014). Bentonite, an alumina-siliceous clay material, has not been previously utilized to control NO_3 leaching.~~

Soil NO_3^{-1} is originated from both organic and inorganic N sources. Leaching and drainage studies found that NO_3^{-1} is the major form of N occurred in the soil water [8, 9, 10]~~(Kladivko et al., 1991; Jacinthe et al., 1999)~~. A number of factors including plant characteristics, seasonal and climatic changes, and soil properties govern NO_3 leaching from soils [11, 12]~~(Bibi et al., 2016)~~. The specific factors include soil texture, soil N concentration, amount of applied N, type of fertilizer, precipitation amount and intensity, soil water holding capacity, types of crops, root~~ing~~ length and N demand of next crop [13, 14]~~(Hallaq, 2010)~~. Leaching of NO_3^{-1}-N is more common than leaching of NH_4-N since both NO_3^{-1}-N and soil are negatively charged [15]. Over application or un-timely application of animal manures or commercial N fertilizers result in the nutrient imbalance in soils which lead to the increased N leaching rates, especially of NO_3^{-1}, into

Formatted: Underline
Formatted: Underline
Formatted: Underline
Formatted: Underline
Formatted: Subscript
Formatted: Superscript
Formatted: Underline
Formatted: Underline
Formatted: Subscript
Formatted: Superscript
Formatted: Underline
Formatted: Subscript
Formatted: Superscript
Formatted: Superscript
Formatted: Superscript
Formatted: Underline
Formatted: Underline
Formatted: Underline
Formatted: Underline
Formatted: Underline
Formatted: Underline
Formatted: Underline
Formatted: Superscript
Formatted: Superscript
Formatted: Underline
Formatted: Superscript

groundwater [12, 16]~~(Hatfield and Cambardella, 2001)~~. Sandy soils, due to low water holding capacity [17], allow NO_3^{-1} to leach down into the groundwater faster than the soils having fine textures, such as clay loams [13, 15]~~(Hallaq, 2010)~~. Thus, leaching of NO_3^{-1} through soil profile can potentially contaminate surface and groundwater [18]. Sandy soils with low organic matter may facilitate leaching of 10-15 mg L^{-1} of NO_3^{-1} to groundwater [19]. About 20-25% of this NO_3^{-1} may enter surface water via buffer streams and wetlands causing eutrophication of water bodies [20, 21].

The increasing unsustainable agricultural use ~~ed use~~ of N fertilizers results in NO_3 leaching into ground waters [22, 23] and runoff into surface water ecosystem producinge unfavorable consequences [24]~~(Alexander et al., 1995)~~, which adversely affect water quality [22, 25]~~(Zeb et al., 2011)~~. The increasing potential of contamination of water resources is linked with the inefficient management of N fertilizer when compared with the natural systems [26, 27, 28]~~(Kanwar et al., 1993; Randall, 1997; Cambardella et al., 1999)~~. The concentration of NO_3^{-1} above 10 mg L^{-1} in drinking water are considered as harmful for human health [29].~~The United States Environmental Protection Agency set the standard for drinking water and health advisory level of 10 mg L^{-1} nitrate based on the human health risks (Kross et al., 1993).~~ Higher NO_3^{-1} consumption has been affiliated with various illnesses, e.g., methemoglobinemia has been proven due to ingestion of over nitrate concentrations in water [30, 31]~~(Kross et al., 1993)~~. The endogenous NO_3^{-1} may chemically be transformed to carcinogenic N compounds leading to adverse effects of colorectal cancer [32] and bladder cancer [33]. Therefore, d~~D~~eveloping an effective technology to retain nutrients in soils is imperative to prevent NO_3^{-1} leaching from soils. Soil amendments have been considered as management practice to reduce NO_3^{-1} losses from sandy soils [34]~~(Irshad et al., 2014)~~. Bentonite, an alumina-siliceous clay material, has not been

Formatted: Underline
Formatted: Underline
Formatted: Underline
Formatted: Superscript
Formatted: Underline
Formatted: Underline
Formatted: Subscript
Formatted: Superscript
Formatted: Underline
Formatted: Underline
Formatted: Underline
Formatted: Underline
Formatted: Underline
Formatted: Underline
Formatted: Underline
Formatted: Underline
Formatted: Underline
Formatted: Underline
Formatted: Underline
Formatted: Underline
Formatted: Subscript
Formatted: Superscript
Formatted: Superscript
Formatted: Underline
Formatted: Superscript
Formatted: Underline
Formatted: Underline
Formatted: Subscript
Formatted: Superscript
Formatted: Underline
Formatted: Underline
Formatted: Superscript
Formatted: Superscript
Formatted: Underline

previously utilized to control NO_3^{-1} leaching.It is, therefore, imperative to mitigate the nutrient losses, especially NO_3 leaching from sandy soil to reduce health risk caused by groundwater contamination.

An alumina-siliceous clay material, bBentonite, like other clays, was never considered in reducing NO_3 leaching from sandy soils. Clays are hydrous aluminosilicates with fine colloids of < 2 mm of soils. Theysoils [35]. Clays are composed of fine-grained clays minerals and crystals such as quartz, carbonates and oxides [35](Sirivastava and Singh, 2017) and are. Clays are considered to retain contaminants by anion and cation exchange processes and prevent leaching into groundwaters. Due to effective adsorption capacities, bentonite clay has been used for multiple purposes. Bentonite is also used to remove dyes, radioactive waste, purification of viral RNA and of wastewater [36, 37, 38](Bentahar et al., 2018; De Castro et al., 2018; Lopez-Fernandez et al., 2018). Bentonite application as amendment enhanced soil fertility by increased soil carbon and potassium [39](Hall et al., 2010), while improved water holding capacity of sandy soils under drought stress [40](Mi et al., 2020). Bentonite application to sandy acidic soil improved soil fertility by increasing availability of macro-nutrient (up to 30%) to plants [41](Czaban, and Siebielec, 2013). Fertilizers, if used in combination with nano-dimensional adsorbents increase nutrient use efficiency and reduce nutrient leaching into groundwaters [42]. Clay amended sandy soil significantly reduced N and P leaching by 20% to 60% [43](Tahir and Marschner, 2017). Leaching of NH_4-N was reduced by 70% from a mixture of biochar, urea and bentonite plus sepiolite clay [44](Shi et al., 2020). However, it is still unclear that how the type of fertilizer and application of bentonite clay to soils can mitigate NO_3^{-1} leaching.

A reduction in the NO_3^{-1} leaching was expected when clay material was applied to the soil. Reports evaluating the interactive effects of bentonite material and N sources on the reduction of

Formatted: Superscript

Formatted: Indent: First line: 0.5"

Formatted: Underline

Formatted: Underline

Formatted: Underline

Formatted: Underline

Formatted: Underline

Formatted: Underline

Formatted: Underline

Formatted: Underline

Formatted: Underline

Formatted: Underline

Formatted: Font: Not Bold, Not Italic

Formatted: Font: Not Bold, Underline

Formatted: Superscript

Formatted: Superscript

NO_3^{-1} leaching from sandy soils are scanty. Therefore, the objective of the present study was~~aimed~~ to investigate the influence of bentonite on NO_3^{-1} leachability from a sandy soil after application of three N sources.

2. Materials and methods

2.1 Lysimeter experiment

A leaching experiment was conducted in the Soil Science Laboratory at COMSATS University Islamabad, Abbottabad Campus, Pakistan following the idea of Zhao et al. [45]. For this purpose, PVC columns, with 0.60 m length and 0.15 m diameter, were installed to run the experiment. The bottles used for collecting leachate were installed~~erected~~ on the floor. Filter papers were placed on porous bottom of the columns to prevent soil leaching. The columns were connected with bottles using small pipes for the collection of ~~to obtain~~ leachate. The connecting pipes were kept airtight to prevent evaporation~~transpiration~~ from the leachate bottles. Locally collected sandy soil samples from agricultural land (0-12 cm depth) were utilized for the experiment. Bentonite material was commercially purchased and then air-dried. Both the soil and bentonite were analyzed ~~pre-tested for chemical for physical and~~for physico-chemical properties before the experiment. After air drying, 46 kg of the soil was added to each column ~~leveling up to~~ (0.50 m length). The air-dried clay was applied at the rate of 0, 2 and 4% to the sandy soil packed in a PVC column. Three treatments of nitrogen (N) fertilizers namely calcium nitrate [$Ca(NO_3)_2$], ammonium chloride [NH_4Cl], and urea [$CO(NH_2)_2$] were applied to the soil. Based on the bulk density of soil (1.3 g cm^{-1}), each fertilizer was applied at 300 kg N ha^{-1}. Initially, as the soil was dry, the amount of first water application was kept higher so that enough water may~~is~~ drain~~ed~~ out to collect the leachate. For later leaching events, the leaching fraction (LF) was calculated by dividing the drained water by~~over~~ applied water. Then, the tap water was applied at leaching

Formatted: Superscript

Formatted: Superscript

Formatted: Underline

fraction of 0.3~0.4. Leachate was collected within 24 h after each water application. A total of five leaching events were covered, that is, 1st, 2nd, 4th, 6th and 10th day. The graphical display of the experiment is illustrated in [Figure 1~~Fig. 1~~].

Formatted: Underline

Formatted: Keep with next

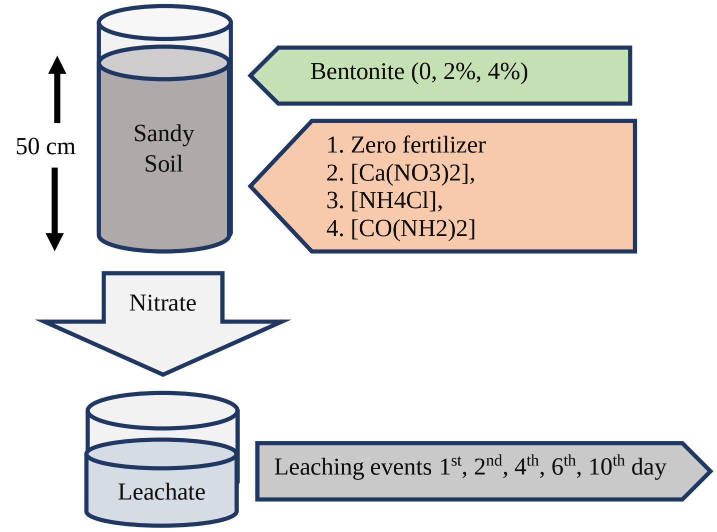

Figure 1 Graphical display of lysimeter experiment

~~Fig. 1 Graphical display of lysimeter experiment~~

Formatted: Font: (Default) Times New Roman, 12 pt, Not Italic

Formatted: Caption, Indent: First line: 0", Line spacing: single

Formatted: Font: (Default) Times New Roman, 12 pt

2.2 Laboratory analysis

~~A pre-experiment c~~Chemical analyse~~i~~s of soil, bentonite and tap water were~~as~~ carried out before experiment~~performed~~. Soil samples were air-dried and ~~strained~~sieved via ~~through~~ 2 mm sieve. The soil and air-dried bentonite material were tested for pH and electrical conductivity (EC) in 1:5 (w/v) soil-water suspensions by a pH meter (Model: HANNA HI 8520) and EC meter (Model: 4320 JENWAY), respectively [46]~~(Richard, 1954)~~. The soil was saturated for overnight, weighed and then the water holding capacity (WHC) of the soil was calculated by the difference

Formatted: Underline

in the weight of soil [47]. The bulk density of soil was determined using cylindrical cores. The soil sample was weighed and placed in the oven at 105℃ for 8 to 12 hours until the weight was constant. Bulk density was then calculated in the same way as described by Grossman and Reinsch [48](2002).

The post-experiment soil was sampled in two layers (0-25 and 25-50 cm) from the PVC columns and was thoroughly mixed. A 10 g soil sample was shaken in 100 mL distilled water for 1 h and then the suspension was filtered. Moisture content in the soil samples was adjusted by oven drying few grams of soil. The leachate collected from 5 events was analyzed for NO_3 concentration. The NO_3^{-1} concentration in pre- and post-experiment soil, bentonite and leachate was determined by UV spectrophotometer (Model: LI-UV-7000) at 220 nm [49](Norman and Stucki, 1981). All the reagents/chemicals of Sigma Aldrich, Germany, were utilized during the experiment.

2.3 Statistical analysis

Data were statistically analyzed by OriginLab 2021 for graphical presentation. The three-way analysis of variance (ANOVA) was performed on Sigmaplot. The three factors were taken as bentonite (0, 2% and 4%), N fertilizer sources ((0 fertilizer, [$Ca(NO_3)_2$], [NH_4Cl] and [$CO(NH_2)_2$])), and leaching events (1st, 2nd, 4th, 6th and 10th day) with three replications. A post hoc Tukey test was also performed to determine the significant difference between the levels of factors.

3. Results

3.1. Pre-experiment chemical analysis

Bentonite clay, soil and water were analyzed for chemical properties before experiment, which are presented in Table 1Table 1. Analysis revealed that soil had highest NO_3^{-1}

Formatted: Underline

Formatted: Underline

Formatted: Superscript

Formatted: Underline

Formatted: Underline

Formatted: Superscript

concentrations, compared to bentonite clay. The tap water had low concentrations of NO_3^{-1}. The electrical conductivity (EC) of bentonite was highest compared to soil and water samples, but still it fell below the category of non-saline (EC<4 dS m^{-1}). However, the pH of bentonite was lower (pH<7) making it more acidic as compared to soil and water.

~~**Table 1** Nitrate, electrical conductivity (EC) and pH of bentonite clay material, soil and tap water~~

Table 1 Nitrate, electrical conductivity (EC) and pH of bentonite clay material, soil and tap water

Material	Nitrate	EC (mS m^{-1})	pH
Bentonite	23.5 mg kg^{-1}	126.7	5.7
Soil	34.4 mg kg^{-1}	87.8	7.8
Tap water	7.8 mg L^{-1}	37.5	7.2

3.2. Effect of bentonite and N sources on NO_3^{-1} leaching

Impact of bentonite clay mixed with different N sources on NO_3^{-1} leaching is illustrated in [*Figure* 2~~Fig.2~~]. Results showed NO_3^{-1} concentration decreased by 12 to 19% in the leachate, irrespective of the source of N with increasing bentonite rates. The significantly highest reduction (20-25%) in NO_3^{-1} leaching was recorded with $Ca(NO_3)_2$ with 4% bentonite as compared with $CO(NH_2)_2$ and NH_4Cl at similar bentonite rates. At early stages of leaching, the leachate showed higher concentrations of NO_3^{-1} in leachate with both NH_4Cl and $Ca(NO_3)_2$, but leaching of NO_3 increased with urea sources at later leaching stages ~~(~~[Figure 3~~Fig. 3~~]~~)~~. ~~Fig 4. showed that t~~Total NO_3^{-1} loads were higher in soil with urea and $Ca(NO_3)_2$ treated soil at 4% bentonite as compared to NH_4Cl [Figure 4]. The incubation of soil with bentonite (4%) reduced NO_3 content by 7%, 20% and 8% with $Ca(NO_3)_2$, NH_4Cl and $CO(NH_2)_2$ treated soil, respectively ~~(~~[Figure 5~~Fig. 5~~]~~)~~.

Formatted: Superscript
Formatted: Font: (Default) Times New Roman, 12 pt, Bold, Not Italic
Formatted: Font: Bold, Not Italic
Formatted: Font: (Default) Times New Roman, 12 pt, Bold, Not Italic
Formatted: Caption
Formatted: Font: (Default) Times New Roman, 12 pt, Bold
Formatted: Superscript
Formatted: Superscript
Formatted: Underline
Formatted: Superscript
Formatted: Superscript
Formatted: Superscript
Formatted: Underline
Formatted: Underline
Formatted: Superscript
Formatted: Underline
Formatted: Underline

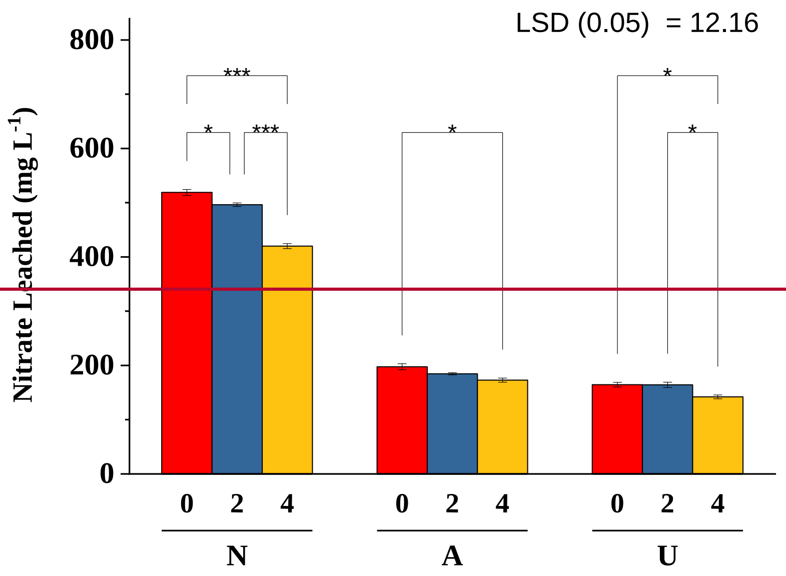

Figure 2 Average amount of leachate nitrate from bentonite treated sandy soil (from day 1 to day 10). N, A and U indicate $Ca(NO_3)_2$, NH_4Cl and $CO(NH_2)_2$, respectively. Bentonite clay was applied at the rate of 0%, 2% and 4%. *, ** and *** show significant differences at P<0.05, P<0,01 and P<0.001, respectively

Fig. 2 Average leachate nitrate from bentonite treated sandy soil (from day 1 to day 10). N, A and U indicate $Ca(NO_3)_2$, NH_4Cl and $CO(NH_2)_2$, respectively. Bentonite clay was applied at the rate of 0%, 2% and 4%.

Formatted: Keep with next

Formatted: Font: (Default) Times New Roman, 12 pt, Not Italic

Formatted: Caption, Indent: Left: 0", First line: 0"

Formatted: Font: (Default) Times New Roman, 12 pt, Not Italic, Subscript

Formatted: Font: (Default) Times New Roman, 12 pt, Not Italic

Formatted: Font: (Default) Times New Roman, 12 pt, Not Italic, Subscript

Formatted: Font: (Default) Times New Roman, 12 pt, Not Italic

Formatted: Font: (Default) Times New Roman, 12 pt, Not Italic, Subscript

Formatted: Font: (Default) Times New Roman, 12 pt, Not Italic

Formatted: Font: Not Italic, Subscript

Formatted: Font: (Default) Times New Roman, 12 pt, Not Italic

Formatted: Font: (Default) Times New Roman, 12 pt, Not Italic, Subscript

Formatted: Font: (Default) Times New Roman, 12 pt, Not Italic

Formatted: Font: Not Italic

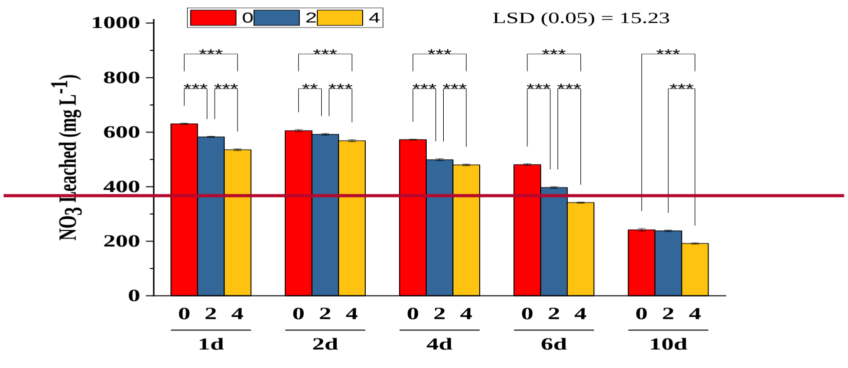

0
2
4
LSD (0.05) = 15.23
1000
800
600
400
200
0
NO3 Leached (mg L-1)
***
**
*
0 2 4
1d
2d
4d
6d
10d

(a)

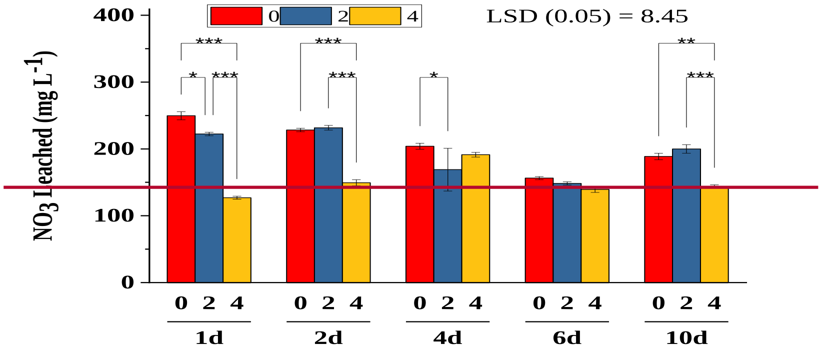

0
2
4
LSD (0.05) = 8.45
400
300
200
100
0
NO3 Leached (mg L-1)
***
**
*
0 2 4
1d
2d
4d
6d
10d

(b)

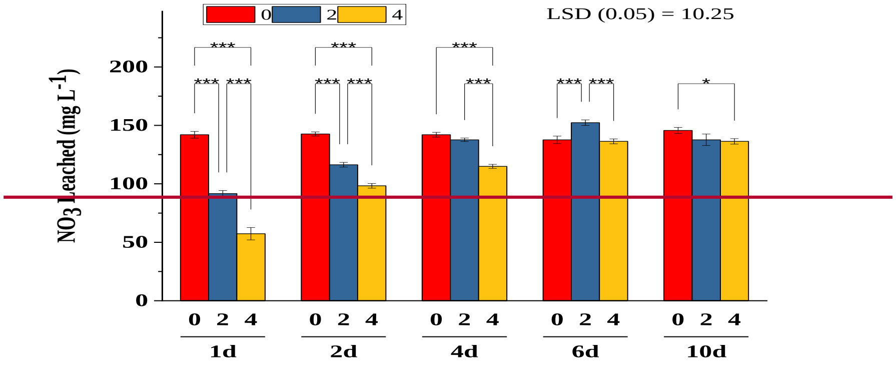

0
2
4
LSD (0.05) = 10.25
200
150
100
50
0
NO3 Leached (mg L-1)
***
*
0 2 4
1d
2d
4d
6d
10d

(c)

~~**Leaching events (Days)**~~

Figure 3 Nitrate leaching in bentonite amended sandy soil using $Ca(NO_3)_2$ (a), NH_4Cl (b), and $CO(NH_2)_2$ (c) with 0%, 2% and 4% bentonite during five leaching events (days). *, ** and *** show significant difference at $P<0.05$, $P<0,01$ and $P<0.001$, respectively

~~**Fig. 3** Nitrate leaching in bentonite amended sandy soil using $Ca(NO_3)_2$ (a), NH_4Cl (b), and $CO(NH_2)_2$ (c) during five leaching events~~

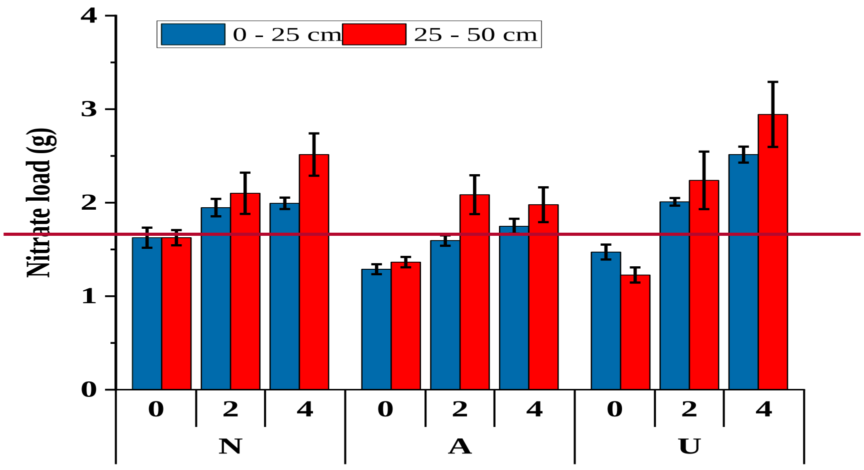

Figure 4 Residual nitrate loads (g) in soil after leaching events (days) at 0%, 2% and 4% bentonite with $Ca(NO_3)_2$ (N), NH_4Cl (A), and $CO(NH_2)_2$ (U). *, ** and *** show significant difference at $P<0.05$, $P<0,01$ and $P<0.001$, respectively

~~**Fig. 4** Residual nitrate loads (g) in soil after leaching events~~

Formatted: Keep with next

Formatted: Font: (Default) Times New Roman, 12 pt, Not Italic

Formatted: Font: (Default) Times New Roman, 12 pt, Not Italic, Subscript

Formatted: Font: (Default) Times New Roman, 12 pt, Not Italic

Formatted: Font: (Default) Times New Roman, 12 pt, Not Italic, Subscript

Formatted: Font: (Default) Times New Roman, 12 pt, Not Italic

Formatted: Font: (Default) Times New Roman, 12 pt, Not Italic, Subscript

Formatted: Font: (Default) Times New Roman, 12 pt, Not Italic

Formatted: Font: (Default) Times New Roman, 12 pt, Not Italic, Subscript

Formatted: Font: (Default) Times New Roman, 12 pt, Not Italic

Formatted ...

Formatted ...

Formatted ...

Formatted: Subscript

Formatted: Subscript

Formatted: Subscript

Formatted: Subscript

Formatted: Subscript

Formatted: Keep with next

Formatted ...

Formatted: Caption, Line spacing: single

Formatted ...

Formatted ...

Formatted ...

Formatted ...

Formatted ...

Formatted ...

Formatted ...

Formatted ...

Formatted ...

Formatted ...

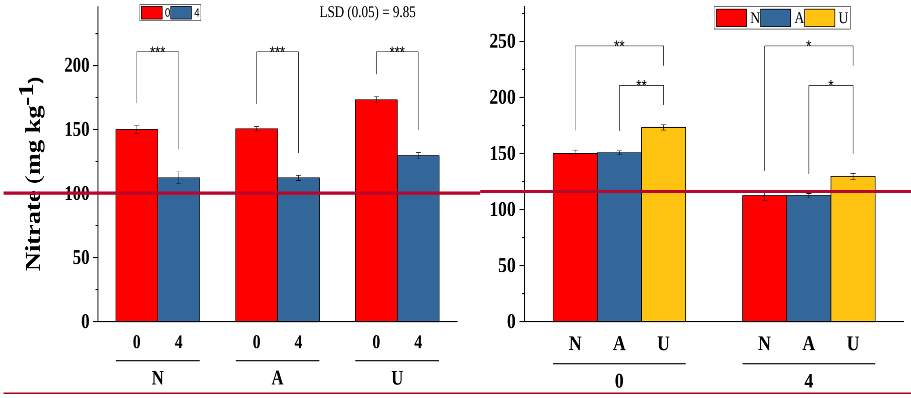

Figure 5 Nitrate concentration in 4% bentonite clay mixed sandy soil after discrete incubation with $Ca(NO_3)_2$ (N), NH_4Cl (A) and $CO(NH_2)_2$ (U), *, ** and *** show significant difference between treatments at P<0.05, P<0,01 and P<0.001, respectively

~~**Fig. 5** Nitrate concentration in 4% bentonite clay mixed sandy soil after discrete incubation with $Ca(NO_3)_2$ (N), NH_4Cl (A) and $CO(NH_2)_2$ (U)~~

Formatted: Keep with next

Formatted: Font: (Default) Times New Roman, 12 pt, Not Italic

Formatted: Font: (Default) Times New Roman, 12 pt, Not Italic, Subscript

Formatted: Font: (Default) Times New Roman, 12 pt, Not Italic

Formatted: Font: (Default) Times New Roman, 12 pt, Not Italic, Subscript

Formatted: Font: (Default) Times New Roman, 12 pt, Not Italic

Formatted: Font: (Default) Times New Roman, 12 pt, Not Italic, Subscript

Formatted: Font: (Default) Times New Roman, 12 pt, Not Italic

Formatted: Font: (Default) Times New Roman, 12 pt, Not Italic, Subscript

Formatted: Font: (Default) Times New Roman, 12 pt, Not Italic

Formatted: Font: (Default) Times New Roman, 12 pt, Not Italic, Subscript

Formatted: Font: (Default) Times New Roman, 12 pt, Not Italic

3.3. Statistical Analysis

A three-way analysis of variance (ANOVA) was performed on NO_3^{-1} leaching with 3~~4~~ N sources, 3 levels of bentonite and 5 leaching events ~~(~~[Table 2~~Table 2~~]~~)~~. Analysis showed that there was significant difference (P<0.001) within N sources, bentonite levels and leaching events (days). There was statistically significant (P<0.01) interaction among all the factors. The Tukey test revealed significant (P<0.05) difference between 0% and 2%, and 0% and 4% bentonite with all N sources on NO_3^{-1} leaching, but the difference was not significant between 2% and 4% bentonite with all N sources [Table 2].

~~**Table 2** Summary ANOVA on effect of bentonite and N sources on NO_3 leaching at different leaching events~~

Table 2 Summary ANOVA on effect of bentonite and N sources on NO_3^{-1} leaching at different leaching events

Source of Variation	DF	SS	MS	F	P
N sources (N)	2	2943336.13	1471668	14453	< 0.01
Bentonite levels (B)	2	74157.91	37078	364	< 0.01
Leaching event / day (D)	4	282061.82	70515	692	< 0.01
N x B	4	11270.48	2817	27	< 0.01
N x D	8	589355.42	73669	723	< 0.01
B x D	8	15955	1994	19	< 0.01
N x B x D	16	24125	1507	14	< 0.01
Residual	90	9164	101		
Total	134	3949426	29473		

4. Discussion

Formatted: Superscript

Formatted: Underline

Formatted: Superscript

Formatted: Underline

Formatted: Subscript

Formatted: Font: (Default) Times New Roman, 12 pt, Bold, Not Italic

Formatted: Font: (Default) Times New Roman, 12 pt, Bold, Not Italic

Formatted: Font: (Default) Times New Roman, 12 pt, Bold, Not Italic, Subscript

Formatted: Font: (Default) Times New Roman, 12 pt, Bold, Not Italic, Superscript

Formatted: Font: (Default) Times New Roman, 12 pt, Bold, Not Italic

Formatted: Caption, Justified, Keep with next

Formatted: Font: (Default) Times New Roman, 12 pt, Bold

Results showed that irrespective of the source of N, the NO_3^{-1} leaching consistently decreased with increasing bentonite application showing the sequences of fertilizer type as: $Ca(NO_3)_2$ > NH_4Cl > $CO(NH_2)_2$ ([*Figure 2*Fig. 2]). Form of N leached from the soil columns was closely related to the type of fertilizer applied to the soil [50](Zhou et al., 2006). The NO_3^{-1} fertilizers appeared to be more sensitive to the leaching, especially in sandy soils [51](De Boer et al., 2016) and also to the denitrification [52](Harty et al., 2016) as compared to urea or ammonium fertilizers. Also, NO_3^{-1} leaching is substantially higher in free-drained soils [53](De Boer et al., 2017), such as sandy soil with macropores used in the present study.

Application of bentonite decreased NO_3^{-1} leaching, regardless to the rate of application. The [NO_3^{-1}] decreased with increasing bentonite treatments level. Whereby higher NO_3^{-1} concentration was observed in NO_3^{-1} and NH_4 containing fertilizers during the initial leaching ([Figure 3Fig. 3]). Urea form of N showed consistent increases in NO_3^{-1} concentrations in water collected in later leaching stages ([Figure 3Fig. 3]). This could be associated with increased nitrification process in soils under unsaturated conditions [54](Zhang et al., 2004), which might have resulted in increased NO_3^{-1} leaching at later stages. Accumulation of NO_3^{-1} was more in the soil sampled from the lower layer of the column after a leaching process, showing the sequence as $CO(NH_2)_2$ > NH_4Cl > $Ca(NO_3)_2$.

The amount of total NO_3^{-1} retained in soil was termed as nitrate loads conversely related to the amount of NO_3^{-1} in the leachates ([Figure 4Fig. 4]). An enhanced application of bentonite significantly retained NO_3^{-1} in the soil columns. A higher amount of NO_3^{-1} was retained in the soil amended with 4% bentonite. The application of bentonite clay enhanced soil moisture and improved macro-aggregate development [55](Zhang et al., 2020) which improved soil quality through structural development, by increased exchange of anions and cations [56](Churchman et

Formatted: Superscript
Formatted: Underline
Formatted: Underline
Formatted: Superscript
Formatted: Underline
Formatted: Underline
Formatted: Superscript
Formatted: Underline
Formatted: Superscript
Formatted: Superscript
Formatted: Superscript
Formatted: Superscript
Formatted: Underline
Formatted: Superscript
Formatted: Underline
Formatted: Underline
Formatted: Superscript
Formatted: Superscript
Formatted: Superscript
Formatted: Superscript
Formatted: Underline
Formatted: Superscript
Formatted: Superscript
Formatted: Underline
Formatted: Underline

al., 2014) and helped in reduced leaching while promoting nutrient retention [57](Satje and Nelson, 2009).

Enrichment of sandy soils with bentonite increased the porosity and altered the pore-size distribution [58](Suzuki et al., 2007). The interactions of bentonite with biochar and urea improved soil properties by diffusing soil moisture which controlled the mobility of nutrients within soils [59](Liu et al., 2019), thus with high water retaining capacity, increased exchange capacity, swelling, thermal stability and slow-releasing characteristics, bentonite offers valuable solution to reduced nutrient leaching from loose soils.

Higher quantity of NO_3^{-1} was retained in urea treated soil followed by nitrate and ammonium containing fertilizers ([Figure 4Fig. 4]). This could be attributed to the transformation of NO_3^{-1} in urea contained soil after few days of incubation. Across all N sources, the application of bentonite (4%) markedly limited the release of NO_3^{-1}. After incubation, bentonite contents reduced the magnitude of NO_3^{-1} among fertilizers as follow: 7% in $Ca(NO_3)_2$, 20% in NH_4Cl and 8% in urea treated soil ([Figure 5Fig. 5]). The soil having negatively charged sites attracts more the positively charged NH_4 as compared to the negatively charged NO_3^{-1}, and therefore the NH_4 has been considered to be a less mobile in soils, than NO_3^{-1} [60, 61](Wang and Alva, 2000; Wang and Zhang, 2004).

The effect of bentonite on the leachability of NO_3^{-1} varied with different N fertilizers. The NO_3^{-1} leaching consistently decreased with increasing bentonite application showing the sequences of fertilizer type as: $Ca(NO_3)_2$ > NH_4Cl > $CO(NH_2)_2$ [Figure 2](Fig. 2). Addition of Ca as $Ca(NO_3)_2$ increased the adsorptive capacity of bentonite at low pH (5-6), while higher concentration of NO_3^{-1} ion due to the addition of calcium nitrate as N source resulted in increased adsorption of NO_3^{-1} at bentonite clay surfaces [62](Ouakouak et al., 2018). The mechanismprocess

Formatted: Underline
Formatted: Underline
Formatted: Underline
Formatted: Superscript
Formatted: Underline
Formatted: Superscript
Formatted: Superscript
Formatted: Superscript
Formatted: Underline
Formatted: Superscript
Formatted: Superscript
Formatted: Not Superscript/ Subscript
Formatted: Underline
Formatted: Underline
Formatted: Superscript
Formatted: Superscript
Formatted: Underline
Formatted: Superscript
Formatted: Superscript
Formatted: Underline

can be further explained by Ca hydrolysis, which resulted into the formation of less soluble $Ca(OH)_2$, releasing more H^+ ions, thus acidifying the media ([Eq. 1]) [63]. Under low pH, the anion exchange capacity of bentonites in significantly increased which offers more positive sites to attract NO_3^{-1} on its surface ([Eq. 2]), which increased the retention of NO_3^{-1} ions on soil colloids due to adsorption phenomenon ([Eq. 3]). This ultimately reduced NO_3^{-1} leaching from sandy soil and showed lower NO_3^{-1} concentrations in leachate. The entire process can be explained as follows, where A is dissociated anion and X is the soil colloidal surface

$$Ca^{2+} + H_2O \rightleftharpoons Ca(OH)_2 + 2H^+ \quad [Eq. 1]$$

$$X + H^+ \rightleftharpoons X^+ + HA \quad [Eq. 2]$$

$$X^+ + NO_3^- \rightleftharpoons XNO_3 \quad [Eq. 3]$$

Surface charge of variable charge clays varies with changes in pH of soil solution, therefore, at low pH, the anion exchange capacity exceeds cation exchange capacities which retain more NO_3^{-1} on its surfaces [64](Sollins et al., 1988), while increasing the mobility of NH_4 in such conditions. However, the mobility of both N forms can be maintained by adjusting the rates of $Ca(NO_3)_2$ and urea along with bentonite clay under field conditions. The study suggested that bentonite amendment with $Ca(NO_3)_2$ as N sources could effectively mitigate NO_3^{-1} leaching from sandy soils.

5. Conclusions

It is concluded that N sources and bentonite application were important factors affecting the NO_3^{-1} leaching from sandy soil. Application of N sources enhanced NO_3^{-1} leaching from the sandy soil. The NO_3^{-1} leaching decreased in the order of $Ca(NO_3)_2$ > NH_4Cl > urea. Bentonite substantially reduced NO_3^{-1} in the leachate. Urea showed higher NO_3^{-1} at the later leaching events.

Formatted: Underline
Formatted: Superscript
Formatted: Subscript
Formatted: Superscript
Formatted: Superscript
Formatted: Subscript
Formatted: Superscript
Formatted: Superscript
Formatted: Underline
Formatted: Subscript
Formatted: Superscript
Formatted: Superscript
Formatted: Superscript
Formatted: Superscript
Formatted: Superscript
Formatted: Superscript

Higher contents of NO_3^{-1} were retained in the soil with 4% bentonite. Higher NO_3^{-1} contents were accumulated in the lower part of the soil column after a leaching process. This experiment suggests that bentonite clay chemistry with added $Ca(NO_3)_2$ provide better understanding of anion exchange capacity to retain higher NO_3^{-1} concentrations in soil, thereby decreasing NO_3^{-1} leaching from sandy soil. Further research is suggested to investigate the effects of different clay types on the dynamics of nitrate under field conditions.

~~Acknowledgement~~

~~Authors appreciate the support of Yunnan Provincial Key Laboratory of Pollution Processes and Management in Plateau Lake Watershed (Project No: 202005AG070105) for funding this publication.~~

Authors Contributions

Z. Hussain., M. Irshad., R.A. Khattak. led the research study and prepared the manuscript. M. Mohiuddin., T. Cheng., C. Yao. analyzed the data and prepared graphs. D. Song. facilitated the research. R.A. Khattak edited the manuscript for technical inputs. All the authors discussed and reviewed the manuscript.

References

1. Elferink M, Schierhorn F (2016) Global demand for food is rising. Can we meet it? Harvard Business Review, Harward Business Publishing, Canada.
2. Bijay-Singh, Craswell E (2021) Fertilizers and nitrate pollution of surface and ground water: an increasing pervasive global problem. SN Applied Science 3: 518. https://doi.org/10.1007/s42452-021-04521-8

Formatted: Superscript

Formatted: Superscript

Formatted: Superscript

Formatted: Superscript

3. Shah AN, Iqbal J, Tanveer M et al. (2017) Nitrogen fertilization and conservation tillage: a review on growth, yield, and greenhouse gas emissions in cotton. Environmental Science and Pollution Research 24: 2261–2272. https://doi.org/10.1007/s11356-016-7894-4.

4. Mosongo PS, Pelster DE, Li X, Gaudel G, Wang Y, Chen S, et al. (2022) Greenhouse Gas Emissions Response to Fertilizer Application and Soil Moisture in Dry Agricultural Uplands of Central Kenya. Atmosphere 13: 463. https://doi.org/10.3390/atmos13030463

5. Houlton BZ, Almaraz M, Aneja V, Austin AT, Bai E, Cassman KG, et al. (2019) A world of cobenefits: Solving the global nitrogen challenge. Earth's Future 7: 865–872.

6. Xu P, Chen A, Houlton BZ, Zeng Z, Wei S, Zhao C, et al. (2020) Spatial Variation of Reactive Nitrogen Emissions from China's Croplands Codetermined by Regional Urbanization and Its Feedback to Global Climate Change. Geophysical Research Letters 47: e2019GL086551.

7. Pathak H, Jain N, Bhatia A, Kumar A, Chatterjee D, et al. (2016) Improved nitrogen management: A key to climate change adaptation and mitigation. Indian Journal of Fertilizers 12: 151–162.

8. Lu J, Bai Z, Velthof GL, Wu Z, Chadwick D, Ma L (2019) Accumulation and leaching of nitrate in soils in wheat-maize production in China. Agricultural Water Management 212: 407-415. https://doi.org/10.1016/j.agwat.2018.08.039

9. Kladivko EJ, Van Scoyoc GE, Monke EJ, Oates KM, Pask W (1991) Pesticides and nutrient movement into subsurface tile drains on s silt loam soil in Indiana. Journal of Environmental Quality 20: 264-270.

10. Jacinthe PA, Dick WA, Brown LC (1999) Bioremediation of nitrate-contaminated shallow soils using water table management techniques: Nitrate removal efficiency. Transactions of the American Society of Agricultural Engineers 42: 1251-1259.

11. Bibi S, Saifullah, Naeem A, Dahlawi S (2016) Environmental Impacts of Nitrogen Use in Agriculture, Nitrate Leaching and Mitigation Strategies. Hakeem et al. (eds.), Soil Science: Agricultural and Environmental Prospectives, Springer International Publishing Switzerland. https://doi.org/10.1007/978-3-319-34451-5_6

12. Tamagno S, Eagle AJ, McLellan E, van Kessel C, Linquist B A, Ladha JK et al. (2022) Quantifying N leaching losses as a function of N balance: A path to sustainable food supply chains. Agriculture, Ecosystems and Environment 324: 107714. https://doi.org/10.1016/j.agee.2021.107714

13. Hallaq AHA (2010) The impact of soil texture on nitrates leaching into groundwater in the North Governorate, Gaza Strip. Journal of Social Sciences 38(2): 1-37.

14. Plošek L, Elbl J, Lošák T, Kužel S, Kintl A, Juřička D et al. (2017) Leaching of mineral nitrogen in the soil influenced by addition of compost and N-mineral fertilizer. Acta Agriculturae Scandinavica, Section B. Soil and Plant Science 67(7): 607-614. https://doi.org/10.1080/09064710.2017.1322632

15. Huddell AM, Galford GL, Tully KL, Crowley C, Palm CA, Neill C et al. (2020) Meta-analysis on the potential for increasing nitrogen losses from intensifying tropical agriculture. Global Change Biology 26: 1668-1680. https://doi.org/10.1111/gcb.14951

16. Hatfield JL, Cambardella CA (2001) Nutrient management in cropping systems. In McFarland J and Sanderson M (ed.) Integrated management of land application of animal waste. (In press.). American Society of Agricultural Engineers, St. Joseph, MI.

17. Rasa K, Heikkinen J, Hannula M, Arstila K, Kulju S, Hyvaluoma J (2018) How and why does willow biochar increase a clay soil water retention capacity? Biomass and Bioenergy 119: 346-353. 10.1016/j.biombioe.2018.10.004

18. Wang H, Gao J-e, Li X-h, Zhang S-l, Wang H-j (2015) Nitrate Accumulation and Leaching in Surface and Ground Water Based on Simulated Rainfall Experiments. PLoS ONE 10(8): e0136274. https://doi.org/10.1371/journal.pone.0136274

19. Gatiboni L, Osmond D (2019) Nitrogen Management and Water Quality, Publication No: AG-439-02, NC State Extension Publications, NC State University, USA.

20. Johnson S, Burchell MR, Evans RO, Osmond DL, Gilliam JW (2012) Riparian Buffer Located in an Upland Landscape Position Does Not Enhance Nitrate-nitrogen Removal. Ecological Engineering 52: 252-261. http://dx.doi.org/10.1016/j.ecoleng.2012.11.006.

21. King SE, Osmond DL, Smith J, Dukes M, Evans RO, Knies S et al. (2015) Effects of Riparian Buffer Vegetation and Width: A 12-year Retrospective Study. *Journal of Environmental Quality* 45(4): 1243-1251. https://doi.org/10.2134/jeq2015.06.0321

22. Hansen B, Thorling L, Schullehner J et al. (2017) Groundwater nitrate response to sustainable nitrogen management. Scientific Reports 7: 8566. https://doi.org/10.1038/s41598-017-07147-2

23. Ayyub CM, Haidar MW, Zulfiqar F, Abideen ZU, Wright SR (2019) Potato tuber yield and quality in response to different nitrogen fertilizer application rates under two split doses in an irrigated sandy loam soil. Journal of Plant Nutrition 42(15): 1850-1860. https://doi.org/10.1080/01904167.2019.1648669

24. Alexander RB, Smith RA, Schwarz GE (1995) The regional transport of point and non-point source of nitrogen to the Gulf of Mexico. In Proc. Gulf of Mexico Hypoxia Conf.

Kenner LA, 5-6 Dec 1995. USEPA Publication National Center for Environment. Publ. and Inf. Washington, D.C.

25. Zeb S, Waseem A, Malik AH, Mahmood Q (2011) Water quality assessment of Siran River, Pakistan. International Journal of Physical Sciences 6: 7789 – 7798.

26. Kanwar RS, Stilenberg DER, Pfeiffer DL, Karlen TS, Simpkins WW (1993) Transport of nitrate and pesticides to shallow groundwater systems as affected by tillage and crop rotation practices, 270-273. In Proc. National Conference on Agricultural Research to Protect Water Quality.

27. Randall GW (1997) Nitrate-N in surface waters as influenced by climatic conditions and agricultural practices. In Proc. Agriculture. and Hypoxia in the Mississippi Watershed Conference, St. Louis, MO. 14–15 July 1997. American Farm Bureau Federation, Park Ridge, IL.

28. Cambardella CA, Moorman TB, Jaynes DB, Parkin TB, Karlen DL (1999) Water quality in Walnut Creek watershed: Nitrate nitrogen in soils, subsurface drainage water and shallow groundwater. Journal of Environmental Quality 28: 25-34.

29. EPA (2022) National Primary Drinking Water Regulations. Available at: https://www.epa.gov/ground-water-and-drinking-water/national-primary-drinking-water-regulations#Inorganic (Date of access: 28.08.2022).

30. Kross BC, Hallberg GR, Bruner R, Cherryholmes K, Johnson KJ (1993) The nitrate contamination of private well water in Iowa. American Journal of Public Health 83: 270-272.

31. WHO (2022) Guidelines for drinking-water quality 4th ed., Available at: https://www.who.int/publications/i/item/9789240045064 (Date of access: 28.08.2022).

32. Espejo-Herrera N et al. (2016) Colorectal cancer risk and nitrate exposure through drinking water and diet. International Journal of Cancer 139: 334–346. https://doil.org/10.1002/ijc.30083.

33. Jones RR et al. (2016) Nitrate from Drinking Water and Diet and Bladder Cancer Among Postmenopausal Women in Iowa. Environmental Health Perspectives 124: 1751–1758. https://doi.org/10.1289/EHP191.

34. Irshad M, Waseem A, Umar M, Sabir MA (2014) Leachability of nitrate from sandy soil using waste amendment. Communications in Soil Science and Plant Analysis 45: 680-687.

35. Sirivastava A, Singh PK (2017) Adsorption of Nitrate from Ground Water using Indian Bentonite: Fixed Bed Column Study. International Journal of Engineering Research and Technology (IJERT) 6(5): 390-394.

36. Bentahar S, Dnik A, Khomri ME, Messaoudi NE, Mohammad AD, Noureddine E et al. (2018) Removal of a cationic dye from aqueous solution by natural clay. Journal of Groundwater for Sustainable Development 6: 255–262. https://doi.org/10.1016/j.gsd.2018.02.002

37. De Castro MLFA, Abad MLB, Sumalinog DAG, Abarca RRM, Paoprasert P, de Luna MDG (2018) Adsorption of Methylene Blue dye and Cu(II) ions on EDTA-modified bentonite: Isotherm, kinetic and thermodynamic studies. J. Sustain. Environ. Res. 28, 197–205. https://doi.org/10.1016/j.serj.2018.04.001

38. Lopez-Fernandez M, Vilchez-Vargas R, Jroundi F, Boon N, Pieper D, Merroun ML (2018) Microbial community changes induced by uranyl nitrate in bentonite clay microcosms. Applied Clay Science 160: 206-216. https://doi.org/10.1016/j.clay.2017.12.034

39. Hall DJM, Jones HR, Crabtree WL, Daniels TL (2010) Claying and deep ripping can increase crop yields and profits on water repellent sands with marginal fertility in southern Western Australia. Australian Journal of Soil Research 48: 178–187. https://doi.org/10.1071/SR09078.

40. Mi J, Gregorich EG, Xu S, McLaughlin NB, Liu J (2020) Effect of bentonite as a soil amendment on field water‐holding capacity, and millet photosynthesis and grain quality. Scientific Reports 10: 18282. https://doi.org/10.1038/s41598-020-75350-9.

41. Czaban J, Siebielec G (2013) Effects of Bentonite on Sandy Soil Chemistry in a Long-Term Plot Experiment (II); Effect on pH, CEC, and Macro- and Micronutrients. Polish Journal of Environmental Studies 22(6): 1669-1676.

42. Zulfiqar F, Navarro M, Ashraf M, Akram NA, Munné-Bosch S (2019) Nanofertilizer use for sustainable agriculture: Advantages and limitations. Plant Science 289: 110270. https://doi.org/10.1016/j.plantsci.2019.110270.

43. Tahir S, Marschner P (2017) Clay Addition to Sandy Soil Reduces Nutrient Leaching—Effect of Clay Concentration and Ped Size. Communications in Soil Science and Plant Analysis 48(15): 1813–1821. https://doi.org/10.1080/00103624.2017.1395454.

44. Shi W, Ju Y, Bian R, Li L, Joseph S, Mitchell RG et al. (2020) Biochar bound urea boosts plant growth and reduces nitrogen leaching. Science of Total Environment 701: 134424. https://doi.org/10.1016/j.scitotenv.2019.134424

45. Zhao C, Hu C, Huang W, Sun X, Tan Q, Di H (2010) A lysimeter study of nitrate leaching and optimum nitrogen application rates for intensively irrigated vegetable production systems in Central China. Journal of Soils and Sediments 10: 9–17.

46. Richard LA (1954) Diagnosis and improvement of saline and alkali soils (USDA Handbook 60) Washington, DC: USDA.

47. Basso AS, Miguez FE, Laird DA, Horton R, Westgate M (2013) Assessing potential of biochar for increasing water-holding capacity of sandy soils. GCB-Bioenergy 5(2): 132-142. https://doi.org/10.1111/gcbb.12026

48. Grossman RB, Reinsch TG (2002) Bulk density and linear extensibility: core method. In: Dane JH, Topp GC (Eds.), Methods of Soil Analysis. Part 4. Physical Methods. Soil Science Society of America, Inc., Madison, WI, pp. 208–228.

49. Norman RJ, Stucki JW (1981) The determination of nitrate and nitrite in soil extracts by ultraviolet spectrophotometry. Soil Science Society of America 45: 347-353.

50. Zhou JB, Xi JG, Chen ZJ, Li SX (2006) Leaching and transformation of nitrogen fertilizers in soil after application of N with irrigation: A soil column method. Pedosphere 16(2): 245-252.

51. De Boer HC, Deru JGC, Hoekstra NJ, Van Eekeren N (2016) Strategic timing of nitrogen fertilization to increase root biomass and nitrogen-use efficiency of *Lolium perenne L.* Plant and Soil 407: 81-90.

52. Harty MA, Forrestal PJ, Watson CJ, McGeough KL, Carolan R, Elliot C et al. (2016) Reducing nitrous oxide emissions by changing N fertilizer use from calcium ammonium nitrate (CAN) to urea based formulations. Science of Total Environment 563-564: 576-586.

53. De Boer HC (2017) Nitrate leaching from liquid cattle manure compared to synthetic fertilizer applied to grassland or silage maize in the Netherlands. Wageningen Livestock Research Wageningen, October.

54. Zhang YM, Chen DL, Zhang JB, Edis R, Hu CS, Zhu AN (2004) Ammonia volatilization and denitrification losses from an irrigated maize-wheat rotation field in the North China Plain. Pedosphere 14(4): 533-540.

55. Zhang H, Chen W, Zhao B, Phillips LA, Zhou Y, Lapen DR et al. (2020) Sandy soils amended with bentonite induced changes in soil microbiota and fungistasis in maize fields. Applied Soil Ecology 146: 103378. https://doi.org/10.1016/j.apsoil.2019.103378.

56. Churchman GJ, Noble A, Bailey G, Chittleborough D, Harper R (2014) Clay addition and redistribution to enhance carbon sequestration in soils Soil Carbon. In: Hartemink AE, McSweeney K (eds.) Soil Carbon. Springer International Publishing, Cham, Switzerland, pp. 327-335. https://dx.doi.org/10.1007/978-3-319-04084-4_34 .

57. Satje A, Nelson PN (2009) Bentonite treatments can improve the nutrient and water holding capacity of sugarcane soils in the wet tropics. Proceedings: Australian Society of Sugarcane Technology 31: 166-176.

58. Suzuki S, Noble AD, Ruaysoongnern S, Chinabut N (2007) Improvement in water-holding capacity and structural stability of a sandy soil in northeast Thailand. Arid Land Research Management 21: 37-49.

59. Liu X, Liao J, Song H et al. (2019) A Biochar-Based Route for Environmentally Friendly Controlled Release of Nitrogen: Urea-Loaded Biochar and Bentonite Composite. Scientific Reports 9: 9548. https://doi.org/10.1038/s41598-019-46065-3.

60. Wang FL, Alva AK (2000) Ammonium adsorption and desorption in sandy soils. Soil Science Society of American Journal 64: 1669-1674.

61. Wang Y, Zhang YP (2004) Ammonium adsorption in a Eum-Orthic Anthrosol at different solution / soil ratios and temperature. Pedosphere 14(2): 253-257.

62. Ouakouak A, Youcef L, Achour S, Hamdi N (2018) Kinetics and equilibrium studies of nitrates adsorption onto natural Bentonite. Conference paper, Colloque International Sciences & Techniques de l'Eau & Environnement (STEE).

63. Lohinger H (2019) Calcium Hydroxide. Epina ebook Series. UC Davis, University of California, USA

64. Sollins P, Robertson GP, Uehara G (1988) Nutrient Mobility in Variable- and Permanent-Charge Soils. Biogeochemistry 6(3): 181-199.

~~Elferink, M, Schierhorn F., 2016. Global demand for food is rising. Can we meet it? Harvard Business Review. Harward Business Publishing, Canada.~~

~~Irshad, M., Waseem, A., Umar, M., Sabir, M.A., 2014. Leachability of nitrate from sandy soil using waste amendment. Commun Soil Sci Plant Anal. 45, 680-687.~~

~~Kladivko, E.J., Van Scoyoc, G.E., Monke, E.J., Oates, K.M., Pask, W., 1991. Pesticides and nutrient movement into subsurface tile drains on s silt loam soil in Indiana. J Environ Qual. 20, 264-270.~~

~~Jacinthe, P.A., Dick, W.A., Brown, L.C., 1999. Bioremediation of nitrate-contaminated shallow soils using water table management techniques: Nitrate removal efficiency. Trans ASAE, 42, 1251-1259.~~

~~Zeb, S., Waseem, A., Malik, A.H., Mahmood, Q., 2011. Water quality assessment of Siran River, Pakistan. Int'l. J. Phys. Sci. 6, 7789 – 7798.~~

~~Alexander, R.B., Smith, R.A., Schwarz, G.E., 1995.The regional transport of point and non-point source of nitrogen to the Gulf of Mexico. In Proc. Gulf of Mexico Hypoxia Conf. Kenner, L.A. 5-6 Dec 1995. USEPA Publ National Cent for Environ. Publ. and Inf. Washington, D.C.~~

Kanwar, R.S., Stilenberg, D.E.R., Pfeiffer, D.L., Karlen, T.S., Simpkins, W.W., 1993. Transport of nitrate and pesticides to shallow groundwater systems as affected by tillage and crop rotation practices, 270-273. In Proc. Natl. Conf. on Agric. Res. to Protect Water Quality.

Randall, G.W., 1997. Nitrate-N in surface waters as influenced by climatic conditions and agricultural practices. In Proc. Agric. and Hypoxia in the Mississippi Watershed Conf., St. Louis, MO. 14–15 July 1997. Am. Farm Bureau Federation, Park Ridge, IL.

Cambardella, C.A., Moorman, T.B., Jaynes, D.B., Parkin, T.B., Karlen, D.L., 1999. Water quality in Walnut Creek watershed: Nitrate-nitrogen in soils, subsurface drainage water and shallow groundwater. J Environ Qual. 28, 25-34.

Kross, B.C., Hallberg, GR., Bruner, R., Cherryholmes, K., Johnson, K.J., 1993. The nitrate contamination of private well water in Iowa. Am. J. Public Health 83, 270-272.

Bibi, S., Saifullah, Naeem, A., Dahlawi, S., 2016. Environmental Impacts of Nitrogen Use in Agriculture, Nitrate Leaching and Mitigation Strategies. Hakeem et al. (eds.), Soil Science: Agricultural and Environmental Prospectives, Springer International Publishing Switzerland. https://doi.org/10.1007/978-3-319-34451-5_6

Hallaq, A.H.A., 2010. The impact of soil texture on nitrates leaching into groundwater in the North Governorate, Gaza Strip. J. Social Sci. 38(2), 1-37.

Hatfield, J.L., Cambardella, C.A., 2001. Nutrient management in cropping systems. In J. McFarland and M. Sanderson (ed.) Integrated management of land application of animal waste. (In press.). Am. Soc. of Agric. Eng., St. Joseph, MI.

Sirivastava, A., Singh, P.K., 2017. Adsorption of Nitrate from Ground Water using Indian Bentonite: Fixed Bed Column Study. Int'l J. Eng. Res. Technol. (IJERT), 6(5), 390-394.

Bentahar, S. Dnik, A., Khomri, M.E., Messaoudi, N.E., Mohammad, A.D., Noureddine, E., Lacherai, A., 2018. Removal of a cationic dye from aqueous solution by natural clay. J. Groundw. Sustain. Devel, 6, 255–262. https://doi.org/10.1016/j.gsd.2018.02.002

De Castro, M.L.F.A., Abad, M.L.B., Sumalinog, D.A.G., Abarca, R.R.M., Paoprasert, P., de Luna, M.D.G., 2018. Adsorption of Methylene Blue dye and Cu(II) ions on EDTA-modified bentonite: Isotherm, kinetic and thermodynamic studies. J. Sustain. Environ. Res. 28, 197–205. https://doi.org/10.1016/j.serj.2018.04.001

Lopez-Fernandez, M., Vilchez-Vargas, R., Jroundi, F., Boon, N., Pieper, D., Merroun, M.L., 2018. Microbial community changes induced by uranyl nitrate in bentonite clay microcosms. Appl. Clay Sci. 160, 206-216. https://doi.org/10.1016/j.clay.2017.12.034

Hall, D. J. M., Jones, H.R., Crabtree, W.L., Daniels, T.L., 2010. Claying and deep ripping can increase crop yields and profits on water repellent sands with marginal fertility in southern Western Australia. J. Aust. J. Soil Res. 48, 178–187. https://doi.org/10.1071/SR09078

Mi, J., Gregorich, E.G. Xu, S., McLaughlin, N.B., Liu, J., 2020. Effect of bentonite as a soil amendment on field water‐ holding capacity, and millet photosynthesis and grain quality. Scientific Reports 10, 18282. https://doi.org/10.1038/s41598-020-75350-9

Czaban, J., Siebielec, G., 2013. Effects of Bentonite on Sandy Soil Chemistry in a Long-Term Plot Experiment (II); Effect on pH, CEC, and Macro- and Micronutrients. Pol. J. Environ. Stud. 22(6), 1669-1676.

Tahir, S., Marschner, P., 2017. Clay Addition to Sandy Soil Reduces Nutrient Leaching—Effect of Clay Concentration and Ped Size. Commun. Soil Sci. Plant Anal. 48(15), 1813–1821. https://doi.org/10.1080/00103624.2017.1395454

Shi, W., Ju, Y., Bian, R., Li, L., Joseph, S., Mitchell, R.G., Munroe, P., Taherymoosavi, S., Pan, G., 2020. Biochar bound urea boosts plant growth and reduces nitrogen leaching. Sci. Tot. Environ. 701, 134424. https://doi.org/10.1016/j.scitotenv.2019.134424

Richard, L.A. 1954. Diagnosis and improvement of saline and alkali soils (USDA Handbook 60) Washington, DC: USDA.

Norman, R.J., Stucki, J.W., 1981. The determination of nitrate and nitrite in soil extracts by ultraviolet spectrophotometry. Soil Sci Soc Am. 45, 347-353.

SAS, 1999. StatView reference, third edition, SAS Institute Inc., Cary, NC, USA.

Zhou, J.B., Xi, J.G., Chen, Z.J., Li, S.X., 2006. Leaching and transformation of nitrogen fertilizers in soil after application of N with irrigation: A soil column method. Pedosph. 16(2), 245-252.

De Boer, H.C., Deru, J.G.C., Hoekstra, N.J., Van Eekeren, N., 2016. Strategic timing of nitrogen fertilization to increase root biomass and nitrogen-use efficiency of Lolium perenne L. Plant Soil 407, 81-90.

Harty, M.A., Forrestal, P.J., Watson, C.J., McGeough, K.L., Carolan, R., Elliot, C., Krol, D.J., Laughlin, R.J., Richards, K.G., Lanigan, G.J., 2016. Reducing nitrous oxide emissions by changing N fertilizer use from calcium ammonium nitrate (CAN) to urea based formulations. Sci. Tot. Environ. 563-564, 576-586.

De Boer, H.C., 2017. Nitrate leaching from liquid cattle manure compared to synthetic fertilizer applied to grassland or silage maize in the Netherlands. Wageningen Livestock Research Wageningen, October.

Zhang, Y.M., Chen, D.L., Zhang, J.B., Edis, R., Hu, C.S., Zhu, A.N., 2004. Ammonia volatilization and denitrification losses from an irrigated maize-wheat rotation field in the North China Plain. Pedosph. 14(4), 533-540.

Zhang, H., Chen, W., Zhao, B., Phillips, L.A., Zhou, Y., Lapen, D.R., Liu, J., 2020. Sandy soils amended with bentonite induced changes in soil microbiota and fungistasis in maize fields. Appl. Soil Ecol. 146. https://doi.org/10.1016/j.apsoil.2019.103378

Churchman, G.J. Noble, A., Bailey, G., Chittleborough, D. Harper, R., 2014. Clay addition and redistribution to enhance carbon sequestration in soils Soil Carbon, Springer, Cham. 327-335.

Satje, A., Nelson, P.N., 2009. Bentonite treatments can improve the nutrient and water holding capacity of sugarcane soils in the wet tropics. Proceed Austr Soc Sugarcane Technol. 31, 166-176.

Ma, R.H.Y., Zong, T., Lu, S.G., 2012. Reducing bioavailability and leachability of copper in soils using coal fly ash, apatite, and bentonite. Commun Soil Sci Plant Anal. 43(15), 2004-2017.

Suzuki, S., Noble, A.D., Ruaysoongnern, S., Chinabut, N., 2007. Improvement in water-holding capacity and structural stability of a sandy soil in northeast Thailand. Arid Land Res Manage. 21, 37-49.

Liu, X., Liao, J., Song, H. et al. 2019. A Biochar-Based Route for Environmentally Friendly Controlled Release of Nitrogen: Urea-Loaded Biochar and Bentonite Composite. Sci, Rep. 9, 9548. https://doi.org/10.1038/s41598-019-46065-3

Wang, F.L., Alva, A.K., 2000. Ammonium adsorption and desorption in sandy soils. Soil Sci. Soc. Am. J. 64, 1669-1674.

Wang, Y., Zhang, Y.P., 2004. Ammonium adsorption in a Eum-Orthic Anthrosol at different solution / soil ratios and temperature. Pedosph. 14(2), 253-257.

Ouakouak, A., Youcef, L., Achour, S., Hamdi, N., 2018. Kinetics and equilibrium studies of nitrates adsorption onto natural Bentonite. Conference paper, Colloque International Sciences & Techniques de l'Eau & Environnement (STEE).

Sollins, P., Robertson, G.P., Uehara, G., 1988. Nutrient Mobility in Variable- and Permanent-Charge Soils. Biogeochem. 6(3), 181-199.

Grossman, R.B., Reinsch, T.G., 2002. Bulk density and linear extensibility: core method. In: Dane, J.H., Topp, G.C. (Eds.), Methods of Soil Analysis. Part 4. Physical Methods. Soil Science Society of America, Inc., Madison, WI, pp. 208–228

Supplementary File

Supplementary File
Bentonite experimer

Formatted: Font: (Default) Times New Roman, 12 pt, Bold

Field Code Changed

Formatted: Font: (Default) Times New Roman, 12 pt, Bold

Response to Reviewers

Reviewer #1:

Reviewed manuscript "Bentonite clay mixed with different N sources significantly reduce nitrate leaching from sandy soil" is an original and interesting study. Authors comprehensively evaluated the nitrate leaching in sandy soil that is beneficial for both environment and agriculture.

Authors are grateful to reviewer's encouraging remarks about the quality and significance of the current study. The responses are highlighted in red text.

I would suggest minor revision. Following are some suggestions for further improvements:

The manuscript was improved in accordance the suggestion of the reviewer

1. **From the title, I would suggest you to remove the words "mixed" and "significantly" to make it more catchy.**

   The title was modified as "Bentonite clay with different Nitrogen sources reduce nitrate leaching from sandy soil"

2. **In abstract results should be extended to few more lines.**

   A few ore significant results were added to abstract. See Line 39-40

3. **Journal format need to be followed regarding citations write up in text.**

   Citations were modified in accordance with journal format

4. **Introduction and Discussion section needs to further strengthen by latest studies on the subject.**

   Latest references were added to Introduction and Discussion to further strengthen the manuscript.

5. **Line 105: Company name and details for used material should incorporate**

   Details of the materials used are incorporated in Line 133 and 144.

6. **Line 106: for chemical for physical and chemical properties should be corrected**

   Corrected. See Line 115

7. **Line 120: Sentence not correct**

   Corrected. See Line 130

8. **Figure legends are too brief.**

   Figure legends only show the treatments. However, the details are further given in figure captions

9. **At some places in the text, there are grammatical mistakes that needs to be corrected by some native English colleague.**

   Grammatical mistakes were corrected English language was improved thought the manuscript

10. **To further strengthen introduction, following latest studies etc. are suggested to cite: https://doi.org/10.1016/j.plantsci.2019.110270 https://doi.org/10.1080/01904167.2019.1648669**

    Suggested studies are cited in the manuscript Introduction

Reviewer #2:
General remarks:

1. **The manuscript has an average quality of data. There must be some more experiments which supports different adsorption models to make the quality of this manuscript more better.**

   More experiments can not be added at this time of study. However, the authors have planned more experiments to involving adsorption studies for bentonite clay

2. **The introduction part has not enough citations which is necessary to support your statements of manuscript.**

   A few latest citations are added in Introduction

3. **Most of the references are old, there must be recent references from 2018 to onwards.**

   Latest references are added in the manuscript

4. **In the whole manuscript please replace NO_3 to NO_3^{-1}**

   NO_3 was replaced with NO_3^{-1} in the whole manuscript

5. **The discussion has been elaborated in a good way but it needs recent references.**

   Latest references are added in whole manuscript

6. **Format all the references in the reference list according to PLOS ONE journal. https://www.nlm.nih.gov/bsd/uniform_requirements.html**

   Reference format is changed in accordance with PLOSE ONE format.

Comment 1: please edit the title from "Bentonite clay mixed with different N sources significantly reduce nitrate leaching from sandy soil" to "Bentonite clay mixed with different nitrogen sources, significantly reduce nitrate leaching from sandy soil."

The title was modified according to the suggestions of the reviewers

Comment 2: line number 44 should be like "may lead to an increase in the use of fertilizers." Instead of "will/may lead to an increase in the use of fertilizers."

The sentence is corrected. See Line 48

Comment 3: in the start of line 47 there is no reference cited after the word atmosphere.

Reference is added to the same now in Line 53 with reference number [2, 3 4]

Comment 4: from line 50 to 53 "Developing an effective technology to retain nutrients in soils is imperative to prevent NO3 leaching from soils. Soil amendments have been considered as management practice to reduce NO3 losses from sandy soils (Irshad et al., 2014). Bentonite, an alumina-siliceous clay material, has not been previously utilized to control NO3 leaching."
There is no synchronization with the ongoing paragraph.

The paragraph was corrected and modified and the said statement was add in synchronization with third paragraph in Line 82-85 in Introduction

Comment 5: from line 47 to line 51 there is no single reference has been observed. Please support your statements with published research work.

These lines were modified and new references [2 - 8] were added with new statements. Check Lines 53 and onwards

Comment 6: in line 59 there must be root length instead of rooting length.

Rooting length was changed to root length. Check Line 63

Comment 7: line 77 to 78 there is no supporting reference has been found. Please write a proper reference.

The statement was modified and supported with reference [35]. See Lines 86-87.

Comment 8: from line 84 to 87, the author discussed about different amendments to control nitrogen leaching. Author must elaborate that how many percent the amendment improved the nitrogen leaching as compare to control treatment.
The percent reduction in nutrient leaching is given. See Line 93-97.

Comment 9: in the last paragraph of introduction from 92 to 96, there must be proper objectives of the aimed study. Please write it.

The objective of the current study is clearly mentioned in Line 103-105

Comment 10: in line 97 "Lysimeter experiment" has been described well but there is no supporting reference which describe that from where the author generated the idea.

Supporting reference describing the idea of Lysimetric experiment was add as Zhao et al [45] in Line 109

Comment 11: in Fig.1, author must use subscript letters for representing the chemical formulas e.g [CO(NH2)2] should be written as [CO(NH2)2] please do it for the rest also.

The subscript letters were used in all the formulas wherever required in Figures as well as text. See figures.

Comment 12: in the line 123, the author discussed about water holding capacity analysis but did not mentioned the reference at the end. Please cite a reference to support the analysis method.

Comment 13: why there are double brackets in line 137 and 138 "((0 fertilizer, [Ca(NO3)2], [NH4Cl] and [CO(NH2)2]))." Please remove them.

Double brackets are removed. See Line 150

Comment 14: from line 242 to 249 there is no supporting reference has been cited.

Reference [63] was added to the said statement. See Line 245-247

Comment 15: in the line 258, the NO3 should be written as NO3.

Subscript was added to the formula. See Line 261.

Reviewer #3:
I would suggest to authors to address following comments,

How different sources of N applied NO3 leaching from sandy soil?

Different sources of N such as calcium nitrate, ammonium chloride and urea were calculated according to 300 kg N per ha^{-1}. The bulk density and the weight of soil used for the experiment was calculated which further helped us to calculate the amount of each fertilizer as treatments.

What could be mechanistic explanation of this process.

The mechanism of the process for this experiment is explained in Line 245-258

Line 45-47, should be supported by doi.org/10.1007/s11356-016-7894-4

The statement is supported by references [2, 3, 4]. See Line 49-52

Significant amount of references are more than 15-20 years old, they need to be updated. For example, The United States Environmental Protection Agency set the standard for drinking water and health advisory level of 10 mg L-1 nitrate based on the human health risks (Kross et al., 1993), which is a very old figure.

New references from EPA and WHO are added to support this statement. See Line 78-80 with reference [29. 30. 31]

Authors did not develop a link between NO3 leaching and sandy soil, they have provided a generalized overview of NO3 leaching in soil, but did not explain in the context of sandy soil and why authors have used sandy soil in this study?

The factors explaining nitrate leaching from sandy soils are well explained in Line 58-69
