# Supplementary material for: Bentonite clay with different nitrogen sources can effectively reduce nitrate leaching from sandy soil
Source: PLoS One. 2022 Dec 22;17(12):e0278824. doi: 10.1371/journal.pone.0278824 (PMC9779043; doi:10.1371/journal.pone.0278824)
Supplement: Attachment — Submitted filename: Review Report.docx [file pone.0278824.s002.docx]

Reviewed manuscript “Bentonite clay mixed with different N sources significantly reduce1

nitrate leaching from sandy soil*”* is an original and interesting study. Authors comprehensively evaluated the nitrate leaching in sandy soil that is beneficial for both environment and agriculture. I would suggest minor revision.

Following are some suggestions for further improvements:

From the title, I would suggest you to remove the words “mixed” and “significantly” to make it more catchy.

In abstract results should be extended to few more lines.

Journal format need to be followed regarding citations write up in text.

Introduction and Discussion section needs to further strengthen by latest studies on the subject.

Line 105: Company name and details for used material should incorporate

Line 106: for chemical for physical and chemical properties should be corrected

Line 120: Sentence not correct

Figure legends are too brief.

At some places in the text, there are grammatical mistakes that needs to be corrected by some native English colleague.

To further strengthen introduction, following latest studies etc. are suggested to cite:

<https://doi.org/10.1016/j.plantsci.2019.110270>

<https://doi.org/10.1080/01904167.2019.1648669>
