# Supplementary material for: Bentonite clay with different nitrogen sources can effectively reduce nitrate leaching from sandy soil
Source: PLoS One. 2022 Dec 22;17(12):e0278824. doi: 10.1371/journal.pone.0278824 (PMC9779043; doi:10.1371/journal.pone.0278824)
Supplement: Attachment — Submitted filename: Response to Reviewers.docx [file pone.0278824.s003.docx]

**Response to Reviewers**

**Reviewer #1:**

**Reviewed manuscript “Bentonite clay mixed with different N sources significantly reduce
nitrate leaching from sandy soil” is an original and interesting study. Authors comprehensively evaluated the nitrate leaching in sandy soil that is beneficial for both environment and agriculture.**

Authors are grateful to reviewer’s encouraging remarks about the quality and significance of the current study. The responses are highlighted in red text.

**I would suggest minor revision. Following are some suggestions for further improvements:**

The manuscript was improved in accordance the suggestion of the reviewer

1. **From the title, I would suggest you to remove the words “mixed” and “significantly” to make it more catchy.**

The title was modified as “Bentonite clay with different Nitrogen sources reduce nitrate leaching from sandy soil”

2. **In abstract results should be extended to few more lines.**

A few ore significant results were added to abstract. See Line 39-40

3. **Journal format need to be followed regarding citations write up in text.**

Citations were modified in accordance with journal format

4. **Introduction and Discussion section needs to further strengthen by latest studies on the subject.**

Latest references were added to Introduction and Discussion to further strengthen the manuscript.

5. **Line 105: Company name and details for used material should incorporate**

Details of the materials used are incorporated in Line 133 and 144.

6. **Line 106: for chemical for physical and chemical properties should be corrected**

Corrected. See Line 115

7. **Line 120: Sentence not correct**

Corrected. See Line 130

8. **Figure legends are too brief.**

Figure legends only show the treatments. However, the details are further given in figure captions

9. **At some places in the text, there are grammatical mistakes that needs to be corrected by some native English colleague.**

Grammatical mistakes were corrected English language was improved thought the manuscript

10. **To further strengthen introduction, following latest studies etc. are suggested to cite:**
    [**https://doi.org/10.1016/j.plantsci.2019.110270**](https://doi.org/10.1016/j.plantsci.2019.110270)

[**https://doi.org/10.1080/01904167.2019.1648669**](https://doi.org/10.1080/01904167.2019.1648669)

Suggested studies are cited in the manuscript Introduction

**Reviewer #2:**

**General remarks:**

1. **The manuscript has an average quality of data. There must be some more experiments which supports different adsorption models to make the quality of this manuscript more better.**

More experiments can not be added at this time of study. However, the authors have planned more experiments to involving adsorption studies for bentonite clay

2. **The introduction part has not enough citations which is necessary to support your statements of manuscript.**

A few latest citations are added in Introduction

3. **Most of the references are old, there must be recent references from 2018 to onwards.**

Latest references are added in the manuscript

4. **In the whole manuscript please replace NO_3_ to NO_3_^−1^**

NO_3_ was replaced with NO_3_^-1^ in the whole manuscript

5. **The discussion has been elaborated in a good way but it needs recent references.**

Latest references are added in whole manuscript

6. **Format all the references in the reference list according to PLOS ONE journal.** [**https://www.nlm.nih.gov/bsd/uniform_requirements.html**](https://www.nlm.nih.gov/bsd/uniform_requirements.html)

Reference format is changed in accordance with PLOSE ONE format.

**Comment 1: please edit the title from “Bentonite clay mixed with different N sources significantly reduce nitrate leaching from sandy soil” to “Bentonite clay mixed with different nitrogen sources, significantly reduce nitrate leaching from sandy soil.”**

The title was modified according to the suggestions of the reviewers

**Comment 2: line number 44 should be like “may lead to an increase in the use of fertilizers.” Instead of “will/may lead to an increase in the use of fertilizers.”**

The sentence is corrected. See Line 48

**Comment 3: in the start of line 47 there is no reference cited after the word atmosphere.**

Reference is added to the same now in Line 53 with reference number [2, 3 4]

**Comment 4: from line 50 to 53 “Developing an effective technology to retain nutrients in soils is imperative to prevent NO3 leaching from soils. Soil amendments have been considered as management practice to reduce NO3 losses from sandy soils (Irshad et al., 2014). Bentonite, an alumina-siliceous clay material, has not been previously utilized to control NO3 leaching.”**

**There is no synchronization with the ongoing paragraph.**

The paragraph was corrected and modified and the said statement was add in synchronization with third paragraph in Line 82-85 in Introduction

**Comment 5: from line 47 to line 51 there is no single reference has been observed. Please support your statements with published research work.**

These lines were modified and new references [2 - 8] were added with new statements. Check Lines 53 and onwards

**Comment 6: in line 59 there must be root length instead of rooting length.**

Rooting length was changed to root length. Check Line 63

**Comment 7: line 77 to 78 there is no supporting reference has been found. Please write a proper reference.**

The statement was modified and supported with reference [35]. See Lines 86-87.

**Comment 8: from line 84 to 87, the author discussed about different amendments to control nitrogen leaching. Author must elaborate that how many percent the amendment improved the nitrogen leaching as compare to control treatment.**

The percent reduction in nutrient leaching is given. See Line 93-97.

**Comment 9: in the last paragraph of introduction from 92 to 96, there must be proper objectives of the aimed study. Please write it.**

The objective of the current study is clearly mentioned in Line 103-105

**Comment 10: in line 97 “Lysimeter experiment” has been described well but there is no supporting reference which describe that from where the author generated the idea.**

Supporting reference describing the idea of Lysimetric experiment was add as Zhao et al [45] in Line 109

**Comment 11: in Fig.1, author must use subscript letters for representing the chemical formulas e.g [CO(NH2)2] should be written as [CO(NH2)2] please do it for the rest also.**

The subscript letters were used in all the formulas wherever required in Figures as well as text. See figures.

**Comment 12: in the line 123, the author discussed about water holding capacity analysis but did not mentioned the reference at the end. Please cite a reference to support the analysis method.**

**Comment 13: why there are double brackets in line 137 and 138 “((0 fertilizer, [Ca(NO3)2], [NH4Cl] and [CO(NH2)2])).” Please remove them.**

Double brackets are removed. See Line 150

**Comment 14: from line 242 to 249 there is no supporting reference has been cited.**

Reference [63] was added to the said statement. See Line 245-247

**Comment 15: in the line 258, the NO3 should be written as NO3.**

Subscript was added to the formula. See Line 261.

**Reviewer #3:**

**I would suggest to authors to address following comments,**

**How different sources of N applied NO3 leaching from sandy soil?**

Different sources of N such as calcium nitrate, ammonium chloride and urea were calculated according to 300 kg N per ha^-1^. The bulk density and the weight of soil used for the experiment was calculated which further helped us to calculate the amount of each fertilizer as treatments.

**What could be mechanistic explanation of this process.**

The mechanism of the process for this experiment is explained in Line 245-258

**Line 45-47, should be supported by doi.org/10.1007/s11356-016-7894-4**

The statement is supported by references [2, 3, 4]. See Line 49-52

**Significant amount of references are more than 15-20 years old, they need to be updated.**

**For example, The United States Environmental Protection Agency set the standard for drinking water and health advisory level of 10 mg L-1 nitrate based on the human health risks (Kross et al., 1993), which is a very old figure.**

New references from EPA and WHO are added to support this statement. See Line 78-80 with reference [29. 30. 31]

**Authors did not develop a link between NO3 leaching and sandy soil, they have provided a generalized overview of NO3 leaching in soil, but did not explain in the context of sandy soil and why authors have used sandy soil in this study?**

The factors explaining nitrate leaching from sandy soils are well explained in Line 58-69
