# Supplementary material for: Bentonite clay with different nitrogen sources can effectively reduce nitrate leaching from sandy soil
Source: PLoS One. 2022 Dec 22;17(12):e0278824. doi: 10.1371/journal.pone.0278824 (PMC9779043; doi:10.1371/journal.pone.0278824)
Supplement: Attachment — Submitted filename: Response to Reviewers.docx [file pone.0278824.s005.docx]

**Response to Reviewers**

The authors appreciate the positive and encouraging response of reviewers regarding the quality and significance of the manuscript. However, minor corrections have been suggested by Reviewer#2 which have been incorporated in the manuscript and the response is highlighted in red.

The Title has also been modified. See if appropriate.

2. Is the manuscript technically sound, and do the data support the conclusions?

The manuscript must describe a technically sound piece of scientific research with data that supports the conclusions. Experiments must have been conducted rigorously, with appropriate controls, replication, and sample sizes. The conclusions must be drawn appropriately based on the data presented.

Reviewer #2: Partly

Reviewer #3: Yes

In reviewers’ response to the question, the authors are convinced that manuscript describes technically sound study. The experiment was conducted with appropriate control, replications and appropriate sample size. This provided the basis for appropriate statistical analysis and the conclusion was drawn on the basis of the result findings. The conclusion seems okay.

**Specific comments and response to reviewers**

**Reviewer #2:**

line no 105: please write here three sources names in objectives.

The three N sources, calcium nitrate [Ca(NO_3_)_2_], ammonium chloride [NH_4_Cl], and urea [CO(NH_2_)_2_] were added in objectives. See Line 105.

Line 32: The effects of bentonite clay on NO3 -1leaching from sandy soils are not
extensively studied. Author is sure that he is not using the sandy soil?

The sentence was modified. However, the word “extensively’ is used along with not, which means to say that although some work has been done using bentonite but no extensive studies have been performed to evaluate the impact of bentonite using three N sources in sandy soils on leaching of NO_3_^-1^

Line 55: The N fertilizers, used either as urea or ammonium form, is biochemically converted to NO3-1 which is susceptible to leaching from soil-plant system and enter groundwater bodies.
Please rewrite the line by replacing is with are.

The word “is” was replaced by “are” in the sentence. See line 55

Line 61, 73: NO3 should be replaced with NO3-1.

NO_3_ was replaced by NO_3_^-1^. See Lines 61, 73

**Reviewer #3:**

Authors have revised the manuscript and can be accepted in current form for publication. This is a very interested study and have showed the combination of Bentonite clay with different nitrogen sources to reduce N leaching from sandy soils.

The reviewer already recommended the manuscript as Accepted.
